# Toward a unified data-driven turbulence model through multi-objective learning

**DOI:** 10.1093/nsr/nwag344

**Published:** 2026-06-04

**Authors:** Zhuoran Liu, Haochen Wang, Zhuolin Zhao, Heng Xiao

**Affiliations:** Stuttgart Center for Simulation Science, University of Stuttgart, Stuttgart 70569, Germany; Institute of Aerospace Thermodynamics, University of Stuttgart, Stuttgart 70569, Germany; Stuttgart Center for Simulation Science, University of Stuttgart, Stuttgart 70569, Germany; Institute of Aerospace Thermodynamics, University of Stuttgart, Stuttgart 70569, Germany; Stuttgart Center for Simulation Science, University of Stuttgart, Stuttgart 70569, Germany; Institute of Aerospace Thermodynamics, University of Stuttgart, Stuttgart 70569, Germany; Stuttgart Center for Simulation Science, University of Stuttgart, Stuttgart 70569, Germany; Institute of Aerospace Thermodynamics, University of Stuttgart, Stuttgart 70569, Germany

**Keywords:** Reynolds-averaged Navier–Stokes, unified turbulence model, multi-objective learning

## Abstract

Turbulence remains one of the last unresolved problems of classical physics and a major bottleneck to accurate flow prediction in climate, aerospace, and energy systems. Industrial simulations therefore rely on averaged representations of turbulence, which often struggle to predict flows governed by multiple interacting mechanisms. We present a unified, data-driven turbulence modeling framework designed to learn robustly from sparse, indirect observations across diverse flow regimes. The framework embeds physical consistency into a flexible, frame-invariant closure, automatically selects representative training cases based on similarity of flow-feature distributions, and learns a single, unified model through a multi-objective ensemble strategy that balances competing objectives across flows and quantities of interest. The resulting unified foundation model adapts seamlessly across regimes without manual intervention. It outperforms existing turbulence models across a broad spectrum of canonical flows and maintains improved performance in complex three-dimensional configurations of industrial relevance, including a gas turbine diffuser, a generic car, and a generic aircraft. When application-specific accuracy is required, the framework further enables specialist models through additive fine-tuning on targeted flow datasets. The results demonstrate the feasibility of a deployable and generalized turbulence modeling approach that unifies multiple flow mechanisms within a single architecture for a broad range of natural and industrial flows.

## Teaser

We present a unified data-driven turbulence model that ensures physical consistency and generalizes across diverse regimes using sparse data. It outperforms existing models in canonical and industrial configurations without manual tuning, offering a deployable architecture for accurate flow prediction.

## INTRODUCTION

Turbulence remains a central challenge in classical physics [1]. Its presence is ubiquitous in natural and engineered flows from sub-meter to planetary scales. Accurate prediction of turbulent flows has far-reaching impacts on modern society, ranging from reducing uncertainties in climate projections and improving fidelity in extreme weather events [2] to designing safer and more efficient mission-critical systems such as aircraft engines and power plants [3]. The chaotic and multi-scale nature of turbulence [4] is responsible for critical physics in these systems, such as momentum and heat transport in planetary boundaries and flow through engine turbines and pollutant transport in rivers and oceans. Despite the importance of turbulent transport in such processes, our current modeling of such effects in state-of-the-art simulations is still crude. For example, in weather forecast models, the vertical turbulent mixing is still represented as one-dimensional parameterization schemes known as the planetary boundary layer scheme [5]. Similarly, in industrial computational fluid dynamics, Reynolds-averaged Navier–Stokes (RANS) solvers are still the workhorse tool for routine simulations [6], where the RANS equations govern the mean flow field and the “averaged effects” of the turbulent fluctuations, introduced through averaging as the Reynolds stress, are represented with turbulence models [7,8]. This is because such applications require either long-time integration or many simulations for design optimization or uncertainty quantification. It becomes prohibitively expensive to fully resolve all the turbulence scales (as in direct numerical simulations) or even to partially resolve them (as in large eddy simulations). Despite the growth of computational resources, flow solvers based on pure modeling or parameterization of the turbulence (referred to as turbulence modeling collectively hereafter) will remain as the backbone in many fields in the years to come. Therefore, the theoretical foundation and practical development of turbulence will continue to have societal and scientific importance.

### Pursuit of a universal constitutive relation

More than half a century ago, Lumley [9] postulated the existence of a turbulent constitutive relation that maps the mean velocities to the Reynolds stresses. From a phenomenological perspective, such a relation shall be inferred from data and constrained by mathematical invariance and symmetry, which in principle enables transferability across similar flows. Mathematically, it defines the Reynolds stress at a given location as a functional of the mean velocity field evaluated over a surrounding neighborhood, which may in the limiting case reduce to a purely local dependence. Using experimental data, Lumley established that such a local constitutive relation exists for homogeneous shear flows and homogeneous strain, both of which behave analogously to viscoelastic fluids with an increasing relaxation timescale [9]. Despite its conceptual depth, this hypothesis has had limited impact on the subsequent development of turbulence modeling. Although numerous turbulence models have since been proposed, none has achieved the universality across a broad range of flows envisioned by Lumley. Recent analyses have further emphasized fundamental limitations of single-point, local closures in representing non-local transport and non-equilibrium effects in turbulence [10], as purely local constitutive relations cannot capture the history dependence and spatial interactions inherent in turbulent flows. Consequently, the existence of a universal, local constitutive relation remains uncertain under practical modeling constraints.

### From universality to unification

Rather than pursuing a universal constitutive relation of uncertain existence, this work focuses on developing a unified turbulence model that can represent multiple flow mechanisms within a single framework. Here, a unified model denotes a closure capable of handling multiple flow mechanisms, such as attached boundary layers, separated flows, and secondary flows, without manual zoning, switching, or blending [11,12]. Such unification is particularly attractive for industrial applications such as aircraft aerodynamics and turbine flows, where multiple flow mechanisms coexist, interact, and cannot be clearly separated spatially. Developing a unified foundation model of this type is the central objective of this work. Nevertheless, even a more limited degree of unification, referred to here as a specialist model, would already offer substantial practical value [13,14].

### Efforts toward a unified turbulence model

Achieving a unified turbulence model relies on training with diverse flows that capture multiple flow mechanisms. A number of strategies have been pursued with varying degrees of success. For example, multi-case training using symbolic model representations has produced models that perform well across several wall-bounded flows, representing some of the earliest steps toward unified turbulence modeling [15,16]. Nevertheless, the restrictive functional forms and reliance on derivative-free optimization can limit scalability, particularly when many competing objectives arise. An alternative strategy, which we refer to as “unification through aggregation”, relies on expert models tailored to individual flow mechanisms, with a classifier assigning one or more models to each spatial location during prediction [17,18]. Such approaches, however, require multiple model evaluations and can struggle when different flow mechanisms strongly interact within the same region. Yet, another strategy toward a unified model is to provide users with a generalized model that can be tuned for individual flows, exemplified by the generalized *k*–*ω* model (GEKO) [19]. This model relies on manual tuning for different flows, which limits its ability to achieve true unification. Similar efforts toward a generalizable closure have also emerged in wall-modeled large eddy simulations, such as the knowledge-integrated additive learning [20] and building block approaches [21]. However, to this day, a unified RANS turbulence model that can seamlessly handle coexisting, vastly different flow mechanisms in a single flow (for example, flows with massive separation and swirling simultaneously) does not yet exist. The difficulty lies in the fact that different flow regimes impose conflicting requirements on the model: adjustments that improve one regime can degrade another. This conflict fundamentally limits the transferability of existing models across diverse flow regimes. As noted in the NASA Langley Turbulence Modeling Symposium [11], after a decade of intense research, the community has yet to produce a data-driven turbulence model that exceeds current models in terms of predictability, generality, and robustness.

### Unification via multi-objective learning

**Figure 1.**
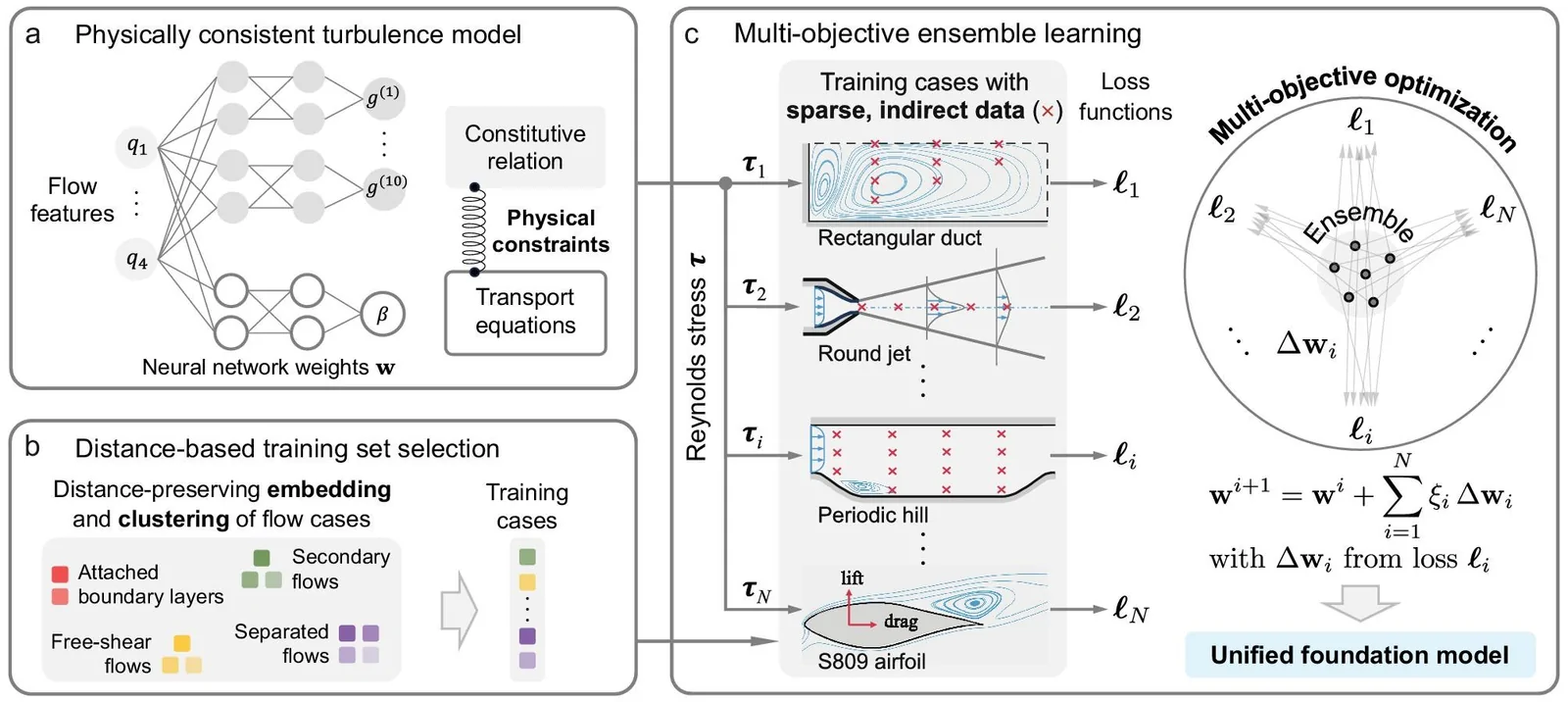
The proposed framework proceeds through three steps to construct a unified turbulence model. The learning is formulated as a multi-objective optimization problem, yielding a single neural-network-based model that reconciles competing objectives across flows and quantities of interest. (a) First, a physically consistent and frame-invariant model representation is employed, in which the turbulent constitutive relation and transport equations are learned in a coupled and internally consistent manner under physical constraints, enabling a single model to adapt to different flow mechanisms without manual switching. (b) Second, a comprehensive dataset of flows is compiled, and a distance-based training-set selection strategy is used to automatically identify a compact and representative set of training cases by comparing probability distributions of local, frame-invariant flow features, thereby spanning relevant flow physics while avoiding redundancy. (c) Finally, an ensemble-based, multi-objective learning framework is applied to learn a unified model from diverse flows and sparse, indirect observations, balancing competing objectives across flows and quantities of interest. Taken together, these three components yield a unified foundation turbulence model that captures multiple flow regimes within a single set of learned network weights. For application-specific accuracy, the unified foundation model can be adapted into a specialist model through additive fine-tuning (see Fig. S1 in Supplementary Material).

In this work, we develop a unified data-driven turbulence modeling framework that learns a single closure capable of representing multiple flow mechanisms. The unification is achieved through a strategy that balances conflicting objectives and branches across non-conflicting ones. As illustrated in Fig. 1, the proposed machine-learning framework proceeds through three steps that together yield a unified turbulence model. First, we employ a physically consistent, frame-invariant model representation that enables a single turbulence model to adapt to different flow mechanisms without manual switching (Fig. 1a) [22,23]. Second, we use a distance-based selection strategy to automatically identify a compact and representative set of training flows that spans the relevant flow physics while avoiding redundancy (Fig. 1b). Finally, we devise an ensemble-based, multi-objective learning strategy to balance competing objectives across flow regimes and, through this process, learn a unified turbulence model from heterogeneous flows and sparse, indirect observations [24] (Fig. 1c). The resulting foundation model captures multiple flow mechanisms within a single set of network weights and is seamlessly integrated into a Reynolds-averaged Navier–Stokes solver in OpenFOAM.

Building on prior work that enables learning a turbulence model from indirect data [24], this study introduces several advances that together enable robust unification across flow regimes. First, we develop a flexible and physically consistent turbulence model representation in which the turbulent constitutive relation and transport equations are learned in a coupled manner, ensuring coherent closure behavior and turbulence scales [23]. Second, we introduce an automatic, distribution-based strategy for selecting representative training flows, coupled with the compilation of a comprehensive library of 36 canonical-to-complex flows—the most extensive dataset used to train a single turbulence model to date. Finally, and most importantly, we formulate turbulence model learning as a multi-objective optimization problem, allowing competing objectives arising from different flows and quantities of interest to be reconciled within a single unified model.

In particular, this formulation naturally accommodates multiple quantities of interest from the same flow (for example, drag and lift for an airfoil), which can introduce competing demands on the turbulence model (see Section S3.2.2).

#### Flexible and physically consistent model representation

We construct a turbulence model that combines high expressive power with physical consistency and numerical robustness by embedding invariance, structural hierarchy, and physics-based constraints directly into the model representation (see Fig. 1a). The tensor basis neural network (TBNN) framework [22] provides a flexible and frame-invariant realization of the general eddy-viscosity model, making it well suited for unified turbulence modeling across diverse flow regimes. In its standard form, the TBNN predicts coefficients for tensor bases of all polynomial orders through a single, shared network, implicitly weighting linear and higher-order nonlinear terms equally. This often introduces unnecessary nonlinearity that degrades generalization and numerical stability. To impose a clear physical structure, we introduce a parallel TBNN architecture (see Fig. S2b in Supplementary Material), in which shared invariant features are mapped to separate, order-specific network branches that predict coefficients for low-order and higher-order tensor bases independently, enabling physics-informed regularization of nonlinear contributions. Beyond the constitutive relation alone, turbulence modeling requires internal consistency with the turbulence transport equations that determine turbulent time and length scales. Rather than modifying either component in isolation [22], we treat coefficients in both the constitutive relation and the transport equations as learnable fields within a unified network architecture and optimize them in a coupled manner [23]. Finally, we impose physics-based constraints to ensure correct behavior in canonical limits, including decaying homogeneous isotropic turbulence, equilibrium homogeneous shear, compatibility with the logarithmic law of the wall, and edge of turbulence region [12,25]. Together, these design choices establish a turbulence model representation that is flexible yet structured, enforces internal consistency between constitutive and transport closures, and embeds physical constraints to ensure correct behavior in canonical flows. Despite these improvements, the model remains a single-point, local closure and thus inherits fundamental limitations. Such closures approximate inherently multi-point and non-local turbulence dynamics using local flow quantities and cannot distinguish between different flow structures that produce similar Reynolds stresses [26]. The present work aims to improve predictive accuracy within this framework rather than to overcome these intrinsic limitations.

#### Distribution-based training set selection

**Figure 2.**
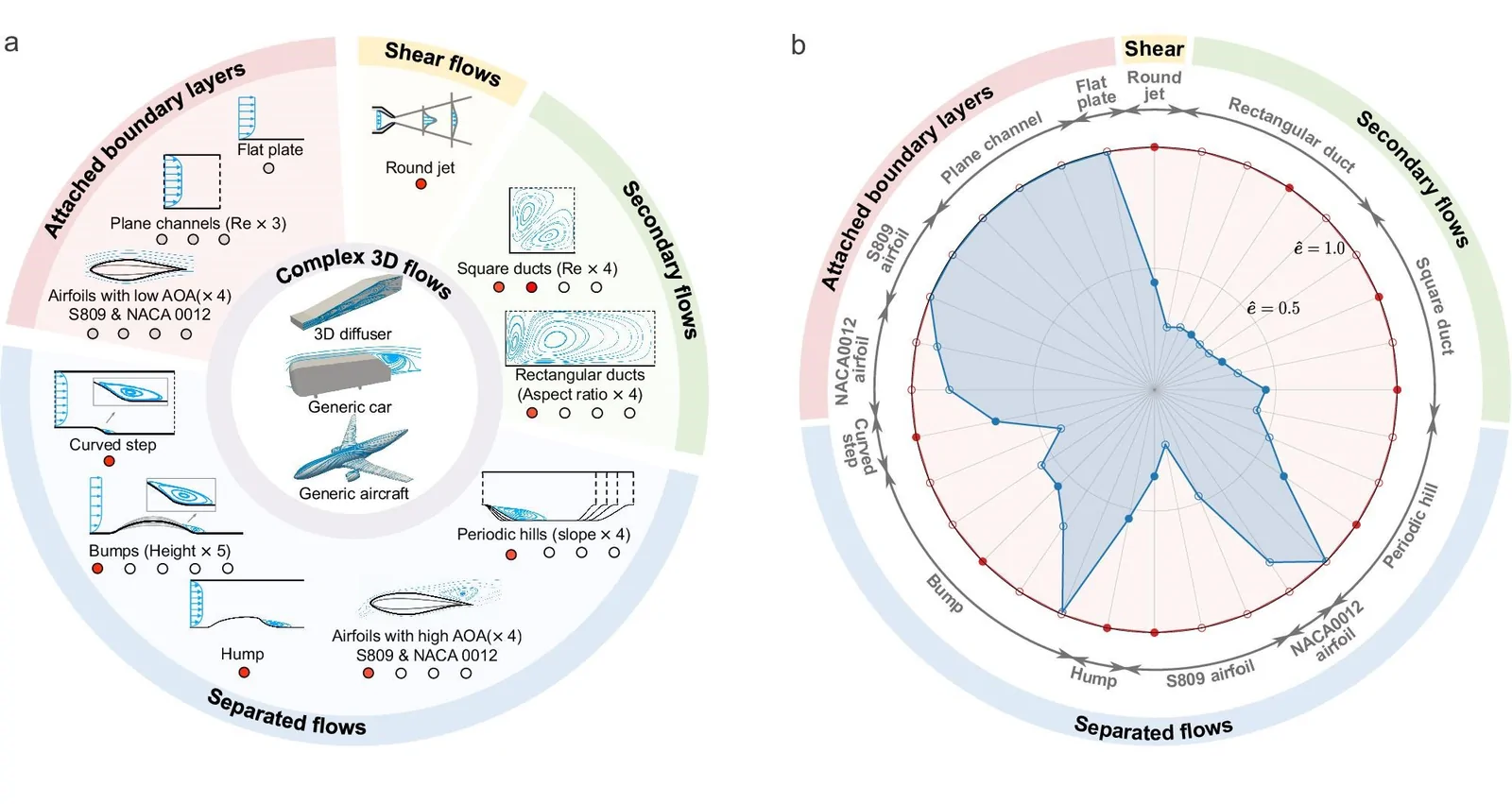
Overview of the training and evaluation cases and performance of the unified foundation turbulence model. (a) Library of canonical and complex flows used for training and evaluation, comprising 36 cases in total. Canonical flows are grouped into four categories—attached boundary layers, free-shear flows, secondary flows, and separated flows—while three complex three-dimensional flows involving multiple mechanisms are shown in the inner circle. Nine representative cases (highlighted with filled circles) form the training set, and the remaining 27 cases are used for testing. (b) Radar chart comparing normalized misfits of the baseline and unified foundation models across flow categories. Smaller radial distances indicate improved performance. The unified foundation model demonstrates robust performance across all categories while maintaining accuracy comparable to the baseline for attached boundary layers.

Developing a unified turbulence model requires training data that span diverse flow mechanisms while keeping the training cost manageable. To this end, we compiled a comprehensive library of benchmark flows from the literature, including both classical datasets [27] and recent datasets designed for data-driven turbulence modeling [28]. The resulting library comprises 36 flow cases spanning canonical to complex configurations; nine representative cases are selected for training using a hierarchical clustering strategy based on probability-distribution distances (described below), while the remaining cases are reserved for testing (see Fig. 2a). As extrapolation beyond the training feature space remains challenging for data-driven turbulence models [29,30], the evaluation focuses on generalization within the feature space spanned by the training flows. For each flow, appropriate quantities of interest (for example, velocity profiles, drag, and lift; see Table 1) are identified based on the underlying physics and modeling challenges, providing a rigorous basis for model evaluation. Unlike data-rich fields such as weather forecasting or genomics, turbulence modeling encompasses many distinct flow mechanisms but offers limited accessible data, making principled case selection essential and requiring an objective notion of similarity between flows. To define such distances systematically, we refrain from heuristic judgment, which lacks objectivity and reproducibility, and from pointwise flow comparisons, which are sensitive to mesh resolution. Instead, we assess flow similarity by comparing probability distributions of local, frame-invariant flow features (see Fig. 1b). Together, this dataset compilation and distribution-based selection strategy yields a training set that spans dominant flow mechanisms while preserving physical representativeness.

**Table 1.** Categorized benchmark turbulent flows for evaluating the unified foundation model.

Categories	Flows	Control parameter	Observations	No. Train	No. Test
Attached boundary layers	Flat plate		Velocities, friction		1
	Plane channel	Reynolds number	Velocities		3
	Airfoil (attached)	Shape, angle of attack	Lift, drag		4
Separated flows	Curved step		Velocities	1	
	Hump		Velocities	1	
	Bump	Height	Friction	1	4
	Periodic hill	Slope steepness	Velocities	1	3
	Airfoil (separated)	Shape, angle of attack	Lift, drag	1	3
Secondary flows	Square duct	Reynolds number	Velocities	2	2
	Rectangular duct	Aspect ratio	Velocities	1	3
Free-shear flows	Round jet		Velocities	1	
Complex 3D flows	Generic car		Drag		1
	3D diffuser		Velocities, friction		1
	Generic aircraft	Angle of attack	Lift, drag, friction, pressure		2
**Totals**				**9**	**27**

The benchmark covers five categories: attached boundary layers, separated flows, secondary flows, free-shear flows, and complex three-dimensional flows. Most cases vary by control parameters such as Reynolds number, angle of attack, or geometric features (for example, bump height, hill slope steepness), while cases without such parameters correspond to a single configuration. Available observations include velocity, wall friction, lift, and drag. ‘No. Train’ and ‘No. Test’ denote the numbers of training and test cases, respectively. The datasets include flat plate [39], plane channel [27], S809 and NACA 0012 airfoils [40,41], curved step [42], bump [43], hump [44], periodic hill [28], square duct [45], rectangular duct [46], round jet [47], generic car [48], three-dimensional diffuser [49], and generic aircraft [50].

#### Multi-objective ensemble learning from sparse, indirect observations

To learn a unified turbulence model from sparse, indirect observations across multiple flows, we develop a learning framework that combines ensemble-based inference with multi-objective optimization (see Fig. 1c). In practice, most experimental and engineering datasets provide only indirect measurements, such as velocity profiles or integral forces, rather than full-field turbulence quantities [24,31]. Learning from such data using gradient-based methods typically requires adjoint-enabled or fully differentiable solvers [32–34], which are often unavailable in complex engineering applications. We therefore employ a regularized ensemble Kalman learning framework [35], which enables non-intrusive parameter updates through ensemble-based covariances between model predictions and observations while constraining deviations from a physically meaningful baseline model [36]. Beyond learning from indirect data, unified turbulence modeling requires balancing performance across multiple flows and quantities of interest. Rather than optimizing a single aggregated loss, we formulate training as a multi-objective optimization problem and seek Pareto-optimal solutions that reconcile competing objectives across flow regimes [37,38]. Although originally developed for gradient-based learning, this framework naturally extends to ensemble-based updates by interpreting objective-specific ensemble corrections as generalized descent directions. Together, this ensemble-based, multi-objective formulation enables robust learning of a single turbulence model across diverse flows and observational constraints. Sensitivity analyses of the training set selection and model size further confirm this robustness (Section S3).

When application-specific accuracy is prioritized, the unified foundation model can be further adapted into a specialist model through additive fine-tuning, in which a compact correction module is trained while all foundation parameters remain fixed. This minimal-modification strategy reallocates model capacity toward targeted flow mechanisms while preserving the generalization and robustness inherited from the unified foundation model.

In summary, the proposed framework integrates a flexible and physically consistent model representation with representative training-case selection and multi-objective ensemble learning. The framework targets generalization within the feature space spanned by the training flows, while regularization toward the baseline model ensures stable behavior outside this regime. As demonstrated in the Results section, this integrated design enables robust and generalizable predictions across diverse flow phenomena without the need for manual zoning or handcrafted blending. Together, these elements advance turbulence modeling toward solutions that are both predictive and practical for large-scale engineering applications. More importantly, the methodology itself is scalable to a large number of flows and target quantities of interest—up to 40 objectives [37]—offering a viable path toward unifying diverse benchmark flow mechanisms and enabling application to truly complex industrial configurations.

## RESULTS

### Unified foundation model

We developed a unified turbulence modeling framework that is trained once and generalizes well across a broad range of flows, including attached boundary layers, separated, secondary, and free-shear flows. Based on this framework, we construct a unified foundation model that integrates multiple diverse flow mechanisms into a single formulation. Unlike most existing data-driven models, which are trained for narrow flow categories and tend to degrade outside their training regimes [13], our model performs well on the training flows and improves predictions for unseen flows exhibiting similar flow mechanisms. This directly addresses long-standing challenges emphasized in recent reviews and community discussions [11–13], particularly the need for general turbulence models that deliver consistent, broadly applicable improvements while remaining robust and free from manual zoning. Such requirements were central to the NASA 2022 Collaborative Testing Challenge [11]. Our unified foundation model makes substantial progress toward this goal, delivering robust and accurate predictions across multiple flow regimes. The NASA challenge cases, included in our evaluation, also demonstrate clear performance improvements (Section S5).

#### Model training and evaluation setup

The unified foundation turbulence model is trained on nine flows representing distinct flow mechanisms, identified using the distribution-based training set selection method. These include a curved step and a periodic hill for internal separated flows; a bump, a hump, and an S809 airfoil at high angle of attack for external separated flows; two square ducts at different Reynolds numbers and a rectangular duct for secondary flows; and a round jet for free-shear flows. Each case provides sparse, indirect, and heterogeneous observations tailored to its nature: sparse velocity measurements for the curved step, periodic hill, bump, hump, square and rectangular ducts, and round jet; and aerodynamic forces for the S809 airfoil. In total, 10 training objectives are defined, as the S809 airfoil case involves two competing objectives: lift and drag. Once trained, the model’s generalization is tested on 27 unseen cases without manual zoning or parameter tuning, as shown in Fig. 2a and Table 1 (Section S4).

#### Evaluation metric

The unified foundation model performs well across all training cases and generalizes effectively to unseen cases, including both those within the training flow categories and complex three-dimensional configurations. Its performance is evaluated on diverse test cases spanning attached boundary layers, separated flows, secondary flows, free-shear flows, and complex three-dimensional flows. The model’s performance across training flow categories is shown in Fig. 2b. It is evaluated using the normalized misfit $\hat{e} = \frac{e_{\mathrm{unified}} - e_{\mathrm{single}}}{e_{\mathrm{base}} - e_{\mathrm{single}}}$, where $e_{\mathrm{unified}}$ is the misfit of the unified foundation model relative to the ground truth, $e_{\mathrm{base}}$ is the misfit of the baseline model, that is, Wilcox (1988) *k*–*ω* model [51], and $e_{\mathrm{single}}$ is the misfit of a single-case trained model, which defines the upper performance limit. A value of $\hat{e} < 1$ indicates improved performance over baseline, while $\hat{e} > 1$ indicates degradation.

#### Performance evaluation of the unified foundation model

**Figure 3.**
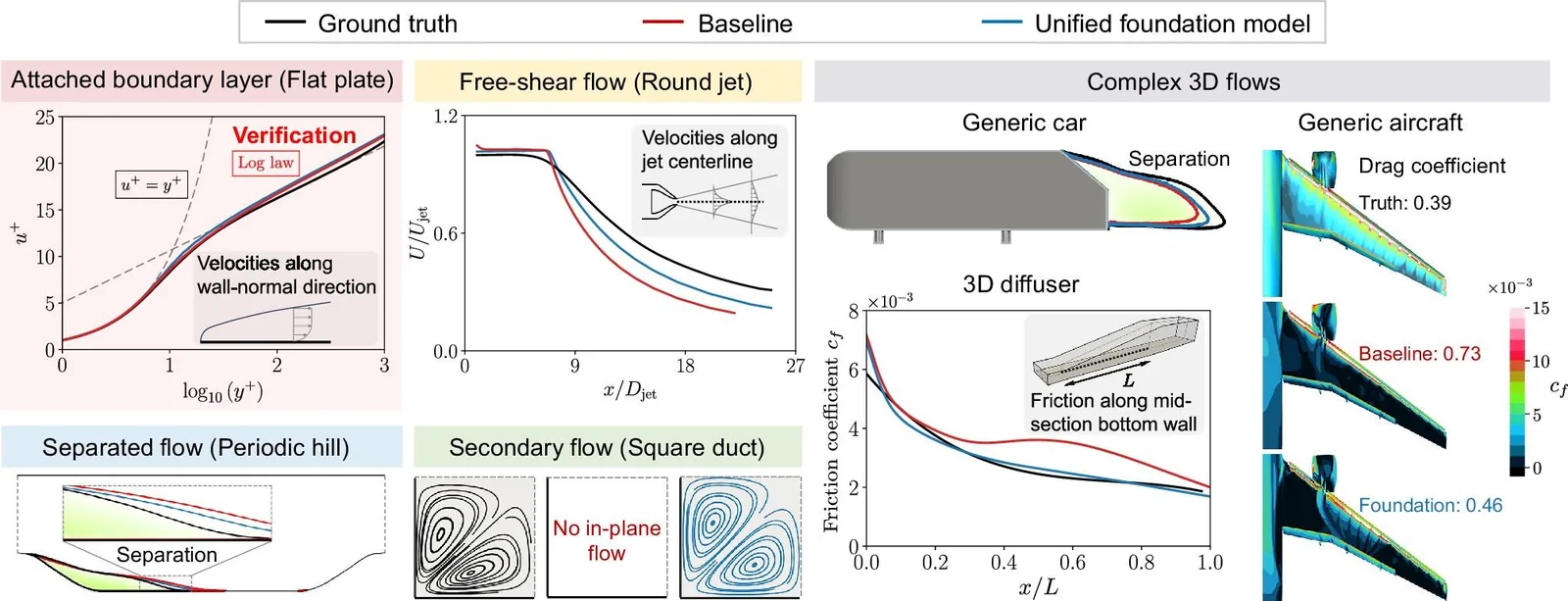
Performance evaluation of the unified foundation model on representative canonical flows and complex three-dimensional flows, comparing the ground truth, baseline model, and unified foundation model. The flat plate represents the attached boundary layer, with the inner-scaled velocity $u^+$ vs. the wall distance $y^+$ and the logarithmic law shown for reference. The round jet represents the free-shear flow, with the normalized streamwise velocity along the jet centerline. The periodic hill represents the separated flow, where the shaded regions from the ground-truth streamwise velocity field indicates regions with *u< 0*, darker shading corresponds to stronger backflow, and contour lines mark *u=0*. The square duct represents the secondary flow, with cross-sectional in-plane velocity streamlines illustrating the secondary motion. The complex three-dimensional cases include the generic car, where the separated wake region is visualized using the same ground-truth *u< 0* shading and *u=0* contours; the three-dimensional diffuser, with the skin-friction coefficient $c_f$ along the bottom wall midsection; and the generic aircraft, with surface distributions of $c_f$ and the drag coefficient.

Overall, the unified foundation model generalizes well, achieving lower misfits than the baseline in most cases and comparable performance otherwise. For attached boundary layers, such as the zero pressure gradient flat plate, the model matches the baseline model in regimes where linear eddy-viscosity models are already reliable, recovering the law of the wall and maintaining accuracy across geometries and Reynolds numbers. In free-shear flows, represented here by a round jet, the model provides more accurate predictions of centerline velocity decay, a key metric that reflects the jet spreading rate. Since this behavior is strongly influenced by the turbulent transport equations, the improved prediction highlights the importance of maintaining physical consistency between the constitutive relation and the transport equations. For secondary flows in ducts with different Reynolds numbers and aspect ratios, the model better captures in-plane flows, which require a nonlinear eddy-viscosity model to represent the anisotropic stress driving the secondary motion, and maintains accuracy under both geometric and Reynolds number extrapolation. For separated flows, the model substantially improves predictions of massive separation, such as periodic hills with varying slope steepness, bumps with different heights, the hump, the curved step, and S809 airfoil at high angles of attack. These improvements mainly arise from the spatial variation of the leading tensor-basis coefficient $g^{(1)}$ shown in Fig. 1a, which represents the eddy-viscosity contribution and directly governs separation and reattachment. For complex three-dimensional flows, including the generic car, three-dimensional diffuser, and generic aircraft, the model generally improves predictions across different flow mechanisms, while remaining comparable to the baseline otherwise. The generic car shows a modest improvement in separation prediction over the baseline; the three-dimensional diffuser recovers the ground-truth skin-friction coefficients along the bottom-wall midsection, where secondary flow interacts with separation; the generic aircraft exhibits improved skin-friction and drag predictions under large angles of attack, where multiple interacting flow mechanisms are present with separation being the dominating effect. The overall improvements for complex three-dimensional flows remain limited, with the diffuser separation location showing some improvement but still exhibiting noticeable discrepancies. Detailed performance of the unified foundation model on seven representative test cases is shown in Fig. 3. The unified foundation model demonstrates improved generalization across diverse flow regimes, achieving better, or at least comparable, accuracy to the baseline model in all test cases.

### Specialist model

The specialist model further improves prediction accuracy for targeted flows through additive fine-tuning of the unified foundation model on three representative cases with related flow mechanisms. Building on the unified foundation model, it preserves much of the foundation performance while reallocating model capacity toward the target mechanisms. Although some generalization capability may be reduced, this trade-off yields substantially improved accuracy for the targeted flows. Such specialization reflects common industrial practice, where high fidelity for specific applications is prioritized over uniform performance.

#### Model training and evaluation setup

The specialist model targets flows dominated by separation and secondary flows. Fine-tuning is performed using three canonical cases: the curved step representing separated flows, and rectangular ducts with aspect ratios of 3 and 10 representing secondary flows. These cases are selected as the three closest matches to the target flows using the distribution-based training case selection method. This combination enables the model to improve its performance for both secondary and separated flow mechanisms. The specialist model is evaluated on all benchmark canonical flows and further tested on an asymmetric three-dimensional diffuser flow, which involves interactions between the fine-tuned flow mechanisms. The three-dimensional diffuser is a challenging validation case featuring incompressible, asymmetric internal flow with strong adverse pressure gradients and significant Reynolds-stress anisotropy. The resulting three-dimensional separation closely mirrors practical diffuser behavior, making it an ideal benchmark for evaluating separation and secondary flow prediction capabilities.

#### Performance evaluation of the specialist model

**Figure 4.**
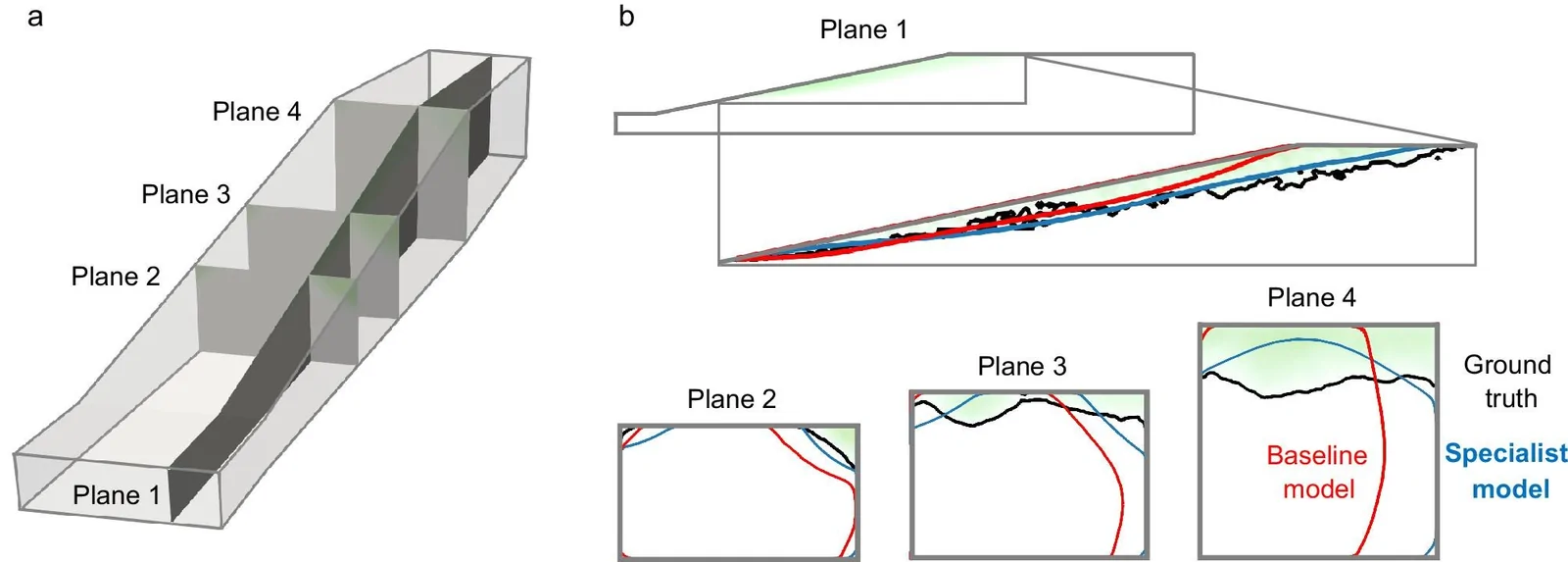
Significant performance improvement of the specialist turbulence model for a complex three-dimensional diffuser flow. (a) The diffuser geometry and sampling planes are shown for evaluation, with *x* and *z* denoting the streamwise and spanwise directions, respectively. Plane 1 is a spanwise cross-section at *z/B=0.75*, where *B* is the diffuser inlet width. Planes 2–4 are streamwise cross-sections extracted at $x/h_{\mathrm{d}}=5$, 8, and 15, where $h_{\mathrm{d}}$ is the diffuser inlet height. The ground truth on Plane 1 is obtained from experiments [49], whereas the ground truth on Planes 2–4 is obtained from the DNS mean flow field [52]. (b) The separation predictions from the ground truth, baseline model, and specialist model are compared on these planes. Flow separation is identified by negative streamwise velocity *u< 0*: shading denotes flow separation in the ground truth, with darker shading indicating stronger reverse flow, while the *u=0* contours enable clear comparison of the separation predictions.

The specialist model maintains good generalization across all benchmark canonical flows, while delivering clear performance enhancements for the fine-tuning flow categories and, in particular, for the complex three-dimensional diffuser case. With additive fine-tuning, the model reduces the over-prediction of separation in the curved step and accurately recovers in-plane secondary flows in rectangular ducts. While the unified foundation model improves predictions for both flows, fine-tuning delivers substantially higher accuracy. Moreover, the specialist model captures diffuser separation more accurately, correcting the baseline *k*–*ω* model’s sidewall separation and aligning it with the ground truth, where separation occurs along the upper wall in the expansion region. The evaluation results are summarized as follows:

(i) *Generalization in benchmark flows*. The specialist model remains robust across all benchmark canonical flow cases. Within the fine-tuning flow categories, including secondary and separated flows, most cases show clear performance improvements relative to the unified foundation model. Outside these categories, predictive accuracy is mildly degraded and remains comparable to, or slightly below, the baseline model. Nevertheless, the specialist model continues to satisfy essential physical constraints, including consistency with the logarithmic law of the wall (Section S5).

(ii) *Generalization to complex 3D flow*. In the three-dimensional diffuser, where secondary flows interact with separation, the specialist model suppresses spurious sidewall separation and correctly predicts the primary upper-wall separation observed in the ground truth, as shown in Fig. 4. Cross-sectional analyses show that the specialist model accurately captures the growth and reattachment of the separated region, while the baseline model misplaces both the location and extent of separation (Section S5). The specialist model’s success on this complex configuration demonstrates strong generalization beyond the training manifold and highlights its ability to capture interacting flow mechanisms in industrial applications.

### Interpretation for model unification

The unified turbulence model combines two complementary strategies for flow mechanism unification: automatic balancing of overlapping input features and internal branching for case-specific features across multiple flows. In overlapping regions, where identical features occur in different flows, the model resolves conflicts through multi-objective learning. In case-specific regions, it learns distinct mappings for each flow, acting as a piecewise function with internal branching. Together, these mechanisms enable the model to generalize across flows governed by distinct yet interacting flow mechanisms. We next use the unified foundation model to interpret how these strategies enable generalization.

**Figure 5.**
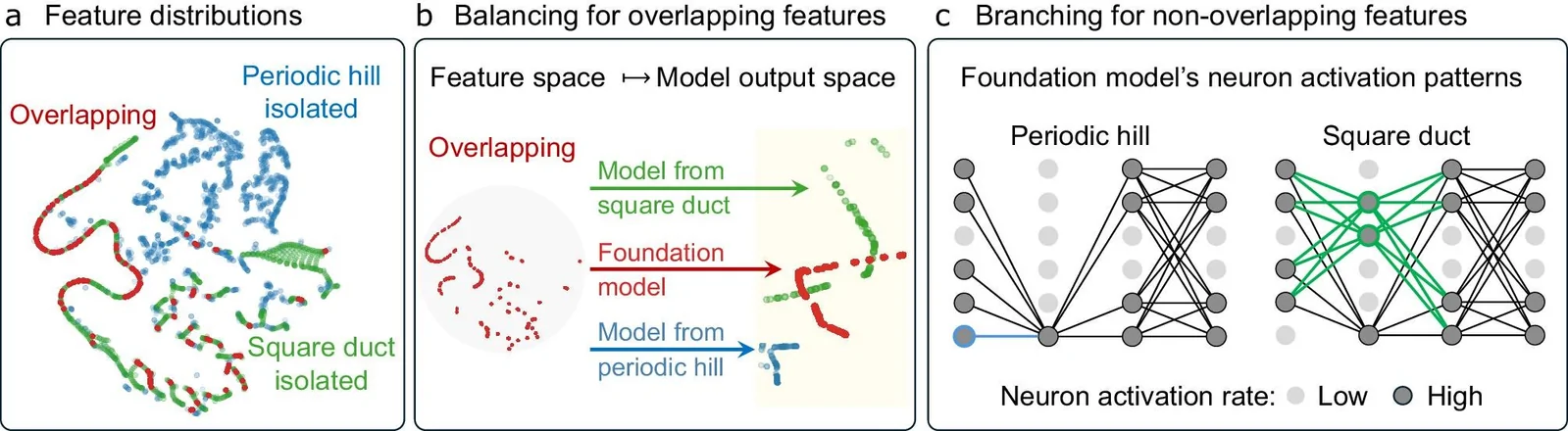
Feature-space overlap and conflict resolution enabled by the unified foundation model. (a) The t-SNE projection reduces the four-dimensional input feature space to two dimensions for visualization and shows the feature distributions of the periodic hill and square duct, including case-specific regions as well as overlapping regions shared by both flows. (b) For overlapping features, the predicted coefficient functions $g^{(1)}$ and $g^{(2)}$ are compared in the shared feature regions for the single-case trained models and the unified foundation model, showing how the unified foundation model balances conflicting model responses. (c) For non-overlapping features, neuron activation patterns in the sub-network associated with the quadratic coefficient $g^{(2)}$ are shown separately for periodic hill and square duct features, indicating case-dependent branching within the unified foundation model.

The unified foundation model’s input feature analysis reveals that different flows exhibit overlapping features while also maintaining distinct case-specific structures. We project the four frame-invariant input features into two dimensions using t-SNE for visualization [53]. The feature distributions of two representative flows, periodic hill and square duct, are shown in Fig. 5a. The distributions contain both overlapping and case-specific regions, indicating that the unified foundation model aims to capture shared structures across flows while preserving case-specific characteristics.

#### Balancing in overlapping regions

Overlapping regions in the feature space serve as critical zones where the model must resolve conflicts among cases to enable joint learning of interacting mechanisms. Because identical features in these regions can correspond to different outputs across cases, deterministic models alone cannot eliminate the resulting discrepancies. Multi-objective learning addresses this by adjusting the contribution of each case during training, promoting balanced optimization and compelling the model to reconcile conflicting objectives. As shown in Fig. 5b, the single-case trained models often map overlapping features to significantly different outputs, creating distinct regions in the output space and limiting generalization. The unified foundation model resolves these conflicts by maintaining a balanced representation, achieving an intermediate and more generalizable state. This balancing not only enhances generalization but also preserves the ability to capture genuine interactions where multiple mechanisms coexist.

#### Origins of feature space overlapping

Conflicts can originate not only from different flow mechanisms across different flows, but also from conflicting quantities of interest within the same flow. Quantities of interest from the same flow are not necessarily aligned: while some probe the same dominant mechanism and respond consistently to model corrections, others reflect different physical sensitivities. When training relies on only a subset of these quantities, the resulting model can neglect unresolved mechanisms, leading to degraded predictions for the others and the appearance of conflicting objectives. For example, in the curved-step flow, the velocity field and skin-friction coefficient are aligned, both governed by separation and reattachment dynamics. In contrast, for the S809 airfoil, lift and drag impose conflicting corrections on the dominant closure coefficient. This conflict is reflected not only at the level of integrated quantities but also in the spatial structure of the learning signals: the gradients with respect to the turbulent eddy viscosity induced by lift and drag exhibit opposing patterns across the flow domain (see Fig. S10), indicating that these two objectives drive the model toward fundamentally different flow adjustments. These competing directions correspond to distinct physical pathways, with lift favoring enhanced separation and drag favoring increased wake mixing (Section S3.2). Together, these observations provide a concrete physical basis for the conflicts observed in overlapping feature regions and motivate the need for multi-objective learning to reconcile them within a unified framework.

#### Branching in case-specific regions

In non-overlapping regions of the feature space, the network learns case-specific mappings, and effectively behaves like a piecewise function. To study this behavior, we extract case-specific features and pass them through the unified foundation model. For each neuron, we compute its activation rate, defined as the fraction of inputs producing a positive output. For rectified linear units (ReLU), this is equivalent to the proportion of non-zero outputs. The analysis is performed separately for the periodic hill and square duct. The activation patterns of the sub-network for the quadratic coefficient $g^{(2)}$ are shown in Fig. 5c. Dark neurons and connections represent pathways activated in both the periodic hill and square duct cases, highlighting their similarities. In the periodic hill, most first-layer neurons are active, with one additional neuron uniquely activated (blue), but the second layer channels information almost entirely through a single pathway. In contrast, in the square duct, second-layer activations are more widely distributed, with several unique pathways remaining active (green). These distinct connection patterns demonstrate how the model reconfigures its internal branching to adapt to the statistical and structural characteristics of each flow.

## DISCUSSION AND CONCLUSIONS

An accurate, predictive turbulence model that performs reliably across a wide range of flow mechanisms, often simultaneously present in industrial flows, has long been a central goal in industrial computational fluid dynamics. In this work, we demonstrate that multi-objective learning combined with a physically consistent model representation and distribution-based training set selection enables the training of a unified foundation model on a heterogeneous dataset consisting of nine canonical flows (for example, periodic hill for separated flows, rectangular duct for secondary flows, and round jet for free-shear flows) and various target quantities (for example, velocities, drag, and lift). With the strategy of “unification through balancing and branching”, this unified foundation model achieves Pareto-optimal performance across a broad range of test cases, including previously unseen and complex scenarios involving multiple interacting flow mechanisms. Learning from canonical flows provides interpretability, ease of verification, and a scalable route to generalization. In contrast to training directly on application-specific flows, which are often inaccessible or lack clear mechanism labeling, our approach provides a structured path to unification by expanding coverage with canonical flows through multi-objective learning, balancing trade-offs in parallel while avoiding catastrophic forgetting [20].

While the unified foundation model yields consistent improvements over the baseline model, not all test cases show dramatic gains. This is expected, as many industrial flows involve additional complexities beyond the training flows and limited objective set. Nevertheless, the framework proves highly effective in application-specific scenarios where a small number of flow mechanisms dominate, as additive fine-tuning reallocates model capacity toward these mechanisms and delivers substantially improved accuracy. We demonstrated this through two specialist models: one for three-dimensional diffuser flow, where adverse pressure-gradient-induced separation interacts with secondary motions, and the other for external vehicle aerodynamics, trained on flows with separations of varying severity (Section S5). These examples show that selecting representative canonical flows with related mechanisms and applying fine-tuning enables efficient training of specialist models that achieve high accuracy for the target mechanisms while maintaining strong generalization within their respective domains.

The long-term goal is to develop a unified turbulence model that performs robustly across an entire device, rather than in isolated regions. For instance, while a three-dimensional diffuser represents a single component in a gas turbine, a full engine comprises many more interacting components with distinct flow characteristics. Switching among specialist models within a single simulation would be cumbersome and potentially unstable. Ideally, a single model should adapt seamlessly across the system. Achieving this will require scaling the current framework to accommodate a significantly larger set of objectives. Recent advances in multi-objective learning suggest that neural networks can balance up to 40 objectives [37], making full-device unification technically feasible for configurations such as entire gas turbines or aircraft frames.

To achieve more consistent improvements across diverse flows and quantities, several technical enhancements are necessary. First, gradient accuracy for each objective must be improved. While our ensemble-based method is robust and broadly applicable, adjoint-based or fully differentiable solvers [54] could provide more precise gradients, enabling more sophisticated architectures. Additionally, feature augmentation could better utilize the network’s representational capacity by increasing the input space dimensionality, helping reduce objective conflicts through internal branching. However, the averaging process that maps turbulent fluctuations to mean fields inherently causes information loss, meaning some mechanisms may remain indistinguishable in the input space. In such cases, effective balancing, supported by accurate gradients, remains essential. Furthermore, this framework can be extended to unsteady flows by incorporating time-resolved observations over a finite time window [55], enabling more accurate modeling of inherently unsteady phenomena such as airfoil dynamic stall [56]. The presented framework, with its physics-informed architecture and gradient-aware training, provides a promising foundation for further advances in unified turbulence modeling.

## MATERIALS AND METHODS

### Physically consistent model representation

For constant-density, incompressible turbulent flows, the mean flow satisfies the Reynolds-averaged Navier–Stokes equations:


(1) \begin{eqnarray*}
\nabla \cdot \boldsymbol {U} &=& 0,\\
\frac{\mathrm{D}\boldsymbol {U}}{\mathrm{D}t} - \nu \nabla ^2 \boldsymbol {U} + \frac{1}{\rho }\nabla P &=& \nabla \cdot \boldsymbol {\tau },
\end{eqnarray*}

with the Reynolds stress $\boldsymbol {\tau } = -2k\!\left(\mathbf {b} + \frac{1}{3}\mathbf {I}\right)$. The transport of turbulence scales (the turbulent kinetic energy *k* and the specific dissipation rate *ω*) is described by partial differential equations (PDE):


(2) \begin{eqnarray*}
\boldsymbol {\mathcal {R}}\left[k,\omega ; \, {\boldsymbol{\beta} }(\boldsymbol {q})\right] = \mathbf {0} ,
\end{eqnarray*}

where ${\boldsymbol{\beta}}$ consists of the constant coefficients in the transport PDE, while the Reynolds stress anisotropy $\mathbf {b}$ is modeled using the general eddy-viscosity constitutive relation [22,57]:


(3) \begin{eqnarray*}
\mathbf {b} = \sum _{i=1}^{10} g^{(i)}(\boldsymbol {q})\, \mathbf {T}^{(i)} .
\end{eqnarray*}

The transport equations and constitutive relation are modified simultaneously by formulating the coefficients ${\boldsymbol{\beta}}$ and $g^{(i)}$ in both relations as function of local features $\boldsymbol {q}$ using parallel tensor basis neural networks with essential physical constraints [23,25]. More details can be found in the Section S2.1.

### Distribution-based training set selection

Each flow case is represented by the empirical distribution of its local, frame-invariant flow features. At each grid point, an invariant feature vector $\boldsymbol {q}(x)\in \mathbb {R}^4$ is extracted, and the collection of these samples induces an empirical probability measure in feature space. The training case selection procedure proceeds as follows:

- *Computing pairwise flow similarity.* Similarity between two flow cases with feature distributions $\pi _1$ and $\pi _2$ is quantified using the Wasserstein-2 distance. The squared Wasserstein-2 distance is defined as
  (4) \begin{eqnarray*}
W_2^2 = \min _{\gamma \in \Gamma (\pi _1,\pi _2)} \int \Vert \boldsymbol {q}-\boldsymbol {q}^{\star }\Vert ^2 \, \mathrm{d}\gamma (\boldsymbol {q},\boldsymbol {q}^{\star }),
\end{eqnarray*}
  where $\boldsymbol {q}$ and $\boldsymbol {q}^{\star }$ denote features sampled from these two flow cases, and $\Gamma (\pi _1,\pi _2)$ denotes the set of admissible transport plans.
- *Clustering and embedding.* Pairwise Wasserstein distances between all candidate flow cases are assembled into a symmetric distance matrix. Hierarchical clustering is applied to identify groups of statistically similar flows without prescribing the number of clusters *a priori*. To aid interpretation of the clustering structure, multi-dimensional scaling is used to embed the flow cases into a 2D plane that approximately preserves the pairwise distribution distances.
- *Selecting representative training cases.* Representative training cases are selected as cluster medoids, defined as the cases that minimize the sum of distances to all other members within the same cluster.

This procedure ensures coverage of dominant flow mechanisms while avoiding redundant cases and limiting the overall training cost. Details are represented in Section S2.2.

### Multi-objective ensemble learning

We couple regularized ensemble learning from indirect data with a multi-objective strategy to balance competing training cases and quantities of interest. Given *N* objectives, each defined by a training case and a quantity of interest, the update at each iteration proceeds as follows:

- *Computing regularized EnKF updates for each objective.* For objective *j*, sparse observations $\mathbf {y}_j$ are related to the model parameters $\mathbf {w}$ through an observation operator $\mathcal {H}_j$. An objective-specific ensemble update $\Delta \mathbf {w}_j$ is computed using the regularized ensemble Kalman filter method, which leverages ensemble covariances and avoids explicit adjoint gradients. The update is obtained by minimizing the cost function with regularization:
  (5) \begin{eqnarray*}
\ell _j &=& \big\Vert \mathbf {w}^{i+1}_j - \mathbf {w}^i_j \big\Vert _{\mathsf {P}^{-1}}^2\\
&&+\, \big\Vert \mathbf {y}_{\! j} - \mathcal {H}_j \big[\mathbf {w}^{i+1}_{\! j} \big] \big\Vert _{\mathsf {R}^{-1}}^2\\
&&+\, \big\Vert \mathcal {G}\big[\mathbf {w}^{i+1}_j \big] \big\Vert _{\mathsf {Q}^{-1}}^2,
\end{eqnarray*}
  where $\mathcal {G}$ penalizes deviations from a physically meaningful baseline model and stabilizes learning from limited, indirect data.
- *Reconciling conflicting objectives by adaptive weighting.* Multiple objective-specific updates $\lbrace \Delta \mathbf {w}_j\rbrace _{j=1}^N$ can point in competing directions. To balance them, we seek non-negative weights $\boldsymbol {\xi } = (\xi _1,\dots ,\xi _N)$, with $\sum _{j=1}^N \xi _j = 1$, such that the combined update direction $\sum _{j=1}^N \xi _j \, \Delta \mathbf {w}_j$ minimizes interference among objectives. Treating each $\Delta \mathbf {w}_j$ as a generalized gradient direction, we normalize the updates to reduce sensitivity to heterogeneous scales and determine $\boldsymbol {\xi }$ by minimizing
  (6) \begin{eqnarray*}
&&\min _{\boldsymbol {\xi }} \left\Vert \sum _{j=1}^N \xi _j \, \Delta \mathbf {w}_j \right\Vert ^2,\\
&& {\rm s.t.}\ \xi _j \ge 0, \quad \sum _{j=1}^N \xi _j = 1.
\end{eqnarray*}
  This auxiliary problem is solved using the Frank–Wolfe algorithm, which iteratively identifies the most conflicting objective and adjusts the weights via an analytic line search, yielding a compromise direction that balances progress across all objectives.
- *Forming the unified update.* The final model update blends the objective-specific EnKF updates using the learned weights:
  (7) \begin{eqnarray*}
\mathbf {w} \leftarrow \mathbf {w} + \eta \sum _{j=1}^{N} \xi _j \, \Delta \mathbf {w}_j,
\end{eqnarray*}
  where *η* is a step size. See Section S2.3 for more details.

## Supplementary Material

nwag344_Supplemental_File

## Data Availability

The source code for our model and training datasets are available at GitHub: https://github.com/ITLR-DDSim/unified-turbulence-modeling.
