# Supplementary material for: Toward a unified data-driven turbulence model through multi-objective learning
Source: Natl Sci Rev. 2026 Jun 4;13(16):nwag344. doi: 10.1093/nsr/nwag344 (PMC13499236; doi:10.1093/nsr/nwag344)
Supplement: Supplementary file 1 [file nwag344_supplemental_file.pdf]

Supplementary Material for:

Toward a unified data-driven turbulence model through multi-objective learning

Zhuoran Liu, Haochen Wang, Zhuolin Zhao, and Heng Xiao

Corresponding author: Heng Xiao, E-mail: heng.xiao@simtech.uni-stuttgart.de

The Supplementary Material follows the same storyline as the main article and includes all figures and tables presented therein, along with additional technical details and supporting results. Together with the accompanying code, data, and figures [1], they facilitate understanding of the methods and ensure reproducibility of the results.

Contents

1 Introduction **S2**
1.1 Turbulence modeling: from universality to unification S2
1.2 Toward a unified turbulence model: existing approaches and key challenges S3
1.3 Scope and contributions of this work S4

2 Methodology **S5**
2.1 Flexible and physically consistent model representation S5
2.1.1 General eddy-viscosity formulation S5
2.1.2 Parallel tensor basis neural network representation S7
2.1.3 Physical consistency requirement within the turbulence model S8
2.2 Distribution-based training set selection S9
2.3 Multi-objective ensemble learning from sparse, indirect data S12
2.3.1 Multi-objective learning for unified turbulence modeling S12
2.3.2 Regularized ensemble learning from sparse, indirect observations S13
2.4 Additive fine-tuning for specialist model S16

3 Verification and validation **S17**
3.1 Effectiveness of physical consistency S17
3.1.1 Verification of physical consistency within turbulence model S17
3.1.2 Validation of physically consistent model performance S18
3.2 Characteristics of aligned and conflicting quantities of interest S19
3.2.1 Aligned quantities of interest: velocity and friction coefficient of curved step . S19
3.2.2 Conflicting quantities of interest: lift and drag of S809 airfoil S21
3.3 Effectiveness of multi-objective learning S23
3.3.1 Conflict flows: periodic hills with different separation characteristics S23
3.3.2 Non-conflict flows: square duct and airfoil flows S24
3.3.3 Scalability evaluation for multi-objective learning S26
3.3.4 Sensitivity analysis on model size S26

4 Setup of training and test cases **S29**
4.1 Training cases for unified foundation model S31
4.2 Test cases for unified foundation model S34
4.3 Specialist turbulence model for separated flows S36
4.4 Specialist turbulence model for secondary flows with separation S37

5 Results **S39**
5.1 Unified foundation model S39
5.2 Specialist turbulence model for separated flows S50
5.3 Specialist turbulence model for secondary flows with separation S55

1 Introduction

Turbulence is ubiquitous in natural and industrial flows and remains a central challenge in classical physics due to its chaotic and multiscale nature. These characteristics govern essential transport processes, such as momentum, heat, and pollutant transport, yet make it prohibitively expensive to resolve all relevant scales in practical simulations. As a result, most predictions rely on turbulence modeling within Reynolds-averaged Navier–Stokes solvers [2]. In such approaches, the averaged effects of unresolved turbulent fluctuations on the mean flow must be represented through closure models that use simplified, typically local, flow information. Despite decades of development and their central role in simulations, existing turbulence models remain crude approximations of complex, nonlinear, and multiscale turbulent dynamics. Consequently, improving the theoretical foundations and practical performance of turbulence modeling remains both scientifically fundamental and societally important.

1.1 Turbulence modeling: from universality to unification

Early efforts in turbulence modeling were shaped by Lumley's proposal that turbulent momentum transport might admit a constitutive relation, analogous to those used in continuum mechanics, linking the mean velocity field to the Reynolds stress [3]. In that work, Lumley argued that such a relation should respect fundamental symmetry and invariance properties and remain independent of boundary conditions imposed on the Reynolds stresses, thereby providing a structured framework for their representation. Using experimental data, he further demonstrated that a local constitutive relation can be identified in highly idealized settings, such as homogeneous shear and homogeneous strain flows, where turbulence exhibits viscoelastic-like behavior. However, these successes remained confined to canonical configurations and had little influence on practical turbulence modeling. In complex industrial flows, multiple mechanisms such as attached boundary layers, flow separation, curvature effects, and secondary motions often coexist and interact within the same configuration, making strict universality unlikely under practical modeling constraints. Specifically, industrial applications strongly favor local closures, in which the Reynolds stress depends only on velocity gradients at the same spatial location. In contrast, a truly universal constitutive relation—if it exists at all—would likely be nonlocal, reflecting the inherently nonlocal nature of turbulent physics. This discrepancy has also been emphasized in recent studies of machine-learning-based closures, which highlight that single-point constitutive relations cannot, in general, capture non-local transport and non-equilibrium effects through purely local mappings from mean-flow quantities to Reynolds stresses [4]. This realization motivated a gradual shift away from the pursuit of universal closures toward the more pragmatic objective of unification, in which a single model is expected to represent multiple flow mechanisms without manual zoning, switching, or retuning. As a result, robustness, simplicity, and computational efficiency became dominant considerations, leading to the widespread

adoption of empirical eddy-viscosity models such as the Spalart–Allmaras and k–ω SST models [5]. Decades of experience with such models suggest that a simple, local universal constitutive relation is unlikely to exist [6]. Nevertheless, a *unified turbulence model* that can handle multiple coexisting mechanisms remains highly desirable for industrial applications [7].

Eddy-viscosity models provide a natural foundation for a unified turbulence model. This choice is practical because eddy-viscosity models are local, robust, and computationally efficient, making them reliable for industrial solvers and a strong basis for data-driven extensions [5]. Conventional eddy-viscosity models combine turbulence transport equations for turbulent scales with a linear constitutive relation to estimate the Reynolds stress. This framework can be generalized to nonlinear forms, offering greater flexibility than the standard Boussinesq assumption [8, 9]. The general eddy-viscosity model [10] has been realized through tensor basis neural networks [11] and symbolic formulations [12, 13]. Alternatively, Reynolds stress modeled from the eddy viscosity models can also be modified directly through eigen-perturbations [14, 15]. Other studies have instead focused on improving the turbulence transport equations, for example by introducing data-driven multiplicative correction fields [16]. Beyond eddy-viscosity models, nonlocal approaches offer an alternative. These include Reynolds stress transport models [17] and data-driven nonlocal constitutive relations [18–20], which could capture additional physical effects such as non-equilibrium effects in the turbulence anisotropy. However, nonlocal models face major challenges: they are computationally expensive, less robust, and difficult to treat solid boundaries (wall models). Machine learning has also been used to refine specific terms in Reynolds stress transport models, such as the pressure–strain correlation [21], but these models themselves remain fragile. Considering these trade-offs, the tensor basis neural network formulation of the general eddy-viscosity model provides a well-balanced compromise among accuracy, flexibility, and computational efficiency.

1.2 Toward a unified turbulence model: existing approaches and key challenges

Despite the merits of eddy-viscosity models as a foundation for unified data-driven turbulence modeling, a predictive and generalizable model cannot be achieved by modifying individual closure components in isolation. Instead, success critically depends on preserving internal consistency between the turbulence transport equations and the constitutive relation for the Reynolds stress, as well as satisfying additional physical constraints. Nevertheless, most existing data-driven eddy-viscosity models modify only one of these tightly coupled components, which fundamentally limits their ability to achieve unification.

Eddy-viscosity models consist of two tightly coupled components: turbulence transport equations define the turbulent length and time scales, determining Reynolds stress amplitude, while the constitutive relation characterizes its anisotropy. Many studies focus exclusively on refining the constitutive relation or the turbulence anisotropy using tensor basis neural networks, symbolic regression, or random forests [11, 13–15], while others introduce corrections to the transport equations [16]. Although both approaches have achieved improved performance over baseline models, treating these components independently can break the physical coupling between turbulent scale evolution and the Reynolds stress anisotropy. Recent analyses have shown that such decoupled corrections can lead to severe inconsistencies, even in simple canonical flows [22, 23]. These observations highlight the importance of preserving the internal consistency in the efforts to achieve a unified turbulence model.

Beyond internal consistency, a unified eddy-viscosity model must also respect physical constraints arising from behaviors observed in canonical flows (e.g., homogeneous isotropic decaying turbulence and homogeneous shear turbulence), near-wall dynamics (law of the wall), and free-stream limits, among other [24]. These flows have traditionally been used to calibrate turbulence models, imposing additional physical constraints among model coefficients in the transport equations. These coefficients,

in turn, rely on implicit assumptions embedded in the constitutive relation. Existing data-driven studies rarely address this full set of coupled constraints in a systematic and coherent manner; instead, different works tend to modify different subsets of model components, leading to patchwise improvements that do not generalize across flow regimes. Ignoring the dependence between transport equations and constitutive relation results in inconsistent Reynolds-stress definitions and incorrect equilibrium behavior, undermining robustness and generalizability. Resolving this physical consistency issue systematically is therefore a prerequisite for unified turbulence modeling.

Within the eddy-viscosity framework, several strategies have been explored to advance turbulence unification, with varying degrees of success. Multi-case training using symbolic model representations has demonstrated promising generalization across several wall-bounded flows, representing some of the earliest steps toward unified turbulence modeling [25]. However, the restrictive functional forms of symbolic models and their reliance on derivative-free optimization methods, such as non-dominated sorting genetic algorithm II, limit the scalability of training, particularly when balancing many competing objectives required for unification. Another line of research pursues unification through aggregation, in which expert models tailored to individual flow mechanisms are combined into a composite framework using mixture-of-experts or classifier-driven model selection [26, 27]. While this strategy enables mechanism-specific modeling, it typically requires multiple model evaluations and can struggle when strong interactions between flow mechanisms occur within the same region. A different approach employs adjoint-based field inversion and machine learning to infer multiplicative correction fields to existing turbulence models [28, 29]. These methods have proven effective for flows dominated by similar mechanisms [29], but their extension to flows with fundamentally different coexisting mechanisms (e.g., separated and secondary flows, as will be shown in the three-dimensional diffuser or the wing-body junction [30]) remains challenging, often leading to non-convergent training and limited generalizability [28]. Finally, the generalized k–ω model provides a pragmatic alternative by introducing tunable closure terms that allow systematic adjustments for different flow conditions [31]. Although attractive for practical use in commercial solvers, its performance still relies heavily on manual tuning and therefore falls short of true unification. Despite these advances, a unified turbulence model capable of seamlessly handling multiple, strongly interacting flow mechanisms within a single configuration does not yet exist [7]. Achieving such a model remains a central open challenge, as improvements targeting one flow regime often degrade performance in others. Taken together, these limitations indicate that unification cannot be achieved through isolated improvements or post hoc aggregation alone, but instead requires a learning framework that jointly enforces physical consistency, balances competing objectives, and operates directly on sparse, indirect observations, since full-field Reynolds stress data are rarely available in realistic applications.

1.3 Scope and contributions of this work

In this work, we propose a unified, data-driven turbulence modeling framework that learns a single, neural-network-based eddy-viscosity model that is robust and accurate across diverse flow regimes. The approach combines a physically consistent model representation, a distribution-based selection of representative training flows, and a multi-objective ensemble learning strategy to balance competing objectives arising from sparse, indirect observations in different flow mechanisms. The resulting unified turbulence model delivers consistent, across-the-board improvements over a wide range of canonical and complex flows, while remaining compatible with existing Reynolds-averaged Navier–Stokes solvers, thereby offering a viable pathway toward industrial deployment.

2 Methodology

The proposed framework integrates several complementary components into a coherent learning pipeline that advances data-driven turbulence modeling from formulation to deployment (Fig. S1). **First**, a flexible and physically consistent model structure is established, in which the constitutive relation and turbulence transport equations are treated in a coupled, frame-invariant manner and constrained by established physical requirements, ensuring both expressiveness and physical admissibility. **Second**, representative training cases are selected using a distribution-based strategy that compares flows through distances between their flow-feature probability distributions. This selection provides broad coverage of distinct flow mechanisms while avoiding redundant information. **Third**, building on the resulting model structure and training set, learning is formulated as a multi-objective problem and carried out using a regularized ensemble Kalman framework that infers turbulence closures from sparse and indirect observations. The ensemble-based formulation enables gradient-free inference and jointly learns multiple flow cases and quantities of interest, allowing competing objectives to be reconciled and yielding a unified foundation model with balanced performance across flow regimes. When application-specific accuracy is required, the unified foundation model can be further adapted through an **optional** additive fine-tuning procedure. In this step, compact correction modules focus model capacity on target flow mechanisms while preserving the physical structure and generalization properties learned in the unified foundation model.

2.1 Flexible and physically consistent model representation

The turbulence model is designed to be frame-invariant, flexible, and physically consistent through its model representation. Frame invariance is enforced by expressing the Reynolds stress closure in a tensor-basis form, which provides a robust foundation for data-driven modeling. To improve numerical stability and explicitly regularize model nonlinearity, linear and nonlinear tensor-basis contributions are treated separately, ensuring that dominant low-order effects are captured first while higher-order corrections are introduced in a controlled manner. Physical consistency between the constitutive relation and the turbulence transport equations is enforced as an essential component of the model. Their coefficients are therefore modeled in a coupled framework as learnable fields subject to established physical constraints, including consistency with near-wall behavior such as the logarithmic law of the wall. This coupled and constrained formulation prevents unphysical Reynolds stress predictions and further enhances numerical stability.

2.1.1 General eddy-viscosity formulation

For constant-density, incompressible turbulent flows, the mean flow satisfies the Reynolds-averaged Navier–Stokes (RANS) equations:

$$\nabla \cdot \boldsymbol{U} = 0,$$
$$\frac{\mathrm{D}\boldsymbol{U}}{\mathrm{D}t} - \nu \nabla^2 \boldsymbol{U} + \frac{1}{\rho}\nabla P = \nabla \cdot \boldsymbol{\tau},$$

where $\boldsymbol{U}$ denotes the mean velocity, P is the mean pressure normalized by density ρ, and $\boldsymbol{\tau}$ is the Reynolds stress tensor that accounts for the effect of unresolved velocity fluctuations. Since these fluctuations are not resolved in RANS equations, the Reynolds stress requires a closure model.

The Reynolds stress tensor is decomposed into deviatoric and isotropic components as

$$\boldsymbol{\tau} = -\left(\mathbf{a} + \frac{2}{3}k\mathbf{I}\right),$$

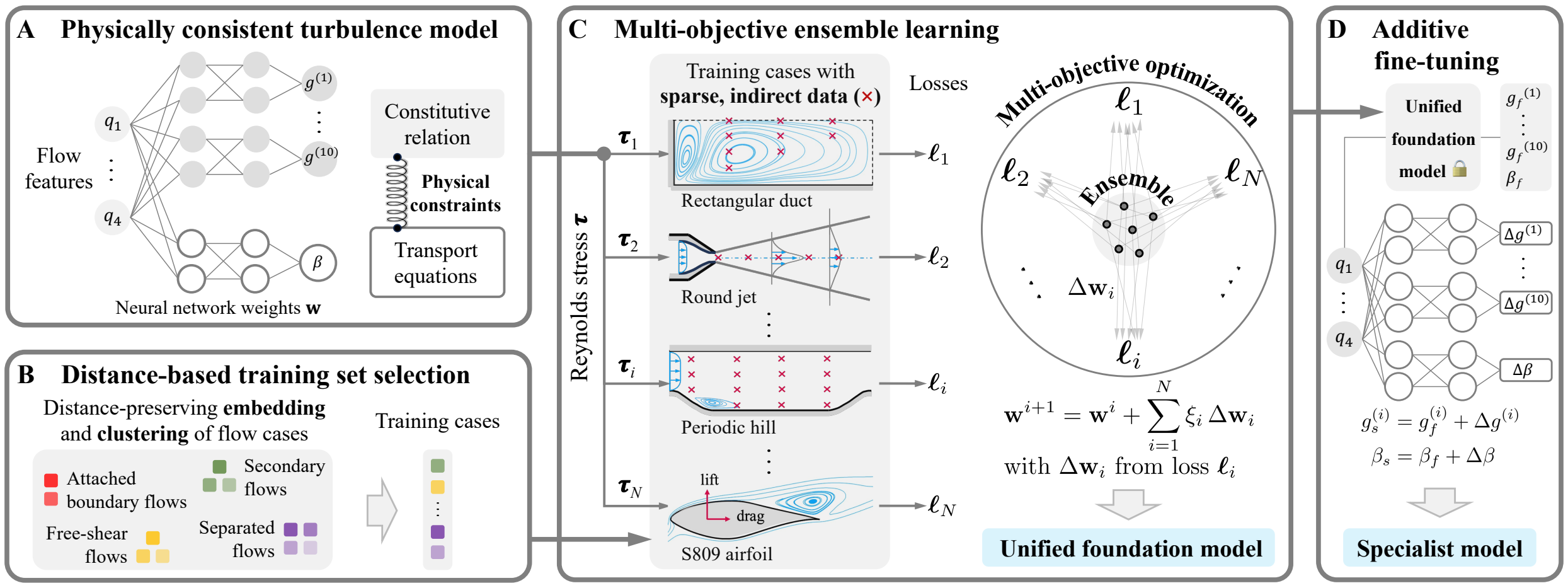

Figure S1: The proposed methodology establishes a unified foundation turbulence model that captures diverse flow regimes and supports application-specific adaptation through fine-tuning. (A) A physically consistent and frame-invariant model representation is adopted, in which a flexible turbulent constitutive relation is learned in a coupled manner with turbulence transport equations under established physical constraints, enabling consistent prediction of Reynolds stresses across different flow regimes. (B) A representative and compact training dataset is automatically identified by comparing probability distributions of local, frame-invariant flow features; distance-preserving embedding and clustering are used to select flows that span distinct flow mechanisms while avoiding redundancy. (C) The unified model is learned using an ensemble-based, multi-objective optimization framework, in which Reynolds stresses predicted by the model are propagated through the Reynolds-averaged Navier–Stokes equations and compared against sparse, indirect observations from the selected training cases, with adaptive weighting used to balance heterogeneous objectives across flows. Together, these steps yield a single set of network weights that captures multiple flow regimes within a unified foundation model. (D) For application-specific accuracy, the foundation model can be adapted into a specialist model through additive fine-tuning, in which compact correction modules target specific flow mechanisms while preserving the learned foundation behavior.

where k is the turbulence kinetic energy and $\mathbf{I}$ is the second-order identity tensor. The normalized anisotropy tensor is defined as $\mathbf{b} = \mathbf{a}/(2k)$. According to the general eddy-viscosity formulation [10], the normalized anisotropy tensor $\mathbf{b}$ can be expressed as a linear combination of ten tensor bases:

$$\mathbf{b} = \sum_{i=1}^{10} g^{(i)}(\theta_1, \ldots, \theta_5)\, \mathbf{T}^{(i)}, \tag{S1}$$

where $g^{(i)}$ are scalar coefficient functions of five invariants θ_i, and $\mathbf{T}^{(i)}$ are tensor bases. Both $\mathbf{T}^{(i)}$ and θ_i are constructed from the normalized mean strain-rate and rotation-rate tensors. The normalized strain-rate and rotation-rate tensors are commonly defined as

$$\hat{\mathbf{S}} = \tau_s \mathbf{S}, \quad \hat{\mathbf{W}} = \tau_s \mathbf{W}, \tag{S2}$$

where $\mathbf{S} = \frac{1}{2}\left[\nabla \boldsymbol{U} + (\nabla \boldsymbol{U})^\top\right]$ and $\mathbf{W} = \frac{1}{2}\left[\nabla \boldsymbol{U} - (\nabla \boldsymbol{U})^\top\right]$ are the mean strain-rate and rotation-rate tensors, respectively. The turbulence time scale τ_s is estimated as $\tau_s = k/\varepsilon = 1/(C_\mu \omega)$, where C_μ is the turbulent-viscosity constant. The first two scalar invariants and corresponding tensor bases in Eq. (S1) are defined as:

$$\theta_1 = \{\hat{\mathbf{S}}^2\}, \quad \theta_2 = \{\hat{\mathbf{W}}^2\}, \quad \mathbf{T}^{(1)} = \hat{\mathbf{S}}, \quad \mathbf{T}^{(2)} = \hat{\mathbf{S}}\hat{\mathbf{W}} - \hat{\mathbf{W}}\hat{\mathbf{S}},$$

where $\{\cdot\}$ denotes the tensor trace. Definitions of the remaining tensor bases and invariants are available in [10, 11]. For two-dimensional turbulent flows, only the first two tensor bases and invariants are required to form a complete representation.

The tensor basis neural network (TBNN) [11] provides a data-driven realization of the general eddy-viscosity model by learning the coefficient functions $g^{(i)}$ as functions of the frame-invariant scalars θ_1, ..., θ_5. These learned coefficients are then combined with the corresponding tensor basis $\mathbf{T}^{(i)}$ according to Eq. (S1) (see Fig. S2). This formulation preserves frame invariance by construction while providing a flexible neural-network representation of the constitutive relation. In classical formulations, the time-scale normalization in Eq. (S2) is applied to the strain-rate and rotation-rate tensors to render them dimensionless; however, it does not bound their magnitude and may yield excessively large values in regions of strong gradients, which is undesirable for machine learning. To ensure stable and efficient learning, the flow features derived from these tensors are required to lie within a bounded range. We therefore avoid conventional global min–max normalization, which depends on dataset extrema and may cause feature clustering near singular points. Instead, we adopt a local time-scale normalization [15, 32] applied directly to the strain-rate and rotation-rate tensors, which combines non-dimensionalization with bounded scaling and preserves local flow physics. Specifically,

$$\hat{\mathbf{S}} = \frac{\mathbf{S}}{|\mathbf{S}| + 1/\tau_s}, \quad \hat{\mathbf{W}} = \frac{\mathbf{W}}{|\mathbf{W}| + 1/\tau_s},$$

from which the scalar invariants and other flow features are subsequently derived.

2.1.2 Parallel tensor basis neural network representation

We extend the original TBNN (Fig. S2 a) to the parallel TBNN (Fig. S2 b) to enhance flexibility and control over model nonlinearity. Unlike the original TBNN, which predicts all coefficients using a single network, the parallel TBNN employs multiple independent sub-networks to predict individual coefficient functions. In this work, we construct a quadratic eddy-viscosity model based on the first two tensor bases, $\mathbf{T}^{(1)}$ and $\mathbf{T}^{(2)}$. The model input consists of four flow features $\mathbf{q} = \{q_i\}_{i=1}^{4}$. The first two are scalar invariants derived from the velocity gradient, denoted by θ_1 and θ_2. The third feature, $q_3 = \nu_t/(100\nu + \nu_t)$, represents the viscosity ratio, and the fourth feature, $q_4 = \min\left(\sqrt{k}d/(50\nu),\, 2\right)$ corresponds to the turbulent Reynolds number [33]. These features are summarized in Table S1.

Feature	Description	Formula
q_1	First scalar invariant	$q_1 = \theta_1 = \{\hat{\mathbf{S}}^2\}$
q_2	Second scalar invariant	$q_2 = \theta_2 = \{\hat{\mathbf{W}}^2\}$
q_3	Viscosity ratio	$q_3 = \frac{\nu_t}{100\nu+\nu_t}$
q_4	Turbulent Reynolds number	$q_4 = \min\left(\frac{\sqrt{k}\,d}{50\nu},\, 2\right)$

Table S1: Summary of model input features, where curly brackets $\{\cdot\}$ indicate trace of a tensor; ν and ν_t are the laminar and eddy viscosities, $\hat{\mathbf{S}}$ and $\hat{\mathbf{W}}$ the normalized strain- and rotation-rate tensors, k the turbulent kinetic energy, and d the wall distance.

Separate sub-networks are used to predict the linear coefficient $g^{(1)}$ and the quadratic coefficient $g^{(2)}$, while sharing the same invariant input features. This parallel architecture imposes a clear

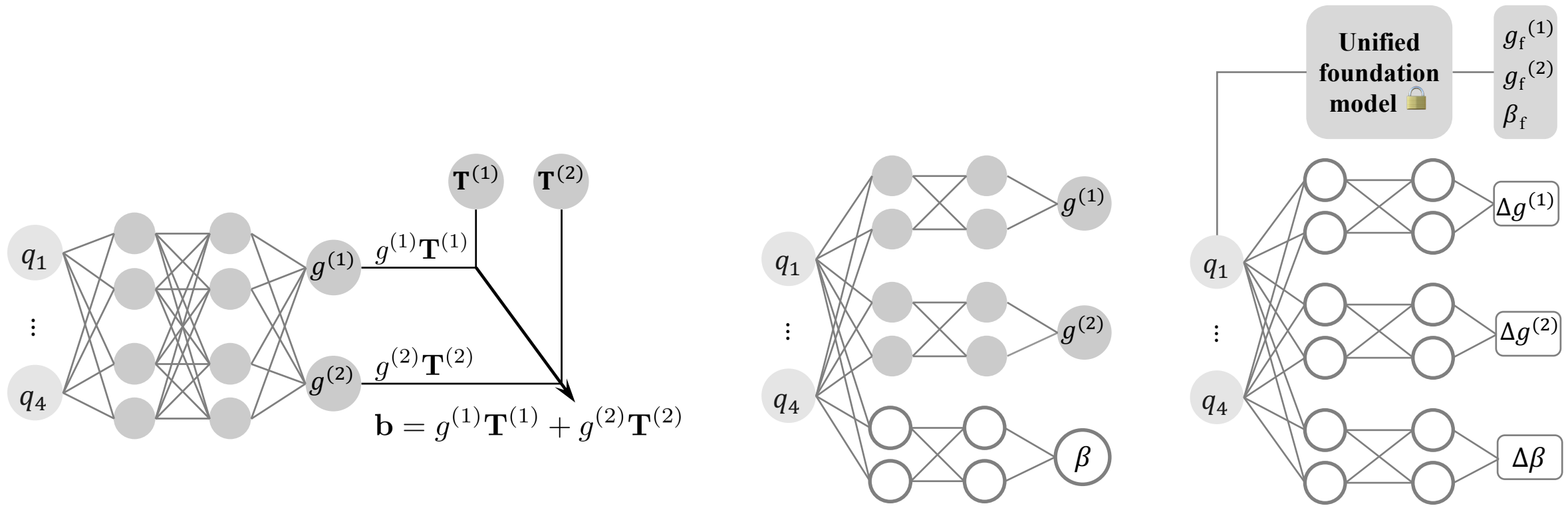

(a) Original TBNN

(b) Consistent parallel TBNN

(c) Additive fine-tuning

Figure S2: Architectural extensions of the tensor basis neural network for unified and specialist turbulence modeling. (a) Original tensor basis neural network (TBNN). A single neural network predicts the coefficient functions $g^{(i)}$ from the flow features $\boldsymbol{q}$, which are combined with the predefined tensor basis $\mathbf{T}^{(i)}$ through the constitutive relation $\sum_i g^{(i)}\mathbf{T}^{(i)}$ as in Eq. (S1). (b) Proposed consistent parallel TBNN used for the unified foundation model. Separate sub-networks predict the linear and quadratic coefficients $g^{(1)}$ and $g^{(2)}$, while an additional sub-network jointly predicts the turbulence transport-equation coefficient β, enabling enforcement of physical consistency between the constitutive relation and the transport equations. For clarity, the tensor-basis combination shown in (a) is not repeated. (c) Proposed additive fine-tuning strategy for specialist models. A compact set of additional trainable network weights is introduced to capture flow-specific corrections, while all network weights of the unified foundation model remain fixed. The underlying tensor-basis formulation is unchanged.

structural hierarchy by treating linear and higher-order nonlinear contributions independently, rather than implicitly weighting them equally within a single network. By emphasizing linear contributions and introducing nonlinear corrections in a controlled manner, the design enables physics-informed regularization, improving interpretability, numerical robustness, and generalization.

2.1.3 Physical consistency requirement within the turbulence model

To ensure physical consistency, the constitutive relation and turbulence transport equations are treated in a coupled manner and learned simultaneously under essential physical constraints. We adopt the k–ω (1988) model as the baseline,

$$\frac{\mathrm{D}k}{\mathrm{D}t} = P_k - \beta^* k\omega + \nabla\cdot\left[(\nu+\sigma^*\nu_t)\nabla k\right],$$

$$\frac{\mathrm{D}\omega}{\mathrm{D}t} = \alpha\,\frac{\omega}{k}P_k - \beta\,\omega^2 + \nabla\cdot\left[(\nu+\sigma\nu_t)\nabla\omega\right],$$

where the transport coefficients $(\alpha, \beta, \beta^*, \sigma, \sigma^*)$ are traditionally calibrated using canonical flows under the assumption of a linear eddy-viscosity model. Such fixed calibration limits generalization to complex industrial flows and becomes internally inconsistent when the constitutive relation is extended to nonlinear forms.

To address these limitations, only the dissipation coefficient β in the turbulent specific dissipation rate ω equation is treated as a spatially varying quantity and is learned jointly with the constitutive

relation. The parallel TBNN directly predicts the dissipation coefficient β together with the tensor-basis coefficients $g^{(i)}$ (Fig. S2 b). Each sub-network is implemented as a fully connected neural network: the sub-network for $g^{(1)}$ uses four hidden layers with four neurons per layer, the sub-network for $g^{(2)}$ uses four hidden layers with six neurons per layer, and the sub-network for β uses four hidden layers with four neurons per layer; all sub-networks employ the ReLU activation function, resulting in a total of 333 trainable network weights. The remaining transport coefficients are then derived sequentially using established physical constraints [34–37]. Specifically, the following constraints are enforced to ensure equilibrium behavior, near-wall asymptotics, and freestream robustness:

(1) $\beta^*/\beta = 1.25$, based on homogeneous isotropic decaying turbulence;

(2) $(Sk/\varepsilon)^2 = -\beta/(g^{(1)}\alpha\beta^*) = 17.06$, enforcing equilibrium homogeneous shear turbulence;

(3) $\sigma = \sqrt{\beta^*}\,(\beta/\beta^* - \alpha)/\kappa^2$, ensuring compatibility with the logarithmic law of the wall;

(4) $\sigma > \sigma^* > 0.5$ and $\sigma - \sigma^* \leq \sigma\sigma^*$, mitigating dependence on the prescribed freestream turbulence levels.

Here, ε denotes the turbulence dissipation rate, κ is the von Kármán constant, and $S = \sqrt{2S_{ij}S_{ij}}$ is the magnitude of the strain-rate tensor S_{ij}. Together, these conditions define the physical limits that the coupled model must satisfy in the learned model. This coupled formulation allows turbulence scales and Reynolds stress anisotropy to adapt coherently to the local flow conditions while remaining anchored to well-established turbulence physics. As a result, the proposed representation provides an expressive, frame-invariant, and physically consistent foundation for learning nonlinear eddy-viscosity models. Within this formulation, the model remains a single-point, local closure. While it does not explicitly represent non-local transport or history effects, the framework can be extended to incorporate such physics. In particular, additional transported quantities can be introduced to encode Reynolds-stress anisotropy, providing a limited form of non-locality. This extension enables improved representation of non-equilibrium behavior, while retaining the robustness and efficiency of eddy-viscosity formulations [38].

2.2 Distribution-based training set selection

Unified data-driven turbulence modeling requires training cases that span diverse flow mechanisms while avoiding redundant information and keeping training cost manageable. We address this requirement by formulating training-set selection as a distribution-based problem, in which candidate flows are compared using distances between probability distributions of physically meaningful flow features. This strategy promotes diversity in the training data and ensures that distinct flow regimes contribute complementary information to the learning of the unified model.

As a starting point, we compiled a library of 36 benchmark flow cases from the literature and used a distance-based similarity measure to quantify their distances. The compiled dataset includes both classical datasets [39] and recent datasets developed for data-driven turbulence modeling [40] (Fig. S3). Because turbulence modeling involves many distinct flow regimes but offers limited accessible data compared with data-rich fields such as weather forecasting or genomics [41, 42], principled comparison and selection of flow cases are essential. Direct pointwise comparison of flow fields is ill-suited for this purpose, as it depends strongly on mesh resolution, geometry, and grid alignment and therefore does not provide an objective basis for comparing different flows. Instead, each flow is represented statistically through the distribution of its local, frame-invariant flow features, allowing similarity to be assessed at the distribution level. Specifically, local flow features are treated as realizations of a multivariate random variable, so that each flow case induces an empirical probability distribution

in feature space. At each spatial location x, the local feature vector is denoted by $\boldsymbol{q}(x) \in \mathbb{R}^4$ (see Table S1), with each grid point providing one sample of $\boldsymbol{q}$. The resulting empirical measure μ summarizes the statistical structure of the flow and enables direct comparison between different flows independent of their geometries and mesh resolutions.

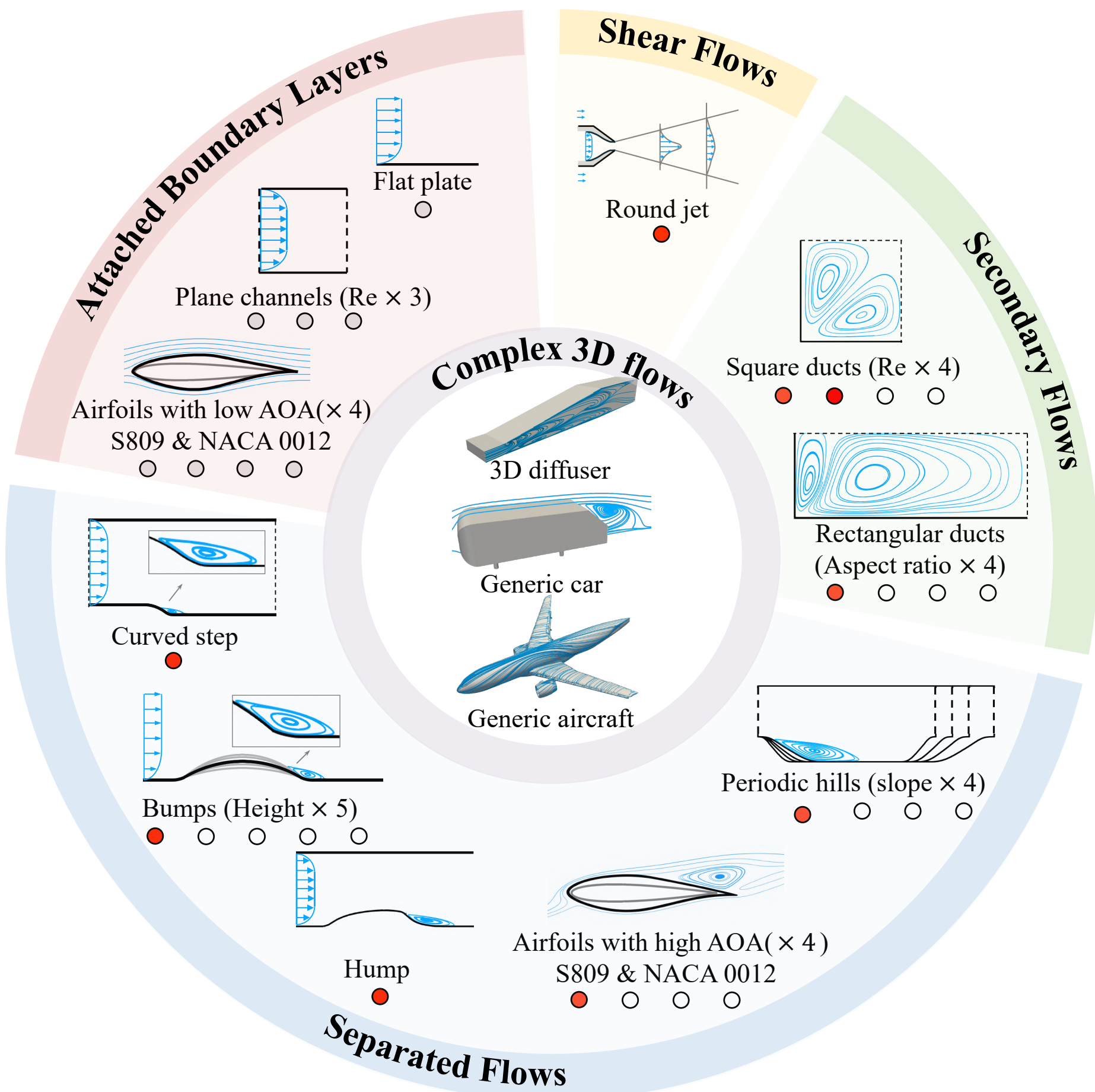

Figure S3: Canonical and complex flows used to train and evaluate the unified foundation model, comprising 36 cases in total. The canonical flows are grouped into four categories: attached boundary layers, free-shear flows, secondary flows, and separated flows, each shown as a sector of the ring, while the three complex 3D flows are placed in the inner circle. Nine representative training flows are highlighted with red circles, identified through the distribution-based training set selection, while the remaining flows are reserved for testing. The testing set includes 23 canonical flows within the training categories and three complex 3D flows. Note that the plane channels and flat plate case are reserved for validation and are thus intentionally excluded from the training set. Detailed specifications are provided in Table S5.

We employ a distance defined directly on probability distributions to quantify the similarity between two flow cases with feature distributions π_1 and π_2 (see Fig. S4). Specifically, we use the Wasserstein-2 distance from optimal transport theory, which measures how much "effort" is required to transform one feature distribution into another. This transformation is interpreted as transporting probability mass from one distribution to the other, with the cost proportional to the squared distance

traveled in feature space. Mathematically, the squared Wasserstein-2 distance is defined as

$$W_2^2(\pi_1, \pi_2) = \min_{\gamma_{\mathrm{w}} \in \Gamma(\pi_1, \pi_2)} \int \|\boldsymbol{q} - \boldsymbol{q}^\star\|^2 \, \mathrm{d}\gamma_{\mathrm{w}}(\boldsymbol{q}, \boldsymbol{q}^\star), \tag{S3}$$

where $\Gamma(\pi_1, \pi_2)$ denotes the set of admissible transport plans whose marginals are π_1 and π_2. Each transport plan $\gamma_{\mathrm{w}}(\boldsymbol{q}, \boldsymbol{q}^\star)$ specifies how much probability mass is moved from feature state $\boldsymbol{q}$ in the first flow to feature state $\boldsymbol{q}^\star$ in the second flow. The Wasserstein distance is obtained by selecting the transport plan that minimizes the total transport cost. Intuitively, two flows are considered similar if their feature distributions can be matched with only small displacements in feature space. In practice, the distributions π_1 and π_2 are approximated by empirical measures constructed from finite samples,

$$\pi_1 \approx \frac{1}{N_{\pi_1}} \sum_{i=1}^{N_{\pi_1}} \delta_{\boldsymbol{q}_i}, \qquad \pi_2 \approx \frac{1}{N_{\pi_2}} \sum_{j=1}^{N_{\pi_2}} \delta_{\boldsymbol{q}_j^\star},$$

where $\{\boldsymbol{q}_i\}$ and $\{\boldsymbol{q}_j^\star\}$ are flow features extracted from the corresponding flow fields, and $\delta_{\boldsymbol{q}_i}$ denotes the Dirac delta (unit point-mass) measure concentrated at $\boldsymbol{q}_i$. The resulting empirical measures assign equal probability to each sampled feature vector. The Wasserstein distance between empirical distributions is computed numerically using standard optimal transport solvers and converges to the population distance as the number of samples increases under mild regularity assumptions.

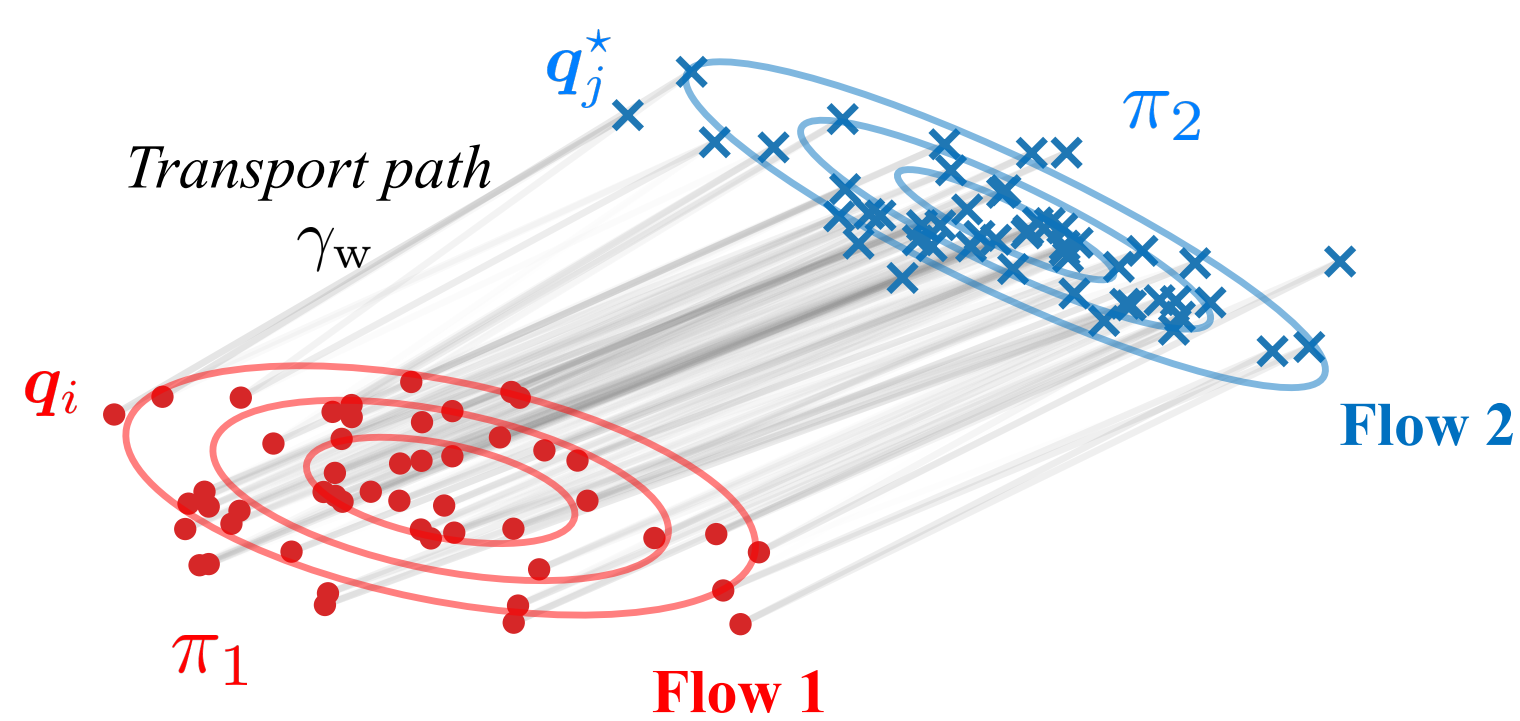

Figure S4: Illustration of similarity between two flow cases based on their feature distributions. Feature samples extracted from the two flows (represented by • and ×) are compared, and gray lines indicate the optimal matching between samples under the optimal transport formulation. The overall dissimilarity between the flows is quantified by aggregating the transport distances between matched feature samples, yielding the distance between their feature distributions.

Building on the distribution-based distance measure defined by the Wasserstein-2 distance, we quantify and interpret flow similarity using hierarchical clustering and distance-preserving low-dimensional embedding. An illustrative example of this procedure is shown in Fig. S18. We first apply hierarchical clustering to the pairwise Wasserstein distances between flow-feature distributions. Starting from individual flow cases, the algorithm iteratively merges the closest clusters, producing the dendrogram shown in Fig. S18a. The resulting hierarchy reveals clear groupings corresponding to secondary flows, separated flows, and free-shear flows, without requiring the number of clusters to be specified *a priori*. To further aid interpretation, we embed the same distance information into a two-dimensional space using multi-dimensional scaling. The distance-preserving embedding retains the relative similarities defined by the Wasserstein-2 distance and provides an intuitive visualization

of how flow cases relate to one another in feature-distribution space, as shown in Fig. S18b. This embedding is invariant under rigid transformations and complements the hierarchical structure by revealing intra- and inter-cluster proximity. Finally, we select representative training cases from each cluster using the cluster medoid, defined as the flow case that minimizes the sum of Wasserstein distances to all other cases within the same cluster. This strategy ensures coverage of dominant flow mechanisms while avoiding redundant cases and limiting training cost. This distribution-based selection provides a principled foundation for joint training and multi-objective learning in the unified turbulence modeling framework.

2.3 Multi-objective ensemble learning from sparse, indirect data

A central contribution of this work is the formulation of turbulence model learning as a multi-objective optimization problem, in which competing objectives arising from different flows and quantities of interest are reconciled within a single unified model. To enable this formulation under the practical constraint of sparse and indirect observations, we combine multi-objective optimization with a regularized ensemble Kalman learning framework, applied to a neural-network–based turbulence model. Within this framework, ensemble-based updates provide robust, gradient-free parameter estimation in the absence of full-field Reynolds-stress data, while the multi-objective formulation balances performance across heterogeneous flow regimes. Together, these elements yield a scalable and physically grounded strategy for constructing unified turbulence models from diverse data sources.

2.3.1 Multi-objective learning for unified turbulence modeling

A unified turbulence model should deliver robust and accurate predictions across diverse flows without manual intervention. To this end, we train a single model using heterogeneous data, such as sparse velocity measurements and integral forces, drawn from flows with distinct physical mechanisms, including attached, separated, secondary, and free-shear flows. This formulation naturally gives rise to multiple objectives that must be optimized simultaneously. Accordingly, the learning problem is formulated as a multi-objective optimization problem, where each flow case and quantity of interest defines an individual objective with loss function ℓ_j:

$$\min_{\mathbf{w}} \left[\ell_1(\mathbf{w}),\, \ell_2(\mathbf{w}),\, \ldots,\, \ell_N(\mathbf{w})\right]^\top ,$$

where $\mathbf{w}$ denotes the weights of the neural-network-based turbulence model (Fig. S2). This formulation explicitly recognizes that improving performance for one objective may degrade performance for others, leading to conflicting objectives during optimization.

Such conflicts arise when different quantities of interest (QoIs) impose different constraints on the turbulence closure. Importantly, QoIs from the same flow are not necessarily aligned and can also be conflicting, as will be demonstrated in §3.2.2. The nature and extent of this conflict are also model-dependent, since different baseline models can exhibit different physical biases and thus different QoI sensitivities. This motivates a multi-objective learning framework for handling competing QoI constraints within a unified turbulence model.

In multi-objective learning, Pareto optimality refers to solutions for which no objective can be improved without worsening at least one other objective. For unified turbulence modeling, a Pareto-optimal solution corresponds to a parameter vector $\mathbf{w}$ that achieves a balanced compromise across multiple flows, rather than optimal performance on a single case. A common approach to multi-objective learning is to combine objectives into a single weighted loss; however, fixed-weight formulations suffer from two major limitations. First, suitable weights are not known *a priori* and often require costly grid searches or heuristic tuning [43, 44]. Second, fixed weighting provides

no principled notion of optimality in the presence of conflicting objectives, frequently resulting in solutions that favor some flows at the expense of others.

To overcome these limitations, we adopt a multi-gradient descent framework with dynamically adjusted weights [45]. Network weights of the model are updated according to

$$\mathbf{w} \leftarrow \mathbf{w} - \eta \sum_{j=1}^{N} \xi_j \nabla_{\mathbf{w}} \ell_j(\mathbf{w}),$$

where η is the learning rate and ξ_j denotes the weight associated with objective j. Pareto optimality can be characterized through Pareto stationarity using the Karush–Kuhn–Tucker (KKT) conditions [46]. A network weight vector $\mathbf{w}$ is Pareto stationary if there exists a nonnegative weight vector $\boldsymbol{\xi} = [\xi_1, \xi_2, \ldots, \xi_N]^\top$, with $\sum_{j=1}^{N} \xi_j = 1$, such that

$$\sum_{j=1}^{N} \xi_j \nabla_{\mathbf{w}} \ell_j(\mathbf{w}) = \mathbf{0}.$$

This condition implies that the objective gradients are in conflict unless all gradients vanish simultaneously. The central task of multi-objective optimization is therefore to identify the weight vector $\boldsymbol{\xi}$ that reconciles these conflicts. In practice, vector $\boldsymbol{\xi}$ can be determined by solving an auxiliary optimization problem that minimizes the norm of the weighted sum of objective gradients. A zero norm indicates Pareto stationarity, while a nonzero norm indicates that objectives are locally aligned and can be improved simultaneously. Each gradient is first normalized by the product of its loss value and its L^2 norm to ensure meaningful weighting across heterogeneous objectives. After normalization, objectives with smaller losses produce larger normalized gradients and therefore receive smaller weights. This mechanism automatically emphasizes underperforming objectives while preventing well-optimized objectives from dominating the update. The resolution of conflicts in multi-objective optimization is illustrated in Fig. S5. We use $\boldsymbol{G}_j = \nabla_{\mathbf{w}} \ell_j(\mathbf{w})$ to denote the gradient of objective j, and $\overline{\boldsymbol{G}} = \sum_{j=1}^{N} \xi_j \boldsymbol{G}_j$ is their weighted sum. The Frank–Wolfe algorithm [47] is used to optimize $\boldsymbol{\xi}$ by identifying the objective gradient most misaligned with $\overline{\boldsymbol{G}}$ and increasing its weight, while maintaining the normalization constraints. Compared with derivative-free methods such as non-dominated sorting genetic algorithms, this gradient-based approach scales efficiently to problems with many objectives [48], making it well suited for unified turbulence model training.

2.3.2 Regularized ensemble learning from sparse, indirect observations

In practical turbulence modeling, most experimental and industrial datasets provide only sparse and indirect observations, such as velocities or integral forces, rather than full-field Reynolds stress data. Learning from such indirect data with gradient-based methods typically requires adjoint-enabled or fully differentiable solvers, which are often unavailable in complex industrial applications. To enable stable and efficient learning under these conditions, we adopt a regularized ensemble Kalman filter (REnKF) method [49].

Turbulence model learning is formulated as a Bayesian inverse problem: given observations $\mathbf{y}$ and a baseline model as the prior, the goal is to infer the posterior distribution of the turbulence model, parameterized by neural network weights $\mathbf{w}$,

$$\mathbb{P}(\mathbf{w} \mid \mathbf{y}) \propto \mathbb{P}(\mathbf{w})\, \mathbb{P}(\mathbf{y} \mid \mathbf{w}).$$

Under Gaussian and linear assumptions, the maximum a posteriori estimate reduces to the optimization problem

$$\ell_{\mathrm{EnKF}}(\mathbf{w}) = \|\mathbf{w}^{i+1} - \mathbf{w}^{i}\|^2_{\mathsf{P}^{-1}} + \|\mathbf{y} - \mathcal{H}[\mathbf{w}^{i+1}]\|^2_{\mathsf{R}^{-1}}, \tag{S4}$$

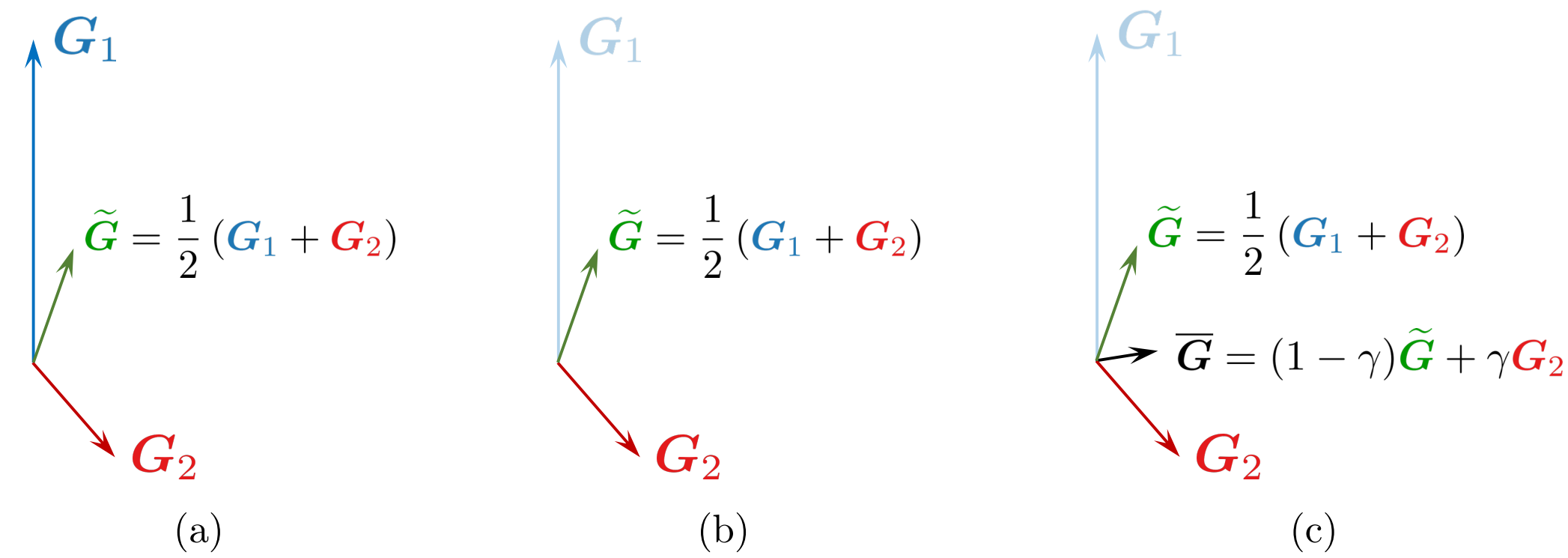

Figure S5: Illustration of the Frank–Wolfe algorithm for two conflicting objectives $\boldsymbol{G}_1$ and $\boldsymbol{G}_2$: (a) averaging objective gradients to obtain the mean gradient $\widetilde{\boldsymbol{G}}$, (b) identifying the most conflicting gradient $\boldsymbol{G}_2$, and (c) under-relaxing the mean gradient toward the conflicting gradient using a step size γ. The procedure iterates until conflicts are resolved or a stopping criterion is met.

where $\mathcal{H}$ is the observation operator, and P and R denote the covariance matrices of the prior model and observation errors, respectively. Iterative EnKF tends to push the solution away from the baseline, effectively reducing the update to maximum likelihood estimation and often leading to overfitting and poor generalization.

To mitigate this issue, we introduce a regularization term into Eq. (S4) that penalizes deviations from physically meaningful baseline model coefficients. The resulting regularized loss function is

$$\ell_{\text{REnKF}}(\mathbf{w}) = \ell_{\text{EnKF}}(\mathbf{w}) + \|\mathcal{G}[\mathbf{w}^{i+1}]\|^2_{\mathsf{Q}^{-1}},$$

where Q denotes the covariance matrix of the regularization constraints. The regularization operator $\mathcal{G}$ penalizes deviations of selected model coefficients from their baseline values. For the constitutive relation, the baseline linear eddy-viscosity coefficients are $g^{(1)} = -0.09$ and $g^{(2)} = 0$, while for the turbulence transport equations the dissipation coefficient β has a baseline value of 0.072. Accordingly, the regularization operator is defined as

$$\boldsymbol{\mathcal{G}} = \left[(g^{(1)} + 0.09)^2,\ (g^{(2)} - 0)^2,\ (\beta - 0.072)^2\right].$$

Minimizing the regularized cost function ℓ_{REnKF} yields a two-stage update scheme [49, 50]. First, a pre-correction step accounts for the regularization term,

$$\widetilde{\mathbf{w}}^i_j = \mathbf{w}^i_j - \mathsf{P}\,\mathcal{G}'[\mathbf{w}^i_j]^\top \mathsf{Q}^{-1}\mathcal{G}[\mathbf{w}^i_j],$$

followed by the standard ensemble Kalman update,

$$\mathbf{w}^{i+1}_j = \widetilde{\mathbf{w}}^i_j + \mathsf{K}\big(\mathbf{y}_j - \mathcal{H}[\widetilde{\mathbf{w}}^i_j]\big), \quad \mathsf{K} = \mathsf{P}\mathsf{H}^\top \left(\mathsf{H}\mathsf{P}\mathsf{H}^\top + \mathsf{R}\right)^{-1}.$$

Overall, this method stabilizes learning of nonlinear eddy-viscosity models, preserves physical consistency between the constitutive relation and transport equations, and yields turbulence models that are both robust and generalizable.

Within this regularized ensemble Kalman framework, the remaining question is how multiple, potentially competing objectives are incorporated consistently into the ensemble updates. This is achieved naturally, as each objective j yields an ensemble-based update $\boldsymbol{G}_j$ which can be interpreted

as a generalized gradient direction. These objective-specific updates are combined using adaptive weights $\boldsymbol{\xi}$ obtained from the multi-objective optimization,

$$\mathbf{w} \leftarrow \mathbf{w} + \sum_{j=1}^{N} \xi_j \boldsymbol{G}_j,$$

thereby coupling multi-objective learning with the regularized ensemble Kalman method. The complete multi-objective ensemble learning procedure is summarized in Algorithm 1.

Algorithm 1 Multi-objective ensemble learning ($T = 100$)

---

for $t = 1, 2, \ldots, T$ **do**
  Compute individual objective j's update $\boldsymbol{G}_j$ by regularized ensemble Kalman method
  Normalize each update:

$$\mathsf{G}_j = \frac{\boldsymbol{G}_j}{\ell_j \cdot \|\boldsymbol{G}_j\|}$$

  Initialize task weights: $\boldsymbol{\xi} \leftarrow [1/N, \ldots, 1/N]$
  repeat
    Compute weighted mean gradient:

$$\overline{\mathsf{G}} = \sum_{j=1}^{N} \xi_j \mathsf{G}_j$$

    Identify the most conflicting task:

$$J = \arg\min_j \mathsf{G}_j^\top \overline{\mathsf{G}}$$

    Compute step size γ via line search:

$$\gamma = \arg\min_{\gamma \in [0,1]} \left\| (1-\gamma)\overline{\mathsf{G}} + \gamma \mathsf{G}_J \right\|^2$$

    Update task weights (where $\mathbf{e}_J \in \mathbb{R}^N$ denotes the Jth unit vector):

$$\boldsymbol{\xi} \leftarrow (1-\gamma)\boldsymbol{\xi} + \gamma \mathbf{e}_J$$

  until Convergence of $\boldsymbol{\xi}$ or maximum steps reached
  Update network weights $\mathbf{w}$ with learning rate η:

$$\mathbf{w} \leftarrow \mathbf{w} + \eta \left(\sum_{j=1}^{N} \xi_j \boldsymbol{G}_j \right)$$

end for
return Final network weights $\mathbf{w}$

2.4 Additive fine-tuning for specialist model

When application-specific accuracy is required, the unified foundation model can be adapted into a specialist model via additive fine-tuning. A unified foundation model is designed to perform robustly across diverse flow mechanisms and typically improves upon baseline closures over a wide range of conditions. However, this broad objective inevitably trades off accuracy in flow regimes that dominate a specific device or operating condition. Enforcing uniform performance across many regimes can dilute model capacity for the mechanisms of primary interest, such as large-scale separation in Ahmed body flows or strong secondary motions in three-dimensional diffusers. In these settings, higher predictive accuracy is often achieved by relaxing performance requirements on less relevant regimes and concentrating model capacity on the targeted mechanisms. This motivates the introduction of a *specialist model*, which improves accuracy within a specific mechanism class while retaining limited generalizability. Crucially, such specialization must preserve essential physical properties inherited from the unified foundation model, such as the logarithmic law of the wall.

The unified foundation model is treated as a strong prior that already encodes broad extrapolation capability across canonical flows through multi-objective training. Specialization is therefore formulated as a controlled adaptation that enhances performance on a targeted application domain while introducing the smallest possible deviation from the unified foundation model. To this end, an additive parameterization is adopted, in which the foundation network is frozen and only a compact correction module is trained (Fig. S2 c). Let the unified foundation model predict the closure quantities $\{g_{\mathrm{f}}^{(1)}, \cdots, g_{\mathrm{f}}^{(10)}, \beta_{\mathrm{f}}\}$ using the physically consistent parallel TBNN architecture. The specialist model augments these predictions by learning small additive corrections $\{\Delta g^{(1)}, \cdots, \Delta g^{(10)}, \Delta\beta\}$, such that

$$g_{\mathrm{s}}^{(i)} = g_{\mathrm{f}}^{(i)} + \Delta g^{(i)}, \quad \beta_{\mathrm{s}} = \beta_{\mathrm{f}} + \Delta\beta. \tag{S5}$$

Only the correction networks are trainable and the unified foundation model remains fixed. This design ensures that the specialist model (i) starts from a well-validated foundation and (ii) modifies the closure only where required by the new data. Additive learning thus provides a natural mechanism for capturing flow-specific effects while retaining the robustness of the unified foundation model. Each correction term is parameterized by a fully connected neural network. The sub-networks for $\Delta g^{(1)}$, $\Delta g^{(2)}$, and $\Delta\beta$ each use four hidden layers with four neurons per layer and employ the ReLU activation function. In total, the additive fine-tuning architecture introduces 255 trainable network weights.

In the fine-tuning process, the physical consistency is still preserved. The specialist model continues to predict β_{s} together with the constitutive coefficients $g_{\mathrm{s}}^{(i)}$, while the remaining transport coefficients $(\beta^*, \alpha, \sigma, \sigma^*)$ are computed through the same constraint chain used for the foundation model, including equilibrium homogeneous shear behavior and logarithmic-layer compatibility. As a result, key physical properties, such as the logarithmic velocity profile, are preserved by construction. This guarantees that fine-tuning improves performance on targeted flows without introducing unphysical inconsistencies.

The specialist model is fine-tuned on a set of targeted flows, supplemented by closely related canonical cases, using the same multi-objective learning framework adopted for foundation training. Each flow and quantity of interest defines an individual objective, and conflicts among objectives are resolved through adaptive weighting. Denoting the trainable correction network weights by $\mathbf{w}_{\mathrm{s}}$ and the frozen unified foundation model network weights by $\mathbf{w}_{\mathrm{f}}$, the fine-tuning problem is formulated as

$$\min_{\mathbf{w}_{\mathrm{s}}} \sum_{j=1}^{N_s} \xi_j \, \ell_j(\mathbf{w}_{\mathrm{f}}, \mathbf{w}_{\mathrm{s}}),$$

with the objective-specific loss

$$\ell_j(\mathbf{w}_\mathrm{f}, \Delta\mathbf{w}) = \|\mathbf{w}_\mathrm{s}^{i+1} - \mathbf{w}_\mathrm{s}^{i}\|^2_{\mathsf{P}^{-1}} + \|\mathbf{y} - \mathcal{H}[\mathbf{w}_\mathrm{f}, \mathbf{w}_\mathrm{s}^{i+1}]\|^2_{\mathsf{R}^{-1}} + \|\mathcal{G}[\mathbf{w}_\mathrm{s}^{i+1}]\|^2_{\mathsf{Q}^{-1}},$$

where ℓ_j follows the same regularized ensemble learning formulation introduced earlier. The regularization operator $\boldsymbol{\mathcal{G}}$ penalizes deviations of the additive corrections from zero and is defined as

$$\boldsymbol{\mathcal{G}} = \left[\left(\Delta g^{(1)}\right)^2, \cdots, \left(\Delta g^{(10)}\right)^2, \ (\Delta\beta)^2\right].$$

This formulation enforces a *minimal-modification* principle: additive corrections are activated only when they yield significant improvements in the objectives.

When multiple candidate fine-tuning cases are available, priority is given to those that are statistically closest to the target application in the model input feature space. Similarity between flows is quantified using probability distribution distances between their feature distributions, and only cases with the smallest distances to the target flows are selected. This strategy promotes mechanism-consistent specialization—for example, selecting curved-step, periodic-hill, or bump flows to support separation-dominated targets—and empirically improves generalization within closely related configurations.

3 Verification and validation

Physical consistency and multi-objective learning are essential for constructing unified turbulence models that are both reliable and broadly applicable. Physical consistency is verified by demonstrating that the learned model preserves the logarithmic law of the wall and is validated by improved predictions in a round jet flow, where turbulence transport coefficients strongly govern flow development. We also demonstrate that quantities of interest for model training can be conflicting even within the same flow, highlighting the necessity of multi-objective learning. The effectiveness of multi-objective learning is demonstrated by its ability to reconcile conflicting objectives in periodic hill flows, maintain performance in non-conflicting square duct and airfoil cases, and scale robustly to an increasing number of training objectives while achieving Pareto-improved solutions.

3.1 Effectiveness of physical consistency

We examine both the enforcement and practical impact of physical consistency in the turbulence model. Correct implementation of the imposed constraints is first verified by assessing whether the learned model preserves fundamental theoretical behavior, namely the logarithmic law of the wall. The influence of physical consistency on predictive accuracy is then evaluated by comparing physically consistent and inconsistent models on a round jet flow, a configuration in which turbulence transport coefficients strongly govern flow development.

3.1.1 Verification of physical consistency within turbulence model

Physical consistency is assessed in both the inner and outer regions of the boundary layer. In the inner region, we verify whether the learned model preserves the logarithmic law of the wall.

As derived from the physical constraint based on equilibrium log-layer analysis, the logarithmic relationship

$$u^+ = \frac{1}{\kappa}\ln y^+ + C \approx \frac{2.3}{\kappa}\log_{10} y^+ + C$$

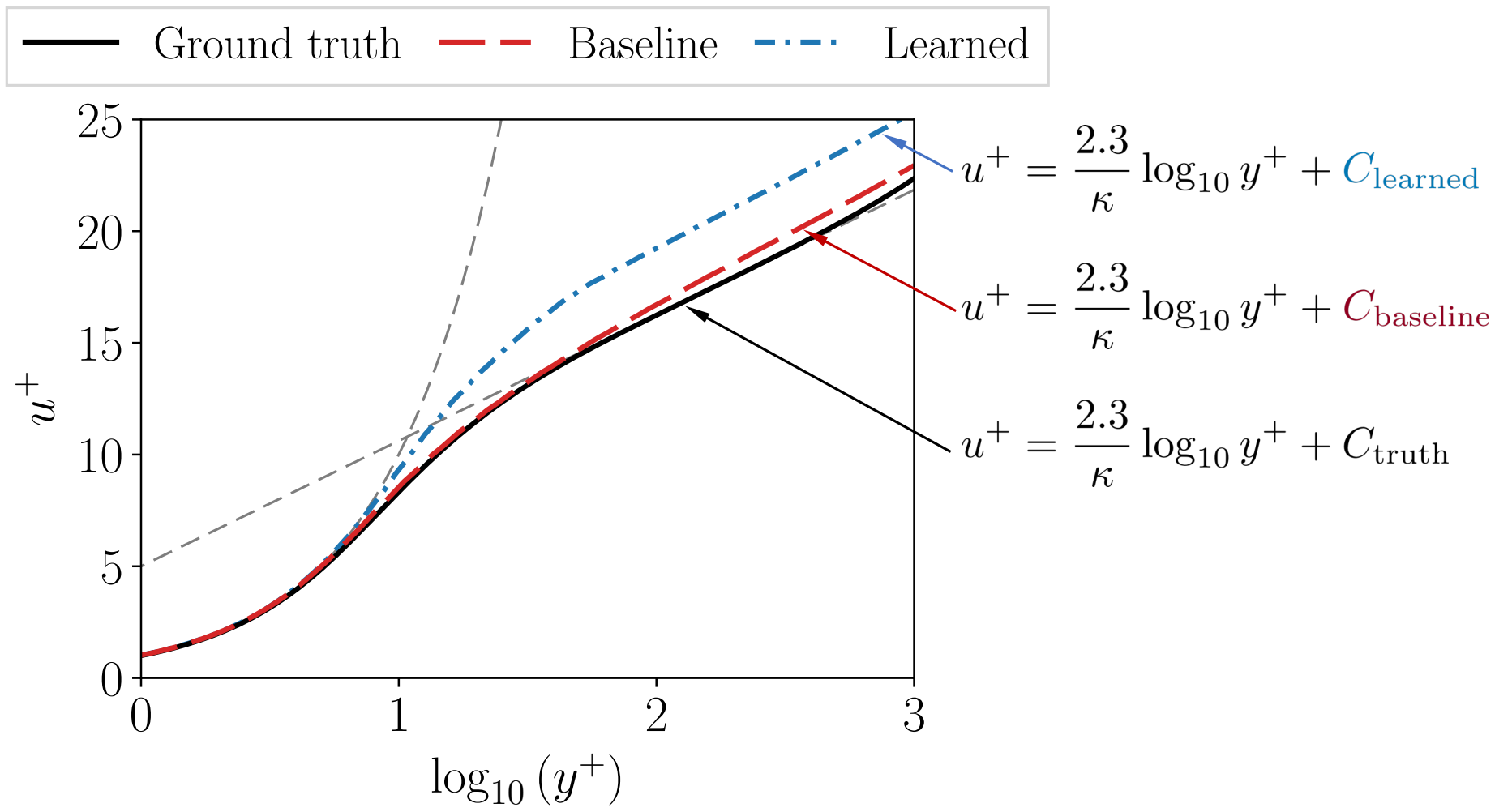

Figure S6: Verification of physical consistency of the learned model using a turbulent flat plate. Mean velocity profiles u^+ are shown versus the wall-normal coordinate y^+ on a logarithmic scale for the learned model (the specialist model for secondary flows with separation), baseline k–ω model, and ground truth. The learned model exhibits the same logarithmic slope in the log layer as the baseline model and the ground truth, consistent with the imposed von Kármán constant $\kappa = 0.41$. Differences arise only in the additive constant (log-law intercept), resulting in a vertical shift of the logarithmic velocity profile.

is enforced through the fixed slope ($1/\kappa$ for $\ln y^+$, equivalently $2.3/\kappa$ for $\log_{10} y^+$), where κ is the von Kármán constant, while the additive constant C is not explicitly constrained. To verify correct enforcement, the learned model (the specialist model for secondary flows with separation) is evaluated on a turbulent flat plate. Within the logarithmic region, the predicted relationship between u^+ and y^+ remains logarithmic and preserves the correct slope, confirming that the imposed constraint is respected (see Fig. S6). A vertical shift of the logarithmic profile is observed, which is as expected because the intercept is not specified by the constraint. This behavior is consistent with conventional turbulence modeling practice [34, 51], where both the slope and intercept are calibrated empirically rather than derived from first principles, and therefore confirms correct and reasonable enforcement of the law of the wall. If enforcing the intercept is desired, the turbulent flat plate can be included explicitly as a training case to constrain the additive constant.

To further assess the outer region, we examine the mean streamwise velocity profiles using outer scaling, y/δ versus U/U_∞, where U_∞ is the freestream velocity and δ is the 99% boundary-layer thickness. A small deviation is observed between the learned and baseline profiles, arising from differences in the logarithmic-law intercept C in the inner layer. Despite this offset, the profiles exhibit consistent trends, indicating that the learned model preserves the global boundary-layer structure (see Fig. S7). Together, the inner- and outer-layer analyses demonstrate that the model maintains physical consistency across the entire boundary layer.

3.1.2 Validation of physically consistent model performance

The impact of physical consistency on predictive performance is validated using a round jet flow, a canonical configuration known to be sensitive to turbulence transport coefficients. For the baseline k–ω model, the dissipation coefficient β in the ω equation strongly influences the jet spreading rate and velocity decay. To isolate the effect of physical consistency, we mainly compare predictions

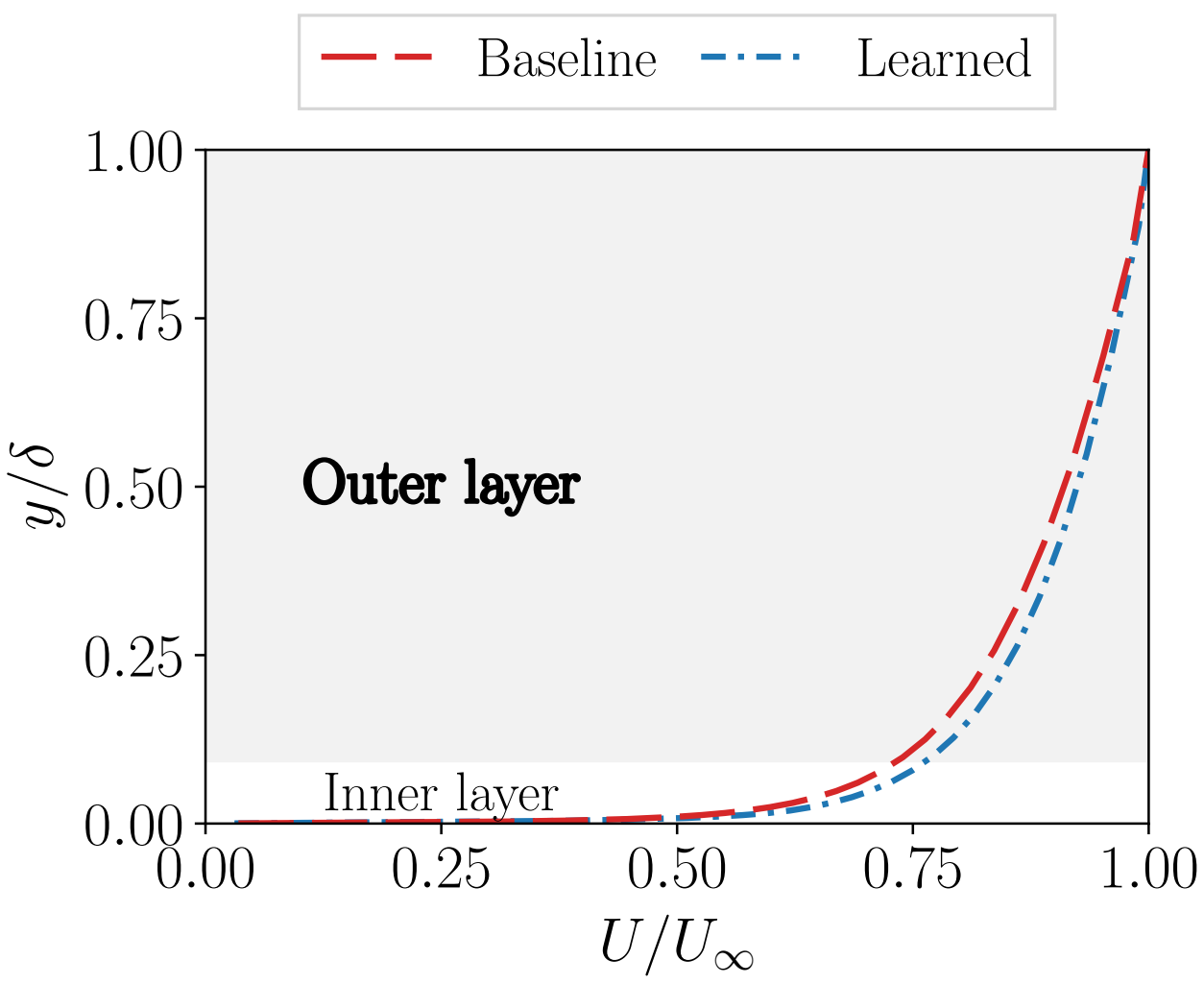

Figure S7: Outer-scaled streamwise velocity profiles for the turbulent flat plate, shown as y/δ versus U/U_∞, where U is the streamwise velocity, U_∞ is the freestream velocity, y is the wall distance, and δ is the 99% boundary-layer thickness.

from two models: one that enforces coupled learning of the constitutive relation and turbulence transport equations under physical constraints, and one that does not. The physically consistent model closely matches the ground truth, yielding an improved prediction of centerline velocity decay in the jet flow (see Fig. S8). In contrast, the physically inconsistent model shows pronounced discrepancies downstream of the jet exit, where the velocity decay is noticeably delayed over an extended region. Its decay trend closely follows that of the baseline model, as the two velocity profiles remain nearly parallel. The superior agreement achieved by the physically consistent model underscores the importance of enforcing consistent coupling between the constitutive relation and the turbulence transport equations in jet flows.

3.2 Characteristics of aligned and conflicting quantities of interest

Quantities of interest (QoIs) from the same flow are not necessarily aligned and can be conflicting. Such conflicts arise because different QoIs probe different physical sensitivities and therefore favor different model corrections. The S809 airfoil provides a representative example, where lift and drag induce opposing corrections to the same closure term. By contrast, the curved-step flow shows that QoIs can also remain aligned, with the velocity field and skin-friction coefficient improving consistently under the learned model. Together, these cases show that QoI compatibility depends on both the flow physics and the deficiencies of the baseline model.

3.2.1 Aligned quantities of interest: velocity and friction coefficient of curved step

Aligned quantities of interest are quantities that are governed by the same dominant flow mechanism and therefore respond to model changes in a consistent manner. When two quantities depend on the same physical process, improvements in the prediction of that process are expected to affect both quantities in a similar way. In this sense, aligned QoIs do not compete but instead provide complementary views of the same underlying flow physics.

The curved step flow provides a representative example. In this case, the dominant mechanism is separation and reattachment induced by the curved geometry and the associated adverse pressure

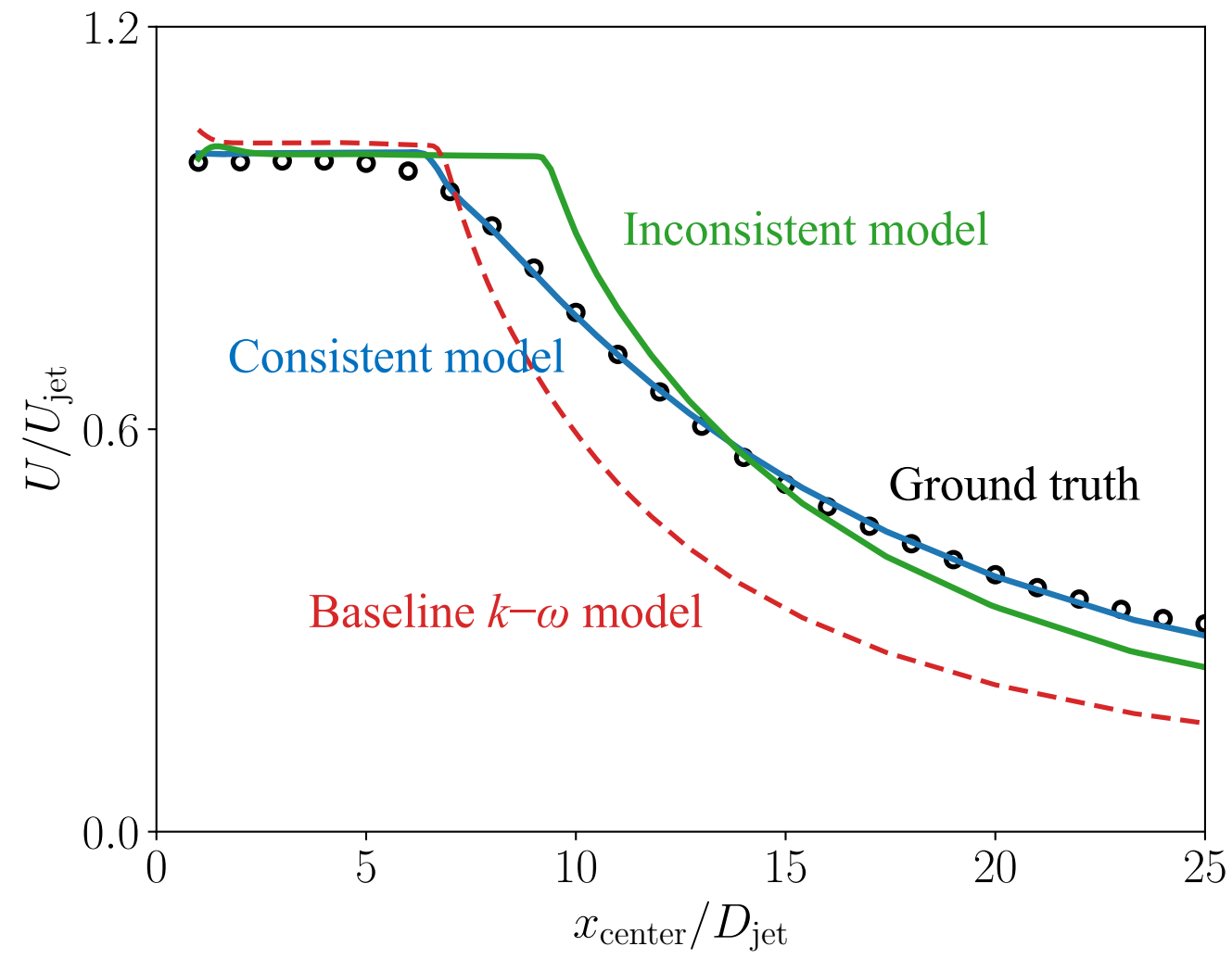

Figure S8: Verification of physical consistency using a round jet flow. The centerline velocity U, normalized by the jet exit velocity U_{jet}, is plotted against the normalized downstream distance $x_{\mathrm{center}}/D_{\mathrm{jet}}$, where D_{jet} is the jet diameter, for the physically consistent model, physically inconsistent model, baseline k–ω model, and ground truth. The physically consistent model closely reproduces the decay trend of the ground truth along the jet centerline, whereas the physically inconsistent and baseline models deviate downstream.

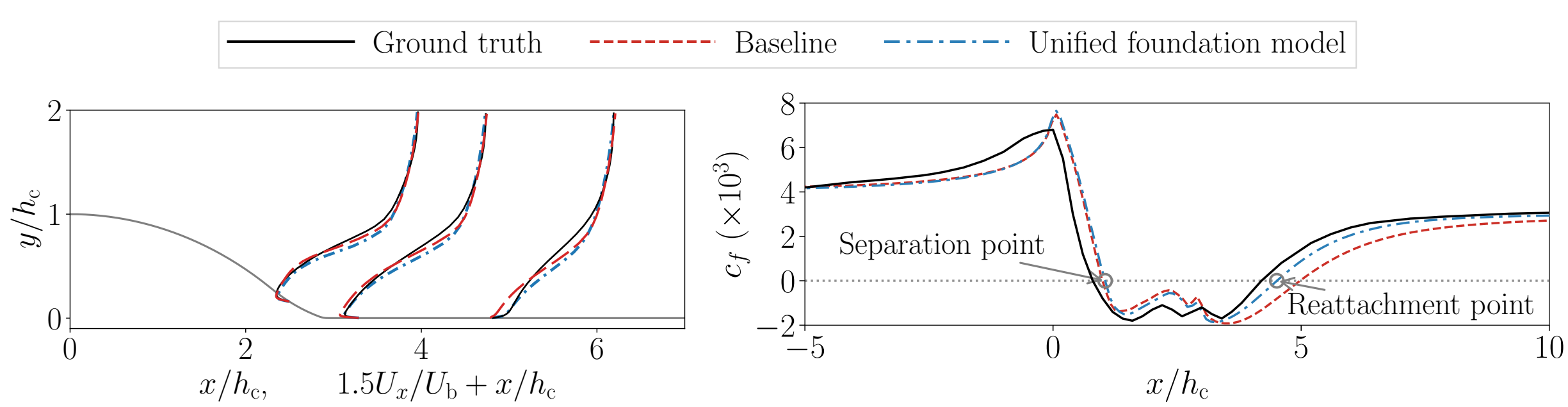

(a) Streamwise velocity profiles

(b) Friction coefficient

Figure S9: Performance evaluation of the **unified foundation model** in a **curved step flow** against the ground truth and the baseline k–ω model. (a) Streamwise velocity profiles U_x, normalized by the bulk velocity U_b, are shown at multiple streamwise locations downstream of the curved step. The profiles U_x versus the wall-normal coordinate y normalized by the curved-step height h_c. (b) Skin-friction coefficient distributions c_f, are shown along the streamwise direction x/h_c. Separation and reattachment points are marked using circles.

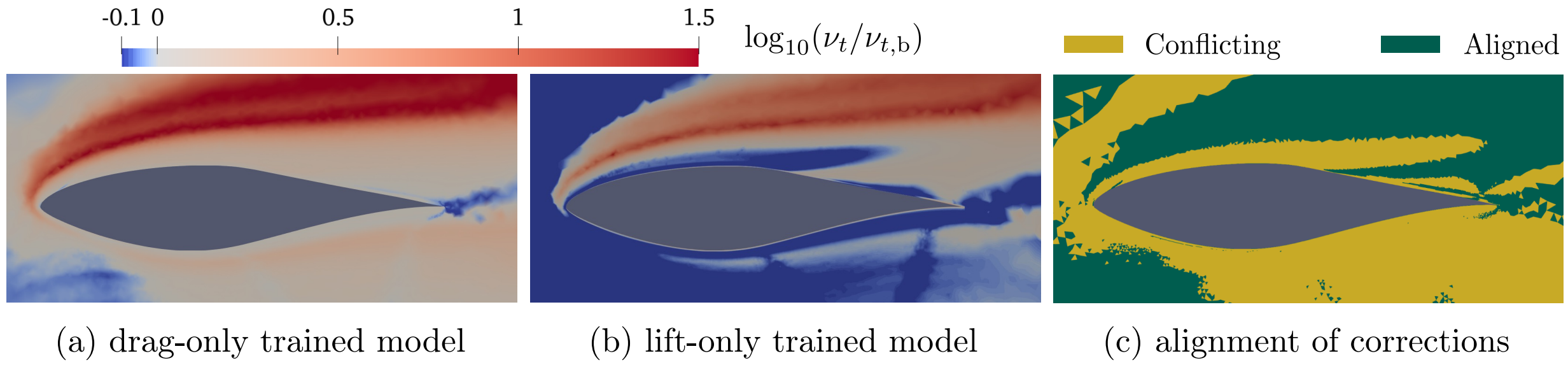

Figure S10: Comparison of eddy-viscosity corrections obtained from single-objective training and their mutual consistency. Panels (a) and (b) show the predicted eddy viscosity, $\log_{10}(\nu_t/\nu_{t,\mathrm{b}})$, from drag-only and lift-only training, respectively, where $\nu_{t,\mathrm{b}}$ denotes the baseline eddy viscosity. Panel (c) highlights regions of alignment and conflict of these corrections.

gradient. The key modeling challenge is therefore to predict the development of the separated shear layer, the extent of the recirculation region, and the downstream recovery of the flow. To constrain these features, we use streamwise velocities sampled at multiple downstream locations as observational data. As shown in Fig. S9a, the unified foundation model yields velocity profiles that are generally closer to the ground truth than the baseline, particularly in the separated and recovery regions, where the shape of the shear layer and the progression toward reattachment are better represented.

The alignment between the velocity field and the skin-friction coefficient c_f is reflected in the corresponding wall-friction distribution shown in Fig. S9b. The unified model produces a c_f distribution that more closely follows the ground-truth trend compared to the baseline, particularly in the separated and recovery regions. In particular, it shows a more consistent representation of the negative region associated with reversed flow and a smoother recovery downstream of reattachment, although discrepancies remain near the peak and separation onset. Overall, the changes observed in both the velocity field and c_f follow similar trends. This behavior is consistent with the fact that both quantities are controlled by the same separation and reattachment physics, suggesting that improvements in the learned model primarily act through correcting this shared mechanism.

3.2.2 Conflicting quantities of interest: lift and drag of S809 airfoil

Conflicting quantities of interest (QoIs) arise when different target quantities favor different corrections to the turbulence closure, even within the same flow configuration. In such situations, improving one QoI requires adjusting the flow in a direction that does not improve, and can even deteriorate, another QoI. The source of the conflict therefore does not necessarily lie in using different flows; rather, it originates from the different physical sensitivities encoded by distinct QoIs within the same flow. Importantly, the manifestation of such conflicts is also model-dependent: different baseline turbulence models may produce different deficiencies in the predicted flow field, and therefore induce different QoI sensitivities and preferred correction directions. As a result, whether two QoIs appear aligned or conflicting, and how strongly that conflict is expressed, depends not only on the flow physics but also on the baseline model from which the correction is learned.

In the present S809 airfoil case, the baseline k–ω model overpredicts the lift coefficient and underpredicts the drag coefficient. The baseline solution remains overly attached on the suction side, maintaining excessive suction and thus yielding too much lift. This result is consistent with the interpretation presented in [32]. The baseline model also underpredicts the wake deficit, which leads to an underprediction of drag, reflecting a known limitation of k–ω type models in capturing mixing

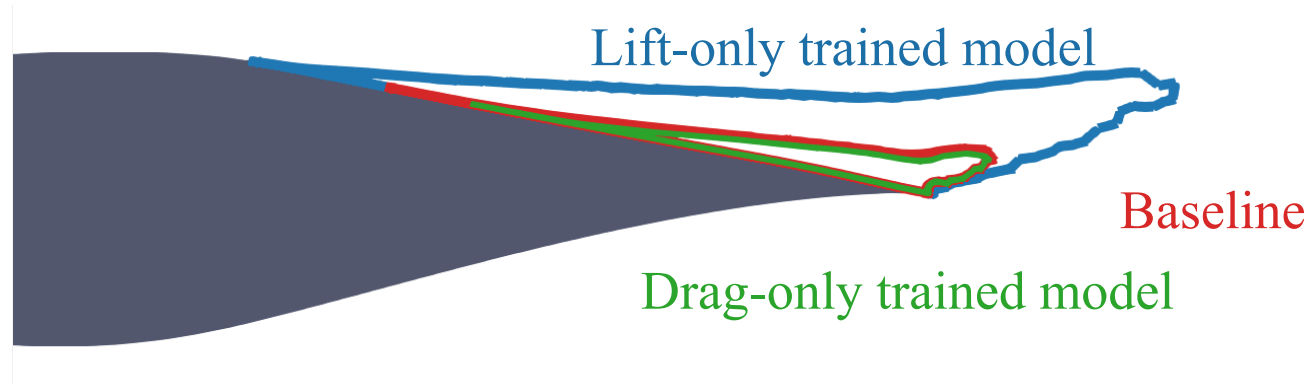

Figure S11: Comparison of the predicted separation bubble for the S809 airfoil obtained from three models: drag-only trained model, baseline model and lift-only trained model.

and shear-layer development in separated and free-shear flows.

The results of the single-objective learning cases show that lift and drag drive the model toward opposite eddy-viscosity corrections, as shown in Fig. S10c. In particular, the lift-only training case yields a much smaller eddy viscosity especially near the airfoil compared to the baseline (Fig. S10b), whereas the drag-only training case yields a much larger eddy viscosity ν_t (Fig. S10a). This indicates that the two objectives favor different turbulence levels, and therefore different flow-adjustment mechanisms.

The learned behavior is dominated by the linear coefficient $g^{(1)}$, since the quadratic coefficient $g^{(2)}$ remains negligible in both cases and contributes little to the closure. As a result, the difference between the learned models can be interpreted primarily as different corrections to the turbulent eddy viscosity. In the present formulation, this relationship is given by

$$\nu_t = -\frac{g^{(1)}k}{C_\mu \omega}.$$

Changes in coefficient $g^{(1)}$ therefore translate directly into changes in turbulent eddy viscosity. The opposite eddy viscosity ν_t corrections obtained from lift-only and drag-only training (Fig. S10c) thus imply opposing adjustments to the dominant closure coefficient $g^{(1)}$, providing strong evidence that the optimization directions associated with these two QoIs are conflicting. These opposite corrections correspond to two distinct physical pathways.

In the lift-only case, the learned model predicts earlier separation and a substantially larger separation bubble than the baseline solution (Fig. S11), which reduces the suction-side pressure loading and brings the lift coefficient closer to the ground truth. This behavior arises from a redistribution of eddy viscosity. Specifically, the learned model decreases eddy viscosity ν_t in the near-wall region on the suction side, while increasing it in the detached shear layer above the separated region. The reduced near-wall eddy viscosity ν_t weakens turbulent momentum transfer into the boundary layer under the adverse pressure gradient, making the flow less resistant to separation. At the same time, the elevated eddy viscosity ν_t in the detached shear layer enhances mixing after separation and helps regulate the downstream development of the enlarged separated flow. The correction therefore acts through a spatial redistribution of turbulent mixing: weaker near-wall mixing to permit stronger separation, and stronger shear-layer mixing once the flow has lifted off. This mechanism remains qualitatively consistent with the separation-based interpretation discussed in [32], while indicating that, in the present case, the learned correction is better understood as a spatial reorganization of ν_t relative to the baseline.

The drag-only training delays separation and produces a slightly smaller separation bubble than the baseline (Fig. S11). At the same time, it generates a stronger and more extended wake deficit, as indicated by the broad blue region of negative $\log_{10}(U/U_{\text{baseline}})$ that persists farther downstream in Fig. S12. This pattern indicates that the drag-only model predicts lower wake velocity than the baseline over much of the wake, with both greater streamwise extent and wider spreading. A localized

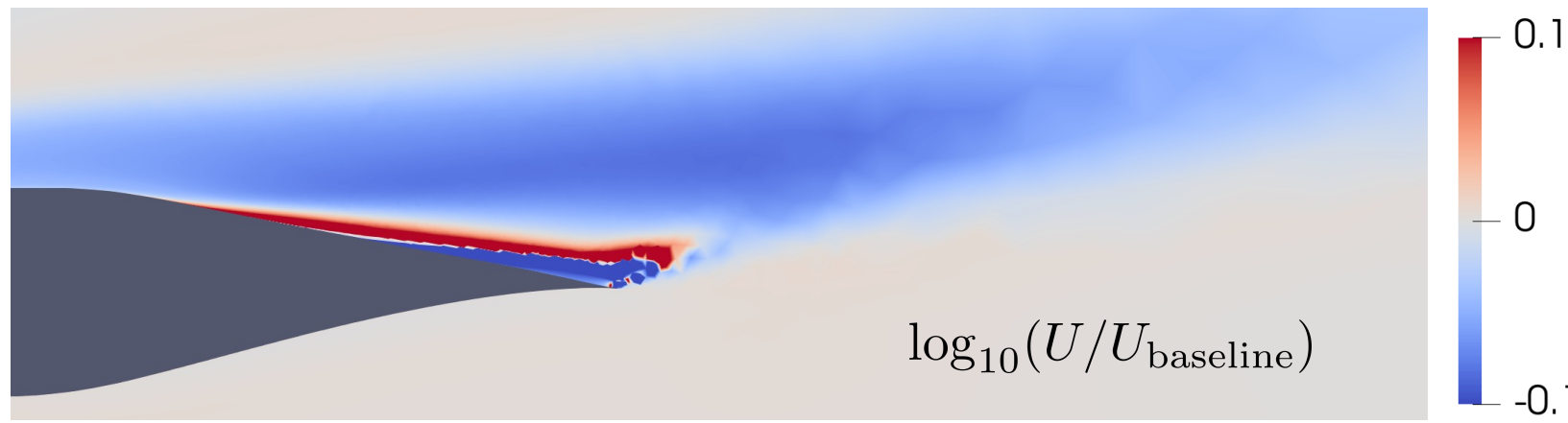

Figure S12: Comparison of the wake deficit between the model trained with only drag and the baseline model for the S809 airfoil. The velocity ratio $\log_{10}(U/U_{\mathrm{baseline}})$ highlights the regions of velocity deficit and acceleration compared to the baseline model.

red region is also observed near the suction-side shear layer and close to the airfoil, indicating limited regions where the drag-only model predicts slightly higher velocity than the baseline. Overall, this behavior is associated with a more global increase in ν_t, including the upper shear layer, the wake, and much of the near-airfoil region, which enhances turbulent mixing and downstream dissipation. Force decomposition further shows that the drag increase arises entirely from pressure drag rather than viscous drag. The model therefore increases drag not by enhancing wall shear, but by modifying the pressure field through stronger wake dissipation. In this sense, separation and drag are not directly equivalent: the drag increase is achieved primarily through wake modification rather than stronger separation.

Taken together, these results demonstrate that lift and drag are intrinsically conflicting quantities of interest in the present airfoil problem. The lift-only objective drives the dominant closure coefficient $g^{(1)}$, and thus eddy viscosity ν_t, in a direction that promotes earlier separation and enlarges the recirculation zone. In contrast, the drag-only objective drives $g^{(1)}$ in the opposite direction, increasing eddy viscosity ν_t in the shear layer and wake and thereby increasing pressure drag without requiring a larger separation bubble. These two QoIs therefore favor opposing corrections to the same dominant closure mechanism, leading to distinct physical behaviors of the flow.

More generally, each quantity of interest encodes specific aspects of the underlying flow physics. In canonical flows, a QoI often reflects a single dominant mechanism, whereas in complex flows, it can combine multiple interacting mechanisms. When model calibration relies on a single QoI, the resulting model is exposed to only a subset of the underlying physics. As a result, it may capture only partial mechanisms and exhibit restricted generalizability, reminiscent of the parable of the six blind men describing an elephant from incomplete perspectives.

3.3 Effectiveness of multi-objective learning

We validate the proposed multi-objective learning framework under two distinct scenarios: a conflict scenario and a non-conflict scenario. In the conflict scenario, the framework automatically resolves competing objectives arising from two periodic hill cases with different separation characteristics, as shown in Fig. S14. In the non-conflict scenario, which includes an S809 airfoil and a square duct flow, the framework preserves optimization performance for both cases without introducing trade-offs, as shown in Fig. S15.

3.3.1 Conflict flows: periodic hills with different separation characteristics

The multi-objective framework automatically resolves competing objectives arising from two periodic hill cases with different separation characteristics, arising from differences in hill slope steepness controlled by the slope scaling factor α_{p}. The $\alpha_{\mathrm{p}} = 0.5$ case is characterized by massive separation,

with the recirculation region extending upstream of the hill crest, whereas the $\alpha_\mathrm{p} = 1.0$ case exhibits only mild separation confined to the leeward foot (see Fig. S13a).

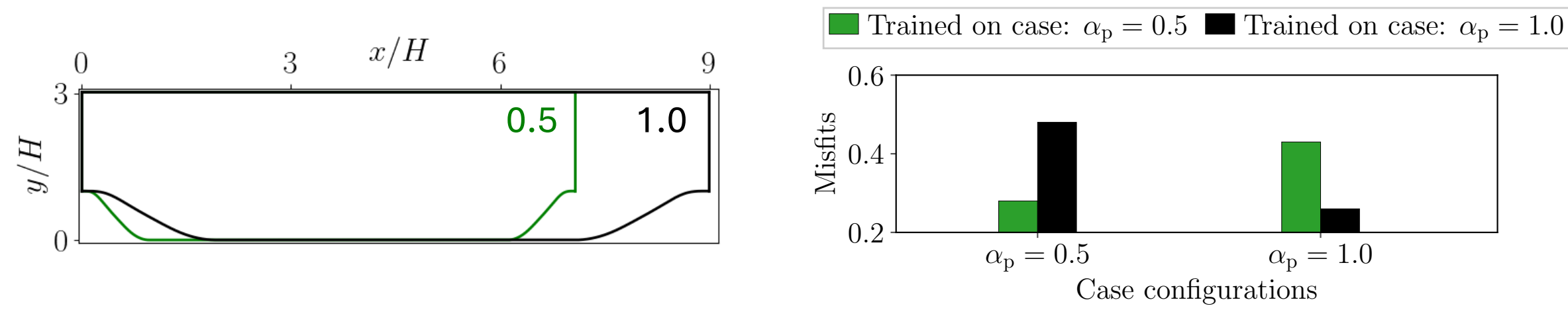

(a) Geometries

(b) Misfit results

Figure S13: Comparison of periodic hill geometries and single-case training misfit results. (a) Geometries of the two periodic hill configurations with slope parameters $\alpha_\mathrm{p} = 0.5$ and $\alpha_\mathrm{p} = 1.0$. (b) Misfit values obtained from single-case training, where models are trained on one configuration ($\alpha_\mathrm{p} = 0.5$ or $\alpha_\mathrm{p} = 1.0$) and evaluated on the other configuration.

Models trained on a single case fail to generalize across regimes. In particular, a model trained only on the mildly separated case ($\alpha_\mathrm{p} = 1.0$) incurs large errors when applied to the strongly separated case ($\alpha_\mathrm{p} = 0.5$), as quantified by the normalized misfits in Fig. S13b. This behavior is consistent with earlier findings that models trained on mildly separated periodic hill flows struggle to extrapolate to strongly separated configurations [19, 52]. These results illustrate the conflicting nature of the two objectives and motivate the use of a principled multi-task optimization strategy.

Fixed-weight loss aggregation cannot reliably reconcile the competing objectives because the optimal balance between cases is unknown a priori and requires manual tuning. To overcome this limitation, the Frank–Wolfe algorithm is employed to adaptively determine the weights by minimizing the norm of the weighted sum of the objective gradients. Two training strategies are compared: grid search over fixed-weight combinations and adaptive weighting using the Frank–Wolfe algorithm.

Adaptive weighting yields a characteristic training evolution in which the normalized misfits evolve jointly, as shown in Fig. S14a. At early stages, improvements in one case degrade the performance of the other, indicating strong competition between objectives. As training progresses, the adaptive weighting reconciles this conflict and drives the optimization toward a balanced solution, in which both cases reach normalized misfits of approximately 0.5. Fixed-weight optimization yields an uneven tradeoff that often improves one periodic hill case at the expense of the other in Fig. S14b. Grid search over fixed weights explores only a limited set of combinations and frequently produces models that favor one case while degrading the other, with several outcomes performing worse than the baseline. In contrast, the Frank–Wolfe–based adaptive strategy automatically traces a smooth tradeoff curve and converges toward a region in which both normalized misfits are simultaneously reduced, as shown in Fig. S14b.

3.3.2 Non-conflict flows: square duct and airfoil flows

The proposed framework preserves optimization performance in the non-conflict scenario because the training objectives are compatible. The training cases include a square duct flow and a high–angle-of-attack S809 airfoil flow, which impose distinct constraints on the turbulence model. The square duct case primarily requires accurate modeling of turbulence-induced secondary flows and mainly activates the quadratic term $g^{(2)}$, which governs anisotropy-driven secondary motions. In contrast, the S809 airfoil case focuses on capturing separation under strong adverse pressure gradients and predominantly constrains the linear term $g^{(1)}$, which controls separation behavior. Because each case

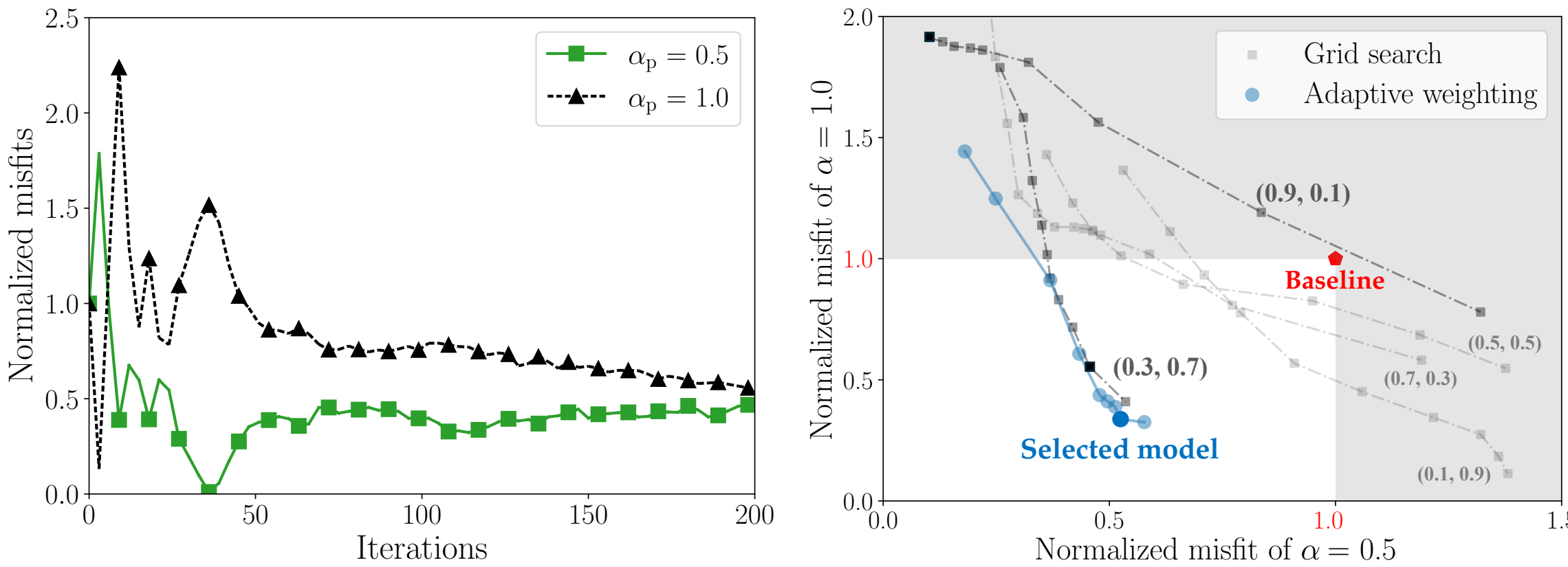

(a) Training evolution of normalized misfits

(b) Trade-off between normalized misfits

Figure S14: Training behavior and trade-off analysis for two periodic hill cases with different slope parameters. (a) Evolution of normalized misfits during training for the two cases with $\alpha_p = 0.5$ and $\alpha_p = 1.0$. (b) Tradeoff between the two normalized misfits for models obtained using grid search and adaptive weighting. Five symmetric weight pairs spanning 0.1–0.9 are selected and only iterations 150–200 are shown for clarity. The baseline k–ω model (⬟) is located at (1.0, 1.0).

emphasizes a different model component, their optimization directions remain aligned rather than conflicting.

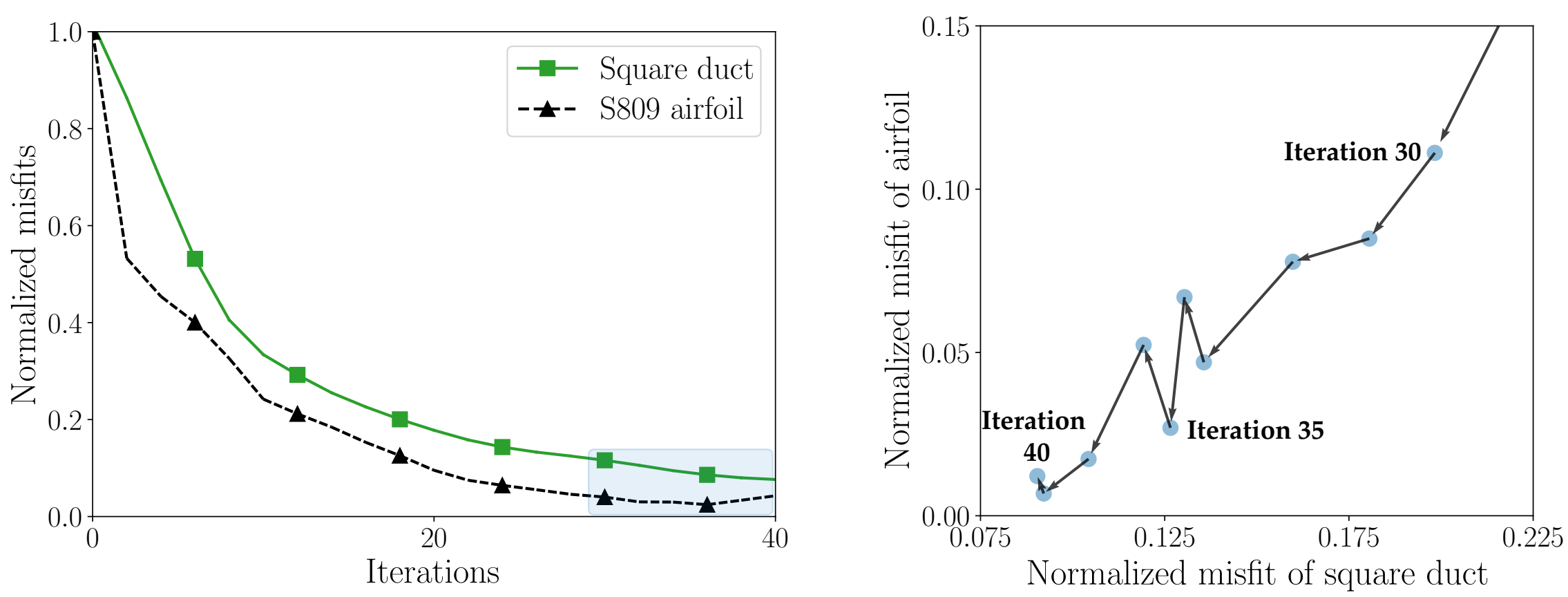

(a) Training evolution of normalized misfit

(b) Trade-off between normalized misfit

Figure S15: Training behavior and misfit evolution for the square duct and S809 airfoil cases. (a) Evolution of normalized misfits during training for the square duct case and the S809 airfoil case using the Frank–Wolfe algorithm. (b) Tradeoff of the normalized misfit values between two non-conflict cases obtained through Frank–Wolfe–based training, with selected iterations highlighted.

The optimization results confirm the absence of conflict between the objectives. As shown in Fig. S15a, the algorithm simultaneously reduces the misfits for both cases during training, indicating stable and consistent convergence. No pronounced Pareto front is observed in Fig. S15b, indicating that no meaningful trade-off exists between the objectives. This behavior confirms that, in the non-conflict scenario, reweighting the objectives has negligible influence on the optimal solution.

Flows	4 objectives	8 objectives	12 objectives	16 objectives
NACA 0012 airfoil (AOA = 18°, drag & lift)				✓
S809 airfoil (AOA = 14°, drag & lift)		✓	✓	✓
Square duct (Re = 1100)		✓	✓	✓
Square duct (Re = 3500)	✓	✓	✓	✓
Rectangular duct (AR = 5)		✓	✓	✓
Rectangular duct (AR = 10)				✓
Channel (Re = 3300)				✓
Round jet	✓	✓	✓	✓
Hump	✓	✓	✓	✓
Curved step			✓	✓
Bump (height = 31)			✓	✓
Periodic hills ($\alpha = 0.8$)			✓	✓
Periodic hills ($\alpha = 1$)	✓	✓	✓	✓
Periodic hills ($\alpha = 1.5$)			✓	✓

Table S2: Canonical flows used as training objectives for different objective set sizes (4, 8, 12, and 16). A checkmark indicates the inclusion of each flow. Detailed flow definitions are provided in Table S4.

3.3.3 Scalability evaluation for multi-objective learning

We assess the scalability of the proposed multi-objective ensemble learning framework by increasing the number of training objectives selected through the distribution-based case selection strategy. Models are trained with 4, 8, 12, and 16 objectives, with the corresponding flows summarized in Table S2 and the resulting normalized misfits shown in Fig. S16. For 4, 8, and 12 objectives, the optimized solutions consistently outperform the baseline k–ω model, indicating clear Pareto improvement across all objectives. Notably, some objectives achieve performance levels close to those obtained from singly trained models. When the number of objectives increases to 16, optimization becomes more challenging. Several objectives approach or slightly underperform the baseline, and most objectives exhibit more limited improvement, with only one objective reaching approximately half of its singly trained performance. These results indicate stronger trade-offs among competing objectives as the optimization problem becomes more complex, suggesting that additional training iterations or a more expressive model representation may be required to further enhance performance. Nevertheless, the framework maintains stable behavior overall, highlighting its robustness and scalability under highly multi-objective settings.

3.3.4 Sensitivity analysis on model size

We assess the sensitivity of the turbulence model to neural network size by varying the number of neurons per layer (width) in each sub-network, while keeping the overall architecture and depth fixed. This analysis focuses on the scenario with 8 objectives (see Table S2).

The study shows that the reference configuration provides the best balance between accuracy and robustness within a many-objective, ensemble-based training setting. Eight training objectives define a many-objective learning scenario, with an ensemble size of 20 samples chosen for computational efficiency. This setup establishes a practical regime for ensemble-based training, where the number of trainable parameters must remain compatible with the ensemble size. Within this regime, four model sizes are examined, ranging from a compact configuration to a heavily over-parameterized one

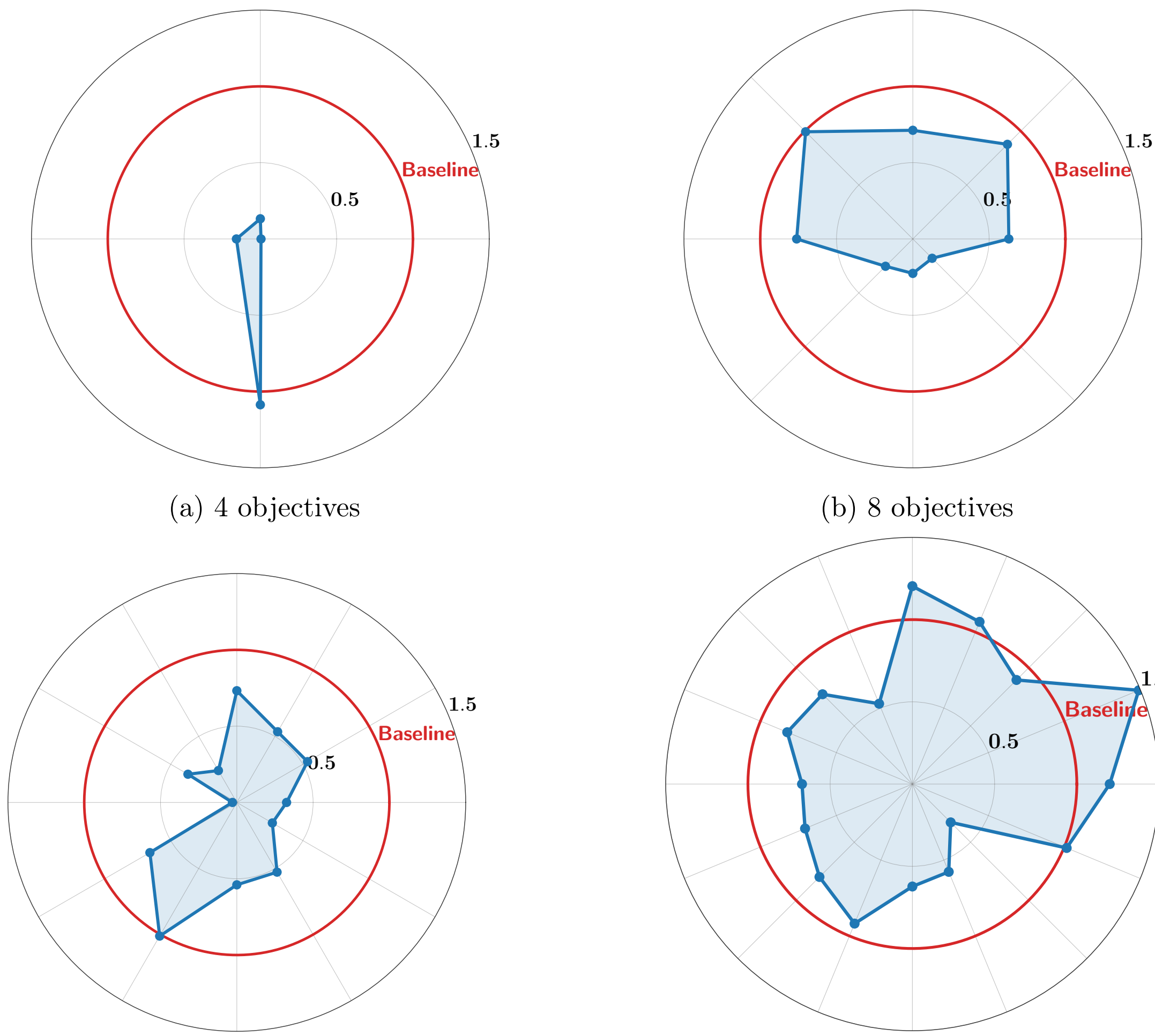

(a) 4 objectives (b) 8 objectives (c) 12 objectives (d) 16 objectives

Figure S16: Scalability of the multi-objective ensemble learning framework. The analysis compares models trained with 4, 8, 12, and 16 objectives selected through the distribution-based case selection strategy. Each objective (●) is evaluated by its normalized misfit, with the filled blue polygon representing the collective optimized solution. The baseline k–ω model performance is denoted by the red circle. The results show clear Pareto improvement for 4, 8, and 12 objectives, where all optimized outcomes lie well within the baseline boundary. When scaling to 16 objectives, improvements become less uniform, with some objectives approaching or slightly exceeding the baseline.

(see Table S3). The comparison (Fig. S17) shows that the reference configuration achieves a good balance between accuracy and robustness, the compact model exhibits slightly degraded performance due to limited representational capacity, and larger models do not yield consistent improvements, with performance degrading as model size increases.

The performance degradation observed for larger models arises from a mismatch between model size and ensemble size. In ensemble-based learning, parameter updates are confined to a low-rank subspace determined by the ensemble size [53]. Although modern localization techniques (e.g., noise-informed covariance estimation [54]) are employed, effective learning in high-dimensional parameter spaces remains limited by the ensemble size. As the number of parameters grows relative to the ensemble size, training becomes less efficient and less stable.

These results indicate that relatively small neural networks are sufficient to represent the underlying turbulence physics, consistent with the low-complexity functional forms of traditional turbulence models. For larger networks to be effective, the ensemble size must be increased accordingly.

Configuration	$g^{(1)}$ width	$g^{(2)}$ width	β width	Total parameters
Compact model	3	4	3	195
Reference model	4	6	4	333
Expanded model	6	9	6	651
Over-parameterized model	9	13	9	1275

Table S3: Neural network configurations used in the sensitivity analysis on model size. All models use 4 hidden layers. The model size is varied by changing the number of neurons per layer in each sub-network.

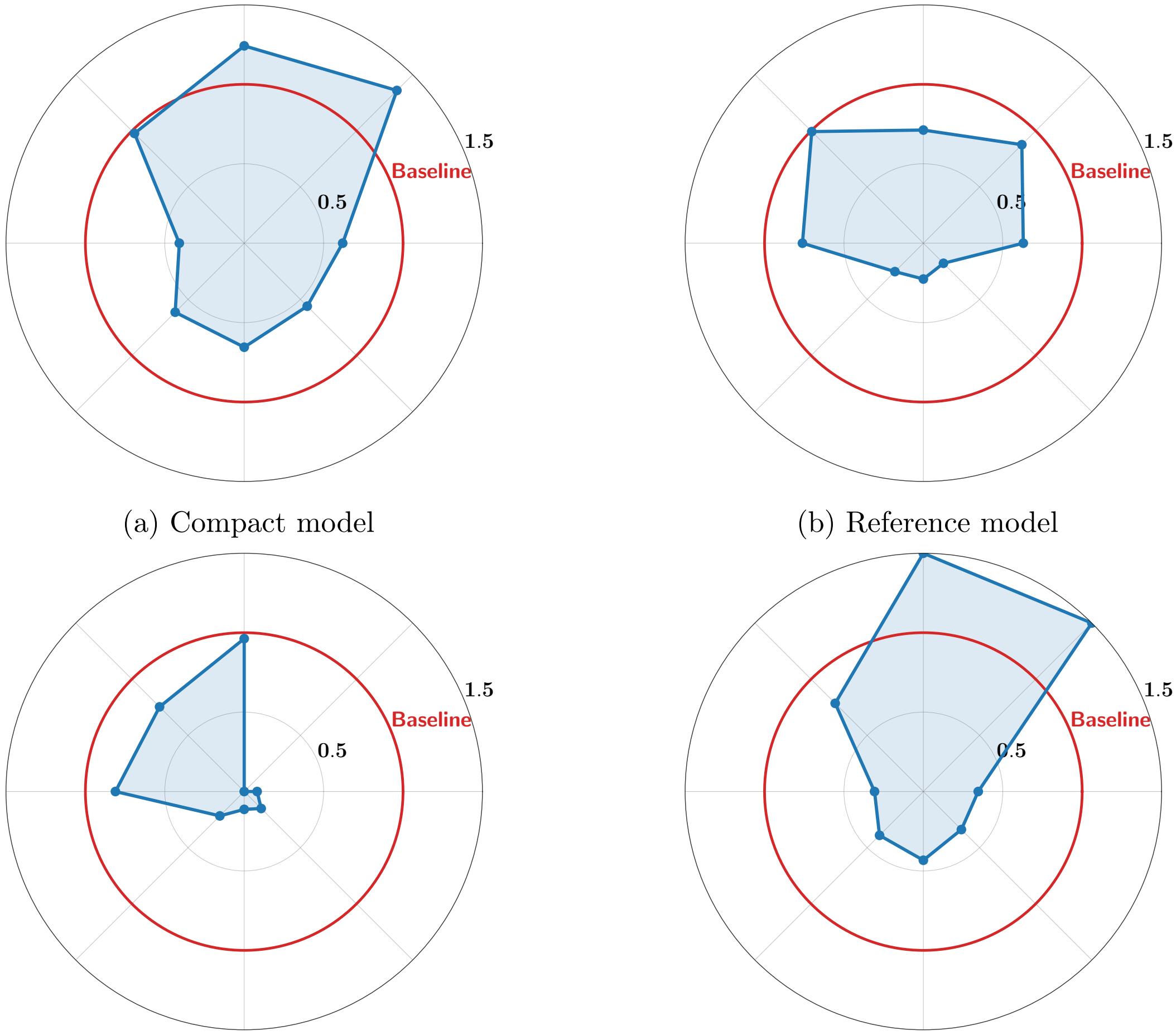

(a) Compact model

(b) Reference model

(c) Expanded model

(d) Over-parameterized model

Figure S17: Sensitivity of the turbulence model to neural network size, shown as radar plots for configurations with different numbers of trainable parameters relative to the reference model. The reference model (b) contains 333 parameters. The other configurations are a compact model (a) with 195 parameters, a moderately expanded model (c) with 651 parameters, and an over-parameterized model (d) with 1275 parameters. Model size is varied by adjusting the number of neurons per layer (width) in each sub-network while keeping the overall architecture unchanged. The radial coordinate represents the normalized misfit $\hat{e}$ for each objective. The red circle denotes the baseline model, and the blue polygon represents the learned model.

4 Setup of training and test cases

We present the training and test cases used to evaluate three turbulence models: the unified foundation model, the specialist model for separated flows, and the specialist model for secondary flows with separation. The setup of all cases together with the corresponding observation data are summarized in Table S4.

Categories	Flows	Observations	No. obs	Control parameter	Value	Label
Attached Boundary Layers	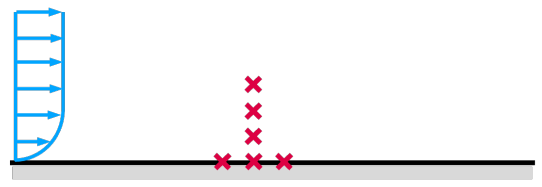<br>Zero pressure gradient flat plate	Velocities, friction	34	Reynolds number	5×10^6	BL1
	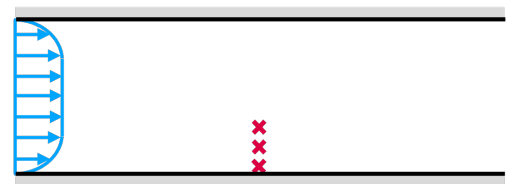<br>Plane channel	Velocities	4	Reynolds number	3300<br>7890<br>8×10^7	BL2.1<br>BL2.2<br>BL2.3
	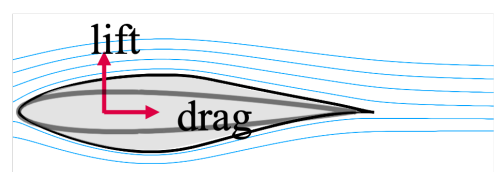<br><br>Airfoils (low AOA)	Lift, drag	2	Angle of attack	S809: 1°, 5°<br>NACA 0012: 10°, 15°	BL3.1, 3.2<br>BL3.3, 3.4
Separated Flows	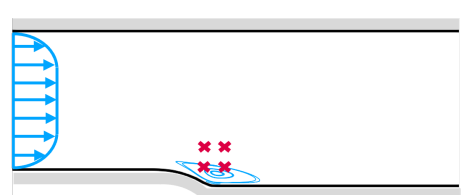<br>Curved step	Velocities	27	Reynolds number	13700	SP1
	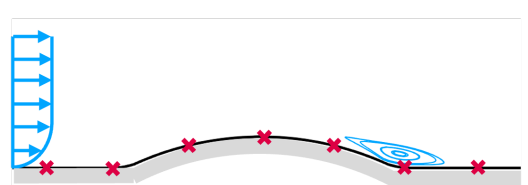<br>Bump	Friction	42	Height	20 mm<br>26 mm<br>31 mm<br>38 mm<br>42 mm	SP2.1<br>SP2.2<br>SP2.3<br>SP2.4<br>SP2.5
	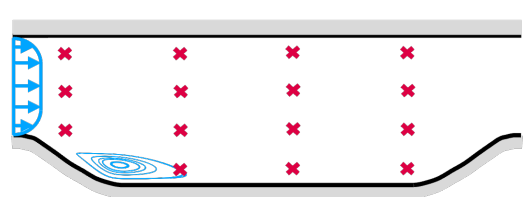<br>Periodic hill	Velocities	66	Slope steepness	0.8<br>1.0<br>1.2<br>1.5	SP3.1<br>SP3.2<br>SP3.3<br>SP3.4
	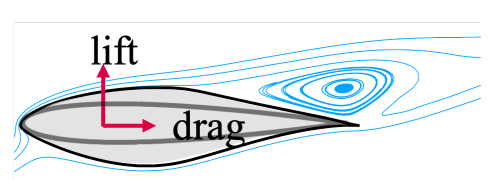<br><br>Airfoils (high AOA)	Lift, drag	2	Angle of attack	S809: 14°, 16°, 18°<br>NACA 0012: 18°	SP4.1–4.3<br>SP4.4
	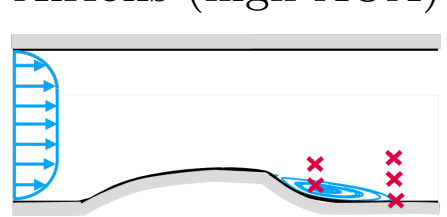<br>Hump	Velocities	200	Reynolds number	936000	SP5
Secondary Flows (Ducts)	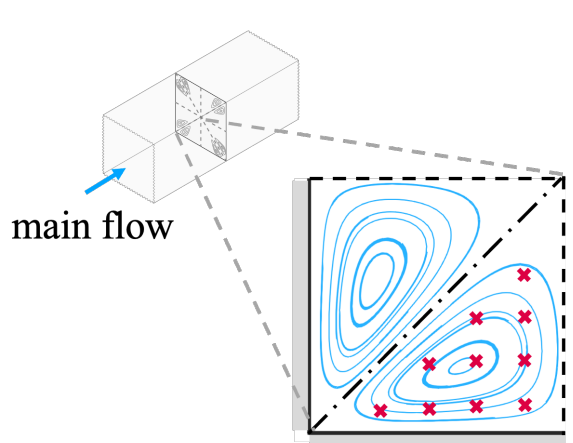<br><br>Square duct	Velocities	193	Reynolds number	1100<br>1800<br>2600<br>3500	DU1.1<br>DU1.2<br>DU1.3<br>DU1.4
	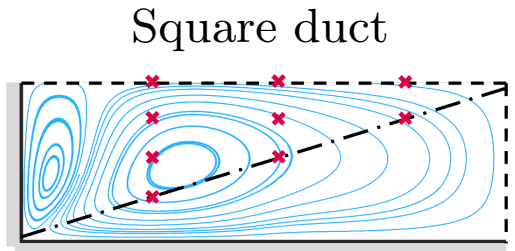<br>Rectangular duct	Velocities	35	Aspect ratio	3<br>5<br>7<br>10	DU2.1<br>DU2.2<br>DU2.3<br>DU2.4

Continued on next page

Table S4 continued from previous page

Categories	Flows	Observations	No. obs	Control parameter	Value	Label
Free-shear Flow	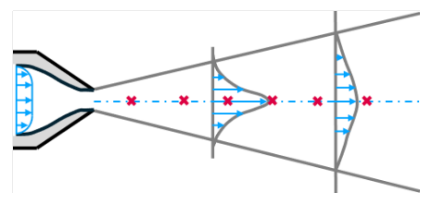 Round jet	Velocities	25	Reynolds number	5601	FS1
Complex 3D Flows	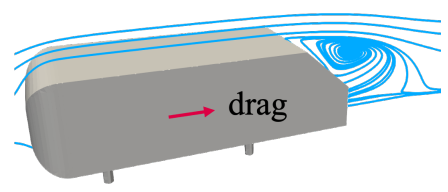 Generic car (Ahmed body)	Drag	-	Slant angle	40°	CF1
	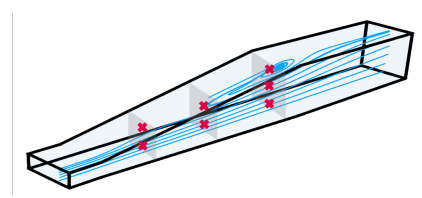 3D diffuser	Velocities, friction	-	Reynolds number	10000	CF2
	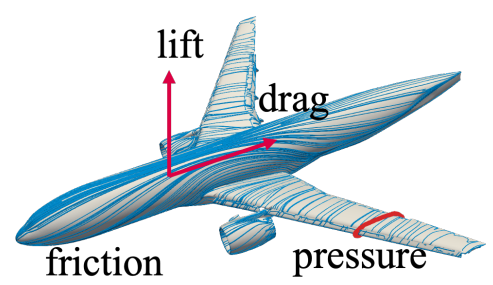 Generic aircraft (CRM-HL)	Lift, drag, friction, pressure	-	Angle of attack	7.05°<br>21.47°	CF3<br>CF4
					**Totals**	**36**

Table S4: Overview of flow cases and associated observation data used in the present study. The table summarizes the flow categories, configurations, observed quantities (× denotes sparse observations), number of observations, control parameters, parameter values, and case labels. Attached boundary-layer flows include a flat plate [55], plane channels [39], and S809 [56] and NACA 0012 [57] airfoils at low angles of attack. Separated flows include a curved step [58], bumps [59], a hump [60], S809 and NACA 0012 airfoils at high angles of attack, and periodic hills [40]. Secondary flows include square ducts [61] and rectangular ducts [62]. Free-shear flow includes a round jet [63], and complex 3D flows include a generic car (Ahmed body) [64], a 3D diffuser [65] and a generic aircraft (NASA High-Lift Common Research Model, referred to as CRM-HL) [66].

4.1 Training cases for unified foundation model

Training cases for the unified foundation model are selected using a distribution-based strategy that balances flow-mechanism diversity, representativeness, and training cost. To this end, flow cases are embedded into a two-dimensional distance-preserving space based on flow-feature distribution distances and grouped into six clusters, while attached boundary layers are excluded from training and reserved for verification. As shown in Fig. S18b, cases belonging to the same cluster share similar flow characteristics, and the highlighted markers indicate the selected training cases. Representative cases are then selected to cover distinct physical behaviors (see Fig. S19). For secondary flows, three cases are selected from two clusters to represent both rectangular and square duct geometries at different Reynolds numbers. For separated flows, three cases are manually chosen from one cluster to capture distinct pressure-gradient effects, including internal and external separation under adverse pressure gradients (periodic hill and curved step) and separation with relaminarization under a favorable pressure gradient (bump). Additional separated flow cases, including the hump and a high-angle-of-attack S809 airfoil, are selected from two other clusters. A free-shear flow is represented by a round jet from the remaining cluster. In total, nine representative flow cases are selected for training. For the S809 airfoil, lift and drag are treated as separate, conflicting objectives, yielding ten training objectives in the multi-objective learning framework. The unified foundation model trained on these cases is

evaluated across flows within the training categories, complex three-dimensional flows, and attached boundary layers as an additional fundamental flow category (Fig. S3).

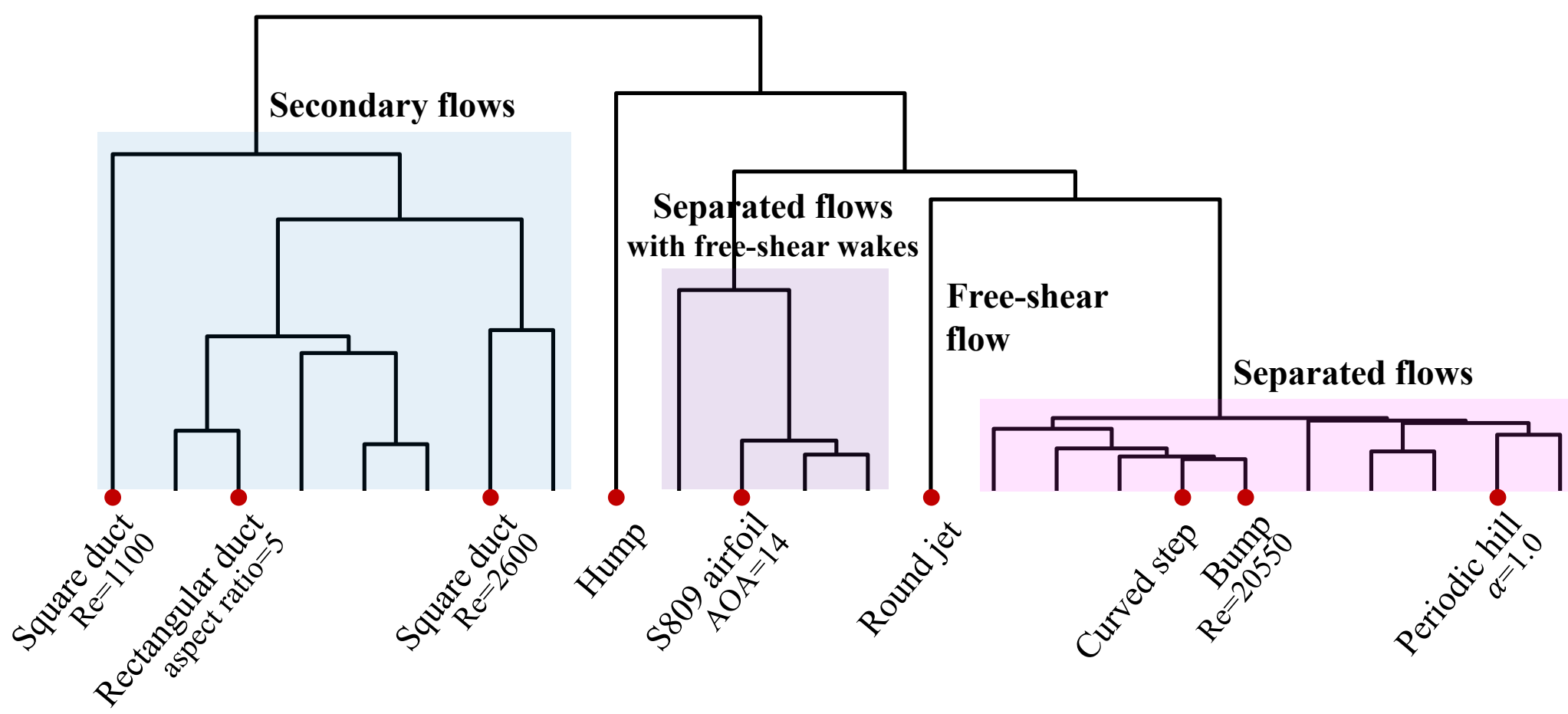

(a) Hierarchical clustering

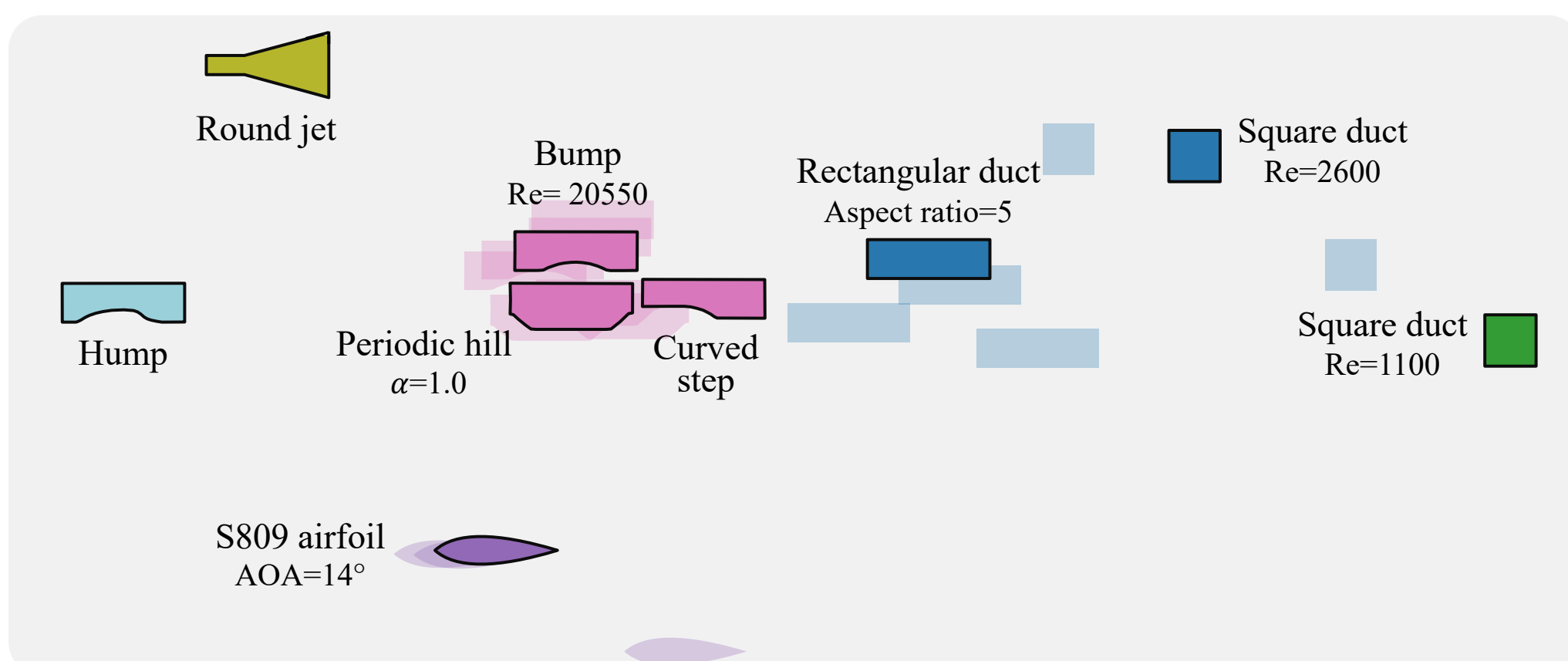

(b) 2D Distance-Preserving Embedding Space

Figure S18: Distribution-based selection of training cases for the **unified foundation model**. Flow cases (excluding attached boundary layers reserved for verification) are compared using flow-feature distribution distances to identify representative training cases that span distinct flow mechanisms. (a) Hierarchical clustering dendrogram constructed from pairwise Wasserstein distances between flow-feature distributions, revealing clear groupings corresponding to secondary flows, separated flows, and free-shear flows. (b) Two-dimensional distance-preserving embedding obtained via multi-dimensional scaling, which visualizes the clustering structure and highlights relative similarities among flow cases. Representative cases are selected to cover key mechanisms and are highlighted with solid lines and opaque colors, including rectangular and square ducts for secondary flows, the S809 airfoil, periodic hill, bump, hump, and curved step for separated flows, and the round jet for free-shear flow. The bump case is labeled by its Reynolds number (Re= 20550), corresponding to a bump height of 31 mm. In total, nine flow cases are selected; for the S809 airfoil, lift and drag are treated as separate objectives, resulting in ten training objectives.

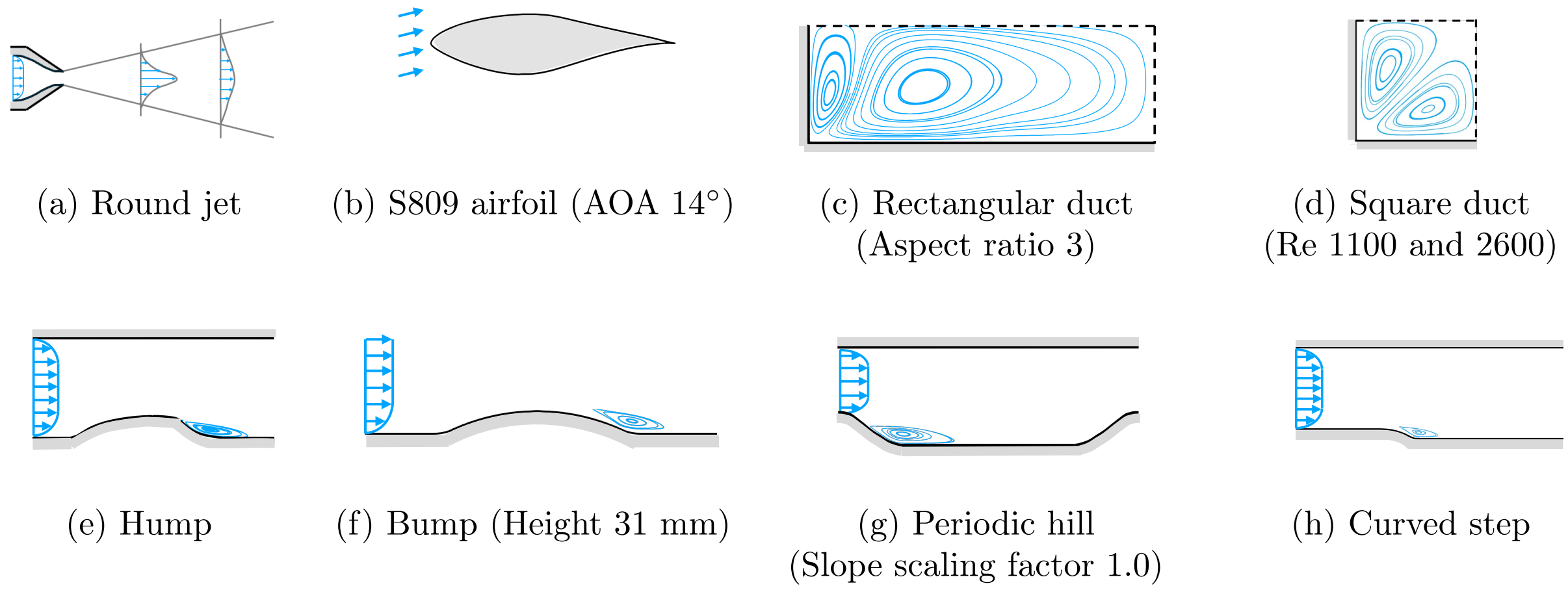

(a) Round jet
(b) S809 airfoil (AOA 14°)
(c) Rectangular duct (Aspect ratio 3)
(d) Square duct (Re 1100 and 2600)
(e) Hump
(f) Bump (Height 31 mm)
(g) Periodic hill (Slope scaling factor 1.0)
(h) Curved step

Figure S19: Training configurations used for the **unified foundation model**, selected to span a diverse set of flow mechanisms. The training set comprises nine representative cases, including free-shear flow (round jet), external separated flows (S809 airfoil at AOA = 14°, periodic hill with slope scaling factor $\alpha_\mathrm{p} = 1.0$ and bump with height 31 mm), secondary flows in ducts (rectangular duct with AR = 3 and square ducts at Re = 1100 and 2600), and internal separated flows (hump and curved step).

External separated flows Flow over the **S809 airfoil** at high angles of attack (AOA > 12°) is dominated by massive flow separation, making it challenging for traditional turbulence models, which often overestimate the lift force beyond stall [67]. At $\mathrm{Re} = 2 \times 10^6$ based on chord length and inflow velocity, the suction side experiences a strong adverse pressure gradient, causing early separation. The case at AOA = 14° is selected for training, using experimentally measured lift and drag coefficients as observations. Flow over a **bump** is a widely used two-dimensional test case for turbulence models because the curved surface first accelerates the flow (favourable pressure gradient) and then decelerates it (adverse pressure gradient), strongly disturbing the boundary layer and, for sufficiently large bumps, bringing it close to separation. The configuration consists of a wall-mounted circular arc smoothly blended into a flat plate by convex fillets at both ends. At the selected operating condition, the inflow Reynolds number based on the momentum thickness and freestream velocity is $\mathrm{Re}_{\theta_\infty} = 2500$. The imposed curvature generates a strong adverse pressure gradient downstream of the crest, which causes primary separation near the trailing fillet and forms a short recirculation bubble. The inlet velocity and turbulence profiles are obtained from a precursor large-eddy simulation of a zero-pressure-gradient turbulent boundary layer at the same $\mathrm{Re}_{\theta_\infty}$. Zero-gradient conditions are applied at the top and outlet boundaries, and the lower boundary is treated as a no-slip wall. The training observations consist of the wall skin-friction coefficient c_f sampled at 42 streamwise locations from the large-eddy simulation results.

Internal separated flows Flow over **periodic hill** is a benchmark for internal separated flows, characterized by turbulence in the presence of periodic smooth hills that induce flow separation and reattachment. The setup operates at a Reynolds number $\mathrm{Re}_{h_\mathrm{p}} = 10595$, based on the bulk velocity and hill height h_p, with a slope scaling factor $\alpha_\mathrm{p} = 1.0$. Smaller values of α_p correspond to steeper hills, while larger values indicate gentler slopes. The domain applies streamwise-periodic boundary conditions, no-slip walls, and a specified bulk velocity. Velocities from DNS data are extracted at four cross-sections ($x/h_\mathrm{p} = 1.0, 3.0, 5.0, 7.0$), yielding 66 velocity components as observations. The **curved step** case evaluates turbulence model performance for separation caused by abrupt streamline curvature acting on a fully developed turbulent boundary layer. The flow consists of an upstream convex ramp that terminates in a sudden downward step, which forces detachment without prior boundary-layer acceleration. Unlike periodic-hill and bump flows, where separation follows an accelerating boundary layer, this configuration isolates curvature-induced separation physics. The

setup resolves both the attached upstream boundary layer and the separated region downstream of the step. The flow operates at Re = 13700, and the training data comprise 27 velocity measurements sampled along several wall-normal lines. The **hump** case serves as a validation benchmark to assess a turbulence model's ability to reproduce adverse–pressure-gradient–induced separation, reattachment, and boundary-layer recovery in a nominally two-dimensional flow. The flow is fully turbulent upstream, with a freestream velocity of approximately 34.6 m/s and a Reynolds number of $\mathrm{Re} = 9.36 \times 10^5$ based on the hump chord $c = 420$ mm. The observation data consist of velocity measurements at three distinct streamwise locations along the hump surface. This configuration has proven challenging for RANS models, which typically underpredict turbulent shear stress in the separated shear layer and consequently overpredict the separation length and delay reattachment.

Secondary flows Two **square duct** flows and one **rectangular duct** flow are included as training cases to expose the model to turbulence-induced anisotropy across different Reynolds numbers and geometric variations. Square duct flow produces secondary motions driven by anisotropy in a non-circular cross-section, and the simulations assume fully developed conditions with periodic streamwise boundaries and no-slip walls. The computations reduce cost by modeling one-quarter of the cross-section using symmetry. Two square-duct cases at different Reynolds numbers (Re = 1100, 2600) isolate Reynolds-number effects on secondary-flow intensity and anisotropy. The rectangular duct case, with an aspect ratio of 5, introduces a strong geometric asymmetry that alters the structure, relative strength, and spatial distribution of the secondary vortices while preserving the same underlying mechanism. The training data include mean velocities in all three directions at four streamwise locations, totaling around 200 velocity components for each case.

Free-shear flows The **round jet** evolves from a potential core into a turbulent shear layer before mixing with the ambient fluid. The total pressure is imposed at the jet inlet, pressure-outlet conditions are applied at the outlet, velocity is specified for the freestream inflow, and no-slip conditions are applied on the walls. Model validation requires reproducing the centerline velocity decay. Accordingly, we used 25 streamwise velocities along the centerline as training observations.

4.2 Test cases for unified foundation model

We evaluate the unified foundation model on 27 turbulent flows, divided into three groups: flows within the training categories, complex three-dimensional flows, and one additional fundamental flow category: the attached boundary layer (Table S5). The additional flow category constitutes an asymptotic consistency check for compliance with the law of the wall.

Attached boundary layers The attached boundary layer category contains eight test cases: a flat plate, channel flows at three Reynolds numbers, S809 airfoils at two low angles of attack and NACA 0012 airfoils also at two low angles of attack. The flat plate case employs uniform inflow and no-slip boundary conditions at the wall, with periodicity applied in the spanwise direction. Two velocity values are sampled along the wall-normal direction to capture near-wall behavior, and the case represents the canonical development of a turbulent boundary layer, where viscous effects dominate close to the wall and gradually transition to turbulent momentum transport farther away. The channel flows at three different Reynolds numbers ($\mathrm{Re} = 3300, 7890, 8 \times 10^7$) use periodic streamwise boundaries and no-slip walls, exhibit the classical viscous, buffer, and logarithmic layers, and use four streamwise velocity samples along the wall-normal direction to probe Reynolds-number effects under otherwise identical conditions. The attached S809 airfoil test cases at $\mathrm{AOA} = 1^\circ$ and 5° follow the same setup as the training cases, differing only in angle of attack. Lift and drag coefficients are calculated over the entire airfoil surface. These low angles maintain attached flow without massive separation, ensuring they remain representative of attached boundary layers. NACA 0012 airfoil at $\mathrm{AOA} = 10^\circ$ and 15° are also included here. Despite the higher angles of attack, these configurations remain predominantly attached at the selected Reynolds number and therefore also represent attached boundary layers. Their inclusion extends the evaluation to a different airfoil geometry and a higher loading regime while preserving the same underlying flow category.

**Case**	**Control parameter**	**Value**	**No. of cases**
Flat plate	Reynolds number	5×10^6	1
Plane channel	Reynolds number	3300<br>7890<br>8×10^7	3
S809 airfoil	Angle of attack	1°<br>5°<br>16°<br>18°	4
NACA 0012 airfoil	Angle of attack	10°<br>15°<br>18°	3
Periodic hill	Slope scaling factor	0.8<br>1.2<br>1.5	3
Bump	Height	20 mm<br>26 mm<br>38 mm<br>42 mm	4
Square duct	Reynolds number	1800<br>3500	2
Rectangular duct	Aspect ratio	3<br>7<br>10	3
Generic car (Ahmed body)	Slant angle	40°	1
3D diffuser	Reynolds number	10^4	1
Generic aircraft (CRM-HL)	Angle of attack	7.05°<br>21.47°	2
		**Totals**	**27**

Table S5: Summary of **test cases** used to evaluate the **unified foundation model**, including flow configuration, control parameter, parameter values, and the number of cases for each configuration, for a total of 27 test cases.

Separated flows The separated flow category contains four types of test cases: bump with different heights, S809 airfoil at two high angles of attack, periodic hills at three slope scaling factors and NACA 0012

airfoil also at one high angle of attack. The bump cases with different heights are simulated with the similar setup as the training case, with the wall friction coefficient used to assess the predictions of separation. The S809 airfoil test cases at AOA = 16° and 18° share the same setup and sampling strategy as the training case, with lift and drag coefficients extracted; at these higher angles, separation and stall dominate the flow behavior. The NACA 0012 airfoil case at AOA = 18° is likewise included as a separated-flow test case. At this angle, the flow exhibits strong separation over a large portion of the suction side, placing it firmly in the stalled regime and making it representative of highly non-equilibrium boundary-layer behavior. The periodic hill cases at three slope scaling factors ($\alpha_\mathrm{p} = 0.8$, 1.2, 1.5) impose periodic streamwise and spanwise boundaries with no-slip walls at top and bottom, and streamwise velocity is sampled along wall-normal lines downstream of the hill crest, with points taken every five computational cells, to determine separation and reattachment lengths. Together, these cases form a diverse testbed of separated turbulent flows, ranging from localized recirculation to fully stalled aerodynamic configurations.

Secondary flows The secondary flow category contains two types of test cases: square ducts at two Reynolds numbers (Re = 1800, 3500) and rectangular ducts at three aspect ratios ($\mathrm{AR} = 3, 7, 10$). In both geometries, a fully developed turbulent profile is prescribed at the inlet, with no-slip walls along all duct boundaries and a constant mass flux condition to sustain the flow. The domain is periodic in the streamwise direction, ensuring statistical stationarity. Velocity observations are taken in cross-sectional planes, identical to the sampling strategy used in the training case, to capture mean velocity distortion and secondary vortex development. The square duct flows emphasize corner-induced vortices, while the rectangular duct flows show how aspect ratio affects secondary circulations. Together, these cases test the ability of turbulence models to capture inherently secondary motions in duct flows.

Complex 3D flows The generic car challenges turbulence models with bluff-body wake dynamics, including shear-layer vortices, corner vortices, and a large base recirculation zone. Observation data consist of the surface-integrated drag coefficient, which reflects the accuracy of predicted separation and wake structures. The asymmetric 3D diffuser tests turbulence models under adverse pressure gradients and Reynolds stress anisotropy that drive asymmetric separation. Observation data include velocity profiles across the separated shear layer, and wall friction trends that capture onset and reattachment.

The generic aircraft configuration, based on the high-lift Common Research Model, represents a realistic transport aircraft with deployed slat, wing, and flap elements and is studied under high-Reynolds-number conditions ($M = 0.2$, $\mathrm{Re} = 5.49 \times 10^6$). It generates complex three-dimensional flow characterized by interactions among boundary layers, wakes, and coherent vortical structures, leading to strong Reynolds stress anisotropy and vortex–boundary-layer–driven separation. Two representative conditions are considered: a pre-stall case at AoA = 7.05°, corresponding to mildly separated flow before stall and a post-stall case at AoA = 21.47°, where stall occurs and large-scale flow separation dominates. Experimental and large-eddy simulation datasets provide integrated forces (lift and drag) and surface measurements (pressure and skin friction).

4.3 Specialist turbulence model for separated flows

Conventional turbulence models often exhibit poor performance in flows with massive separation. To address this limitation, we fine-tune a specialist model tailored for separated flows. This section describes the training configurations and the test cases selected to evaluate its predictive performance.

Training cases To obtain a specialist model for flows with complex separation, we fine-tune the model using two benchmark separated flows: flow over a bump with 42 mm height and flow over a curved step as shown in Fig. S20. The bump's convex curvature induces an adverse pressure gradient that produces an incipient separation bubble with reattachment several bump heights downstream, while the curved step's sudden streamline curvature generates a large separation region followed by gradual reattachment along the lower wall.

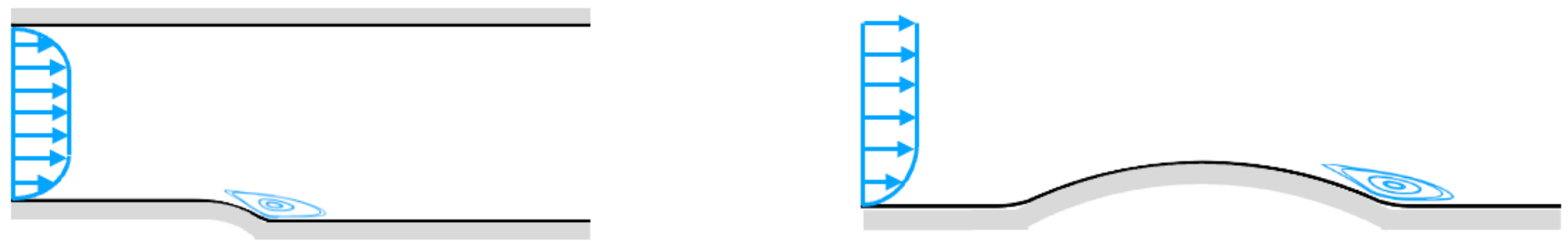

(a) Curved step (b) Bump (height = 42 mm)

Figure S20: **Training configurations** used for the **specialist model for separated flows**. The training set consists of two benchmark configurations, including a curved-step flow and a bump flow with height 42 mm.

Test cases Two groups of cases for evaluating the trained specialist model for separated flows are chosen and summarized in Table S6, namely test cases within the training categories: bumps with different heights $(20, 26, 31, 38\,\text{mm})$ and complex three-dimensional case: the generic car shown in Table S4.

**Case**	**Control parameter**	**Value**	**No. of cases**
Bump	Height	20 mm<br>26 mm<br>31 mm<br>38 mm	4
Generic car	Slant angle	40°	1
		**Totals**	**5**

Table S6: **Test cases** used to evaluate the **specialist model for separated flows**, including flow configuration, control parameter, parameter values, and the number of cases for each configuration, for a total of 5 test cases.

The bumps retain the same configuration as in training while varying the bump height, which systematically alters the adverse pressure gradient and separation strength. These cases extend the training set into controlled parametric variations, enabling assessment of model robustness.

The generic car is a standard benchmark in automotive aerodynamics, used to evaluate turbulence models for bluff-body flows. The model is placed in a wind tunnel domain and a uniform inlet yields a Reynolds number $\text{Re} = 2.8 \times 10^6$. A zero-pressure outlet boundary condition is applied at the downstream end, no-slip conditions are imposed on all solid surfaces, and symmetry conditions are used on the top and lateral boundaries to exploit geometric symmetry and reduce computational cost. The drag coefficient is calculated over the entire generic car surface as the observation data. The 40° slanted rear surface produces a complex three-dimensional wake, including top-shear-layer vortices, corner vortices, and a large base recirculation bubble. These flow structures make the generic car a stringent test for assessing turbulence model capability in predicting complex three-dimensional separation and wake dynamics.

4.4 Specialist turbulence model for secondary flows with separation

We fine tune a specialist turbulence model aimed at accurately capturing secondary flows with separation (multiple mechanisms). Recognizing that practical turbulent flows rarely involve a single dominant mechanism, but instead arise from complex interactions among multiple phenomena, the model is trained on configurations where both effects are prominent. This section outlines the training setups and test cases used to evaluate the model's predictive performance.

Training cases We select three benchmark cases to fine tune the specialist model for secondary flows with separation shown in Fig. S21, targeting large flow separation and secondary flow mechanisms: flow over a curved step and flows through two rectangular ducts with aspect ratio AR = 3, 10. These duct cases are detailed in §4.1. The rectangular duct flow exhibits asymmetric corner vortices, with the vortex on the horizontal wall expanding spanwise and dominating while the vertical wall vortex is compressed, producing a skewed concave flow pattern unlike the symmetric structure in square ducts. Training on these cases enhances the model's ability to capture secondary flows, large separations, and their interactions.

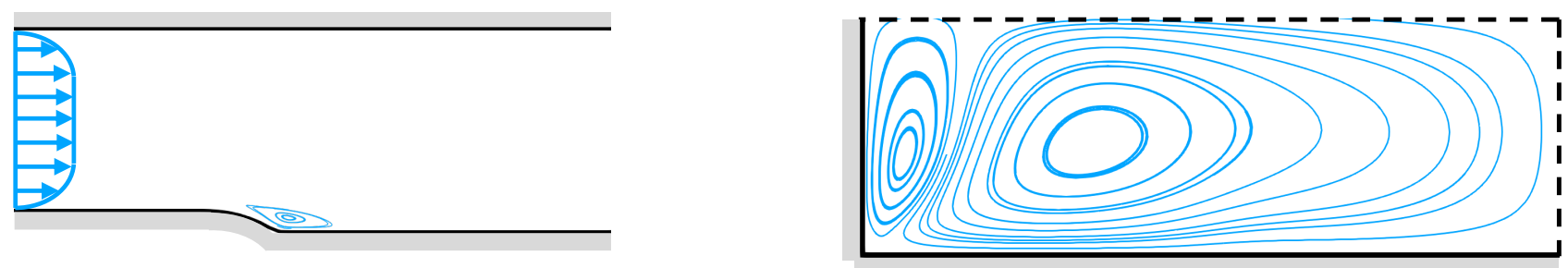

(a) Curved step (b) Rectangular ducts (Aspect ratio 3 and 10)

Figure S21: **Training configurations** used for the **specialist model for secondary flows with separation**. The training set consists of three benchmark configurations, including a curved-step flow and rectangular-duct flows with aspect ratios AR = 3 and 10.

Test cases We select three groups to evaluate the trained specialist model for secondary flows with separation shown in Table S7: (i) secondary flow cases: square ducts at Re $= 1100, 1800, 2600, 3500$; rectangular ducts with aspect ratio 5 and 7; (ii) separated cases: bumps with heights $20, 26, 31, 38, 42$ mm; hump and periodic hills with slope scaling factor $\alpha_{\mathrm{p}} = 0.8, 1.0, 1.2, 1.5$; (iii) a complex three-dimensional diffuser case. All the test cases within the training categories are detailed in Section. 4.2 and we now focus on the 3D diffuser.

The 3D diffuser [65] is a challenging validation case for turbulence models. It features incompressible, asymmetric internal flow with strong adverse pressure gradients and significant Reynolds stress anisotropy. The resulting complex three-dimensional separation closely mirrors the flow behavior in practical diffusers, such as those found between compressors and combustors in jet engines. This case is therefore a valuable benchmark for evaluating a model's ability to predict corner separation and secondary flow dynamics. The diffuser consists of an inlet channel, a diffuser section, and a straight outlet section. Both the upper wall and the sidewall expand. Boundary conditions prescribe a fully developed turbulent inflow with a bulk velocity $U_{\mathrm{b}} = 1$ m/s, leading to a Reynolds number Re $= 10^4$ based on inlet channel height. The diffuser test case is designed to capture three-dimensional separation and reattachment driven by wall expansion. Velocities are sampled at multiple wall-normal locations downstream of the expansion, as indicated by the red markers in Table S4, to resolve the separated shear layer and recirculation bubble. In addition to velocity profiles, the wall friction coefficient is monitored along the bottom wall to quantify separation onset and reattachment. In total, 64 observations are used, providing detailed information on both the mean flow field and wall-bounded response.

**Flow**	**Control parameter**	**Value**	**No. of cases**
Square duct	Reynolds number	1100<br>1800<br>2600<br>3500	4
Rectangular duct	Aspect ratio	5<br>7	2
Bump	Height	20 mm<br>26 mm<br>31 mm<br>38 mm<br>42 mm	5
Hump	Reynolds number	9.36×10^5	1
Periodic hill	Slope scaling factor	0.8<br>1.0<br>1.2<br>1.5	4
3D diffuser	Reynolds number	10^4	1
		**Totals**	**17**

Table S7: **Test cases** used to evaluate the **specialist model of secondary flows with separation**, including flow configuration, control parameter, parameter values, and the number of cases for each configuration, for a total of 17 test cases.

5 Results

5.1 Unified foundation model

We train a unified foundation model on nine canonical benchmark cases and evaluate it on 27 diverse test cases. The unified foundation model demonstrates strong generalization across all test categories and consistently reduces the misfit relative to the baseline k–ω model in Fig. S23.

We introduce the normalized misfit, denoted $\hat{e}$, to quantify the improvement of the unified foundation model relative to the single-case trained model and to the baseline k–ω model. Let e_{unified}, e_{single}, and e_{base} denote the misfits of the unified foundation model, the single-case trained model, and the baseline model, respectively, and define

$$\hat{e} = \frac{e_{\text{unified}} - e_{\text{single}}}{e_{\text{base}} - e_{\text{single}}}. \tag{S6}$$

By construction, $\hat{e} = 0$ corresponds to performance equal to the single-case trained model and $\hat{e} = 1$ corresponds to the baseline; values $\hat{e} < 1$ indicate improvement relative to the baseline while values $\hat{e} > 1$ indicate degraded performance. For the rectangular duct case shown in Fig. S22, the unified foundation model attains $\hat{e}$ well below unity, indicating better performance relative to the baseline model; the baseline model has $\hat{e} = 1$ and the single-case trained model has $\hat{e} = 0$ by definition.

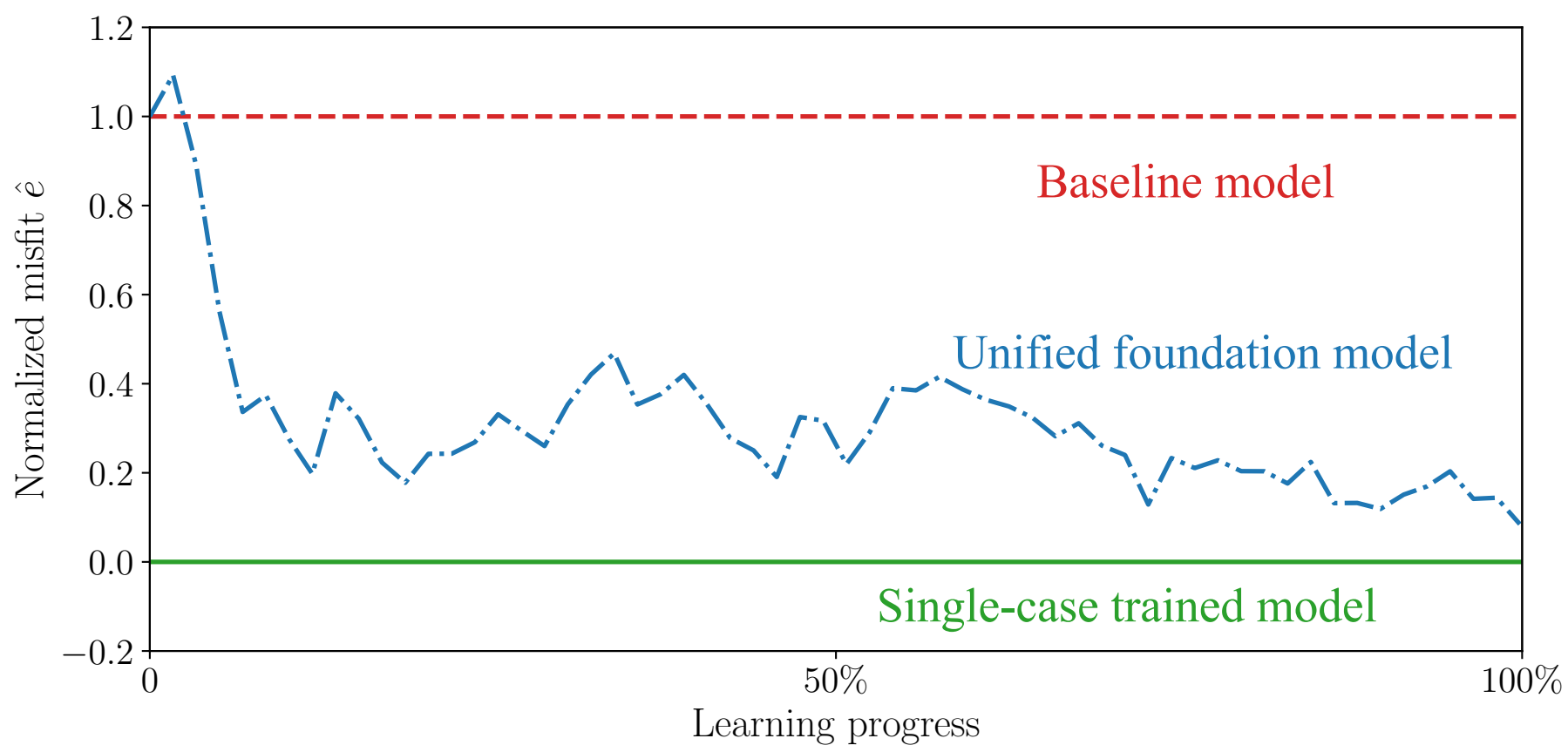

Figure S22: Illustration of the normalized misfit metric $\hat{e}$ used to compare turbulence model performance. The single-case trained model, the unified foundation model, and the baseline k–ω model are shown for reference. The vertical axis shows the normalized misfit $\hat{e}$, defined such that the single-case trained model is fixed at $\hat{e} = 0$ and the baseline model is fixed at $\hat{e} = 1$, and the horizontal axis indicates learning progress.

The normalized misfit $\hat{e}$ across all nine training cases and 27 test cases summarizes the good generalization behavior of the unified foundation model across four flow categories, as shown in Fig. S23. For attached boundary layers, the unified foundation model attains performance comparable to the baseline on non-trained cases, reflecting the effect of the imposed physical consistency constraints. For free-shear and secondary flows, the unified foundation model yields consistently lower misfits than the baseline k–ω model, indicated by the blue polygon lying inside the baseline ring. For separated flows, the unified foundation model substantially reduces misfit relative to the baseline model in most cases, while achieving only comparable performance for the most challenging configurations, including the bump with the greatest height (42 mm) and the periodic hill with the steepest slope ($\alpha_\mathrm{p} = 0.8$). Overall, the normalized misfit distribution demonstrates broad generalization across all four flow categories and simultaneously highlights specific cases of separated flows where targeted refinement is most needed.

Attached boundary layers The unified foundation model matches the baseline k–ω model in eight cases of attached boundary layers, demonstrating good accuracy and generalization across Reynolds numbers and geometries. We evaluate the model on channel flows at $\mathrm{Re} = 3300$, 7890, and 8×10^7, on a flat plate flow, with results shown in Fig. S24. We also test the model on two S809 airfoil cases with low angles of attack (AOA $= 1°$ and $5°$) and two NACA 0012 airfoil cases with low angles of attack (AOA $= 10°$ and $15°$), with results shown in Fig. S28 and Table S8. For the four airfoil cases at low angles of attack, where the flow remains fully attached, all models perform similarly.

The unified foundation model accurately reproduces near-wall mean velocity profiles across canonical wall-bounded flows and Reynolds numbers. Profiles of u^+ versus y^+ (log scale) for DNS data, the baseline k–ω model, and the unified foundation model are shown in Fig. S24. Because the u^+–y^+ relation reflects momentum diffusion from the wall, it provides a sensitive benchmark for turbulence models. At $\mathrm{Re} = 3300$, it follows DNS in the viscous sublayer ($y^+ \lesssim 5$) and buffer layer ($5 \lesssim y^+ \lesssim 30$), and it maintains agreement in the logarithmic region ($30 \lesssim y^+ \lesssim 300$), as shown in Fig. S24a. At $\mathrm{Re} = 7890$, the predicted profile remains consistent with DNS and exhibits behavior similar to the baseline model over the entire wall-normal range, as shown in Fig. S24b. For the plane channel flow at $\mathrm{Re} = 8 \times 10^7$, the velocity profile lies predominantly in the logarithmic region beyond the immediate near-wall layer. In this regime, the unified foundation model preserves the correct logarithmic slope but slightly overpredicts the mean velocity relative to DNS over most of the wall-normal range, as shown in Fig. S24c. In the zero pressure gradient flat plate flow, the unified foundation model captures the expected boundary-layer development and produces velocity profiles

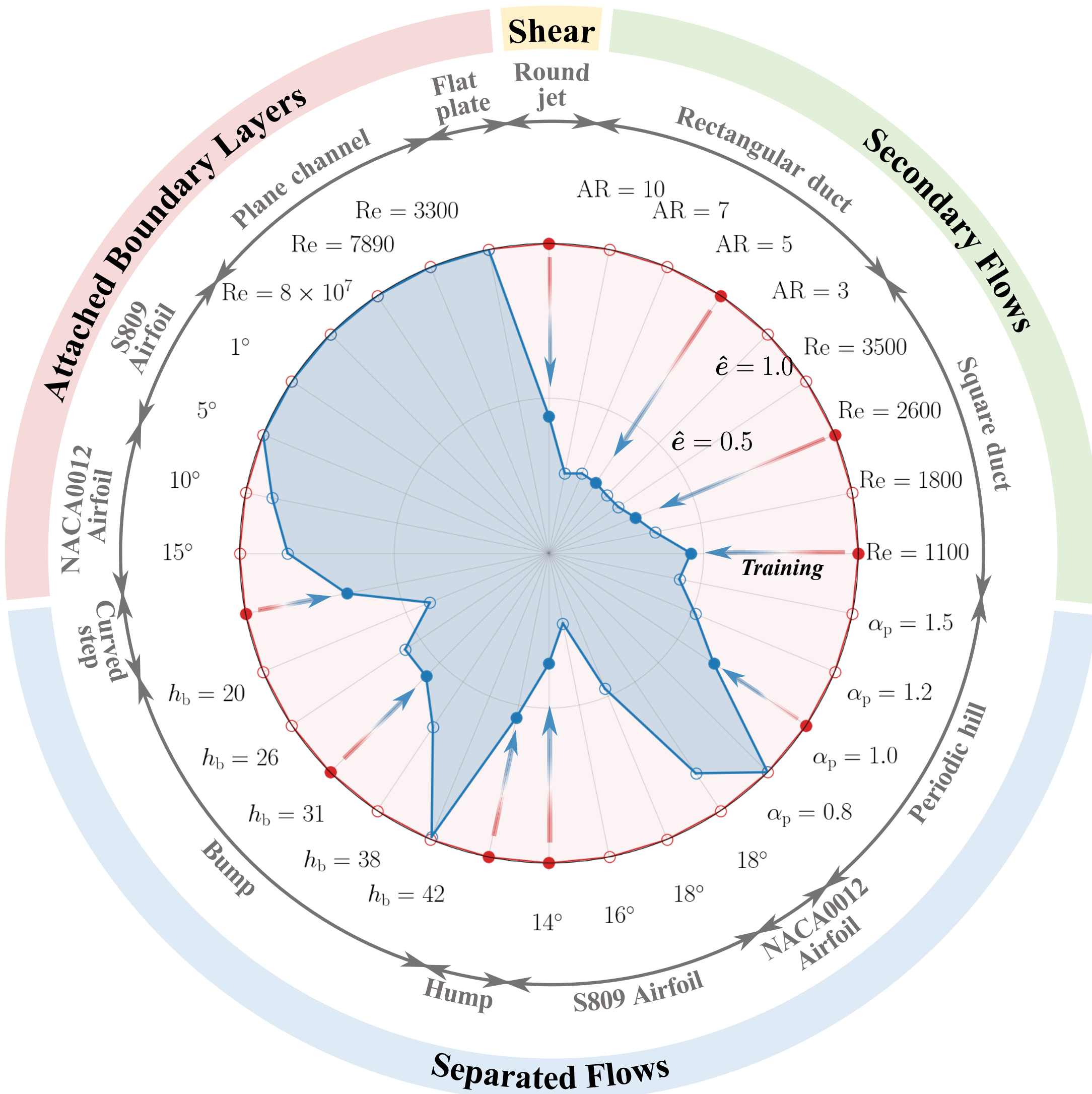

Figure S23: Performance evaluation of the **unified foundation model** across **four benchmark flow categories**, shown as a radar chart with comparison to the baseline k–ω model. The plot includes attached boundary layers, secondary flows, separated flows, and free-shear flows and spans multiple configurations. The normalized misfit $\hat{e}$ is shown as the radial coordinate. The baseline model corresponds to $\hat{e} = 1$ on all axes and is shown as a red curve with round markers. The unified foundation model is shown as a blue filled polygon with markers. Individual test cases are shown along the radial axes and are annotated by parameters such as Reynolds number, aspect ratio, and angle of attack. Across most test cases, the blue filled polygon lies inside the red ring.

comparable to those of the baseline k–ω model, indicating robustness beyond the channel-flow configuration, as shown in Fig. S24d.

These results highlight the unified foundation model's strong generalizability. The model is not trained on any of these cases, yet it captures geometry-agnostic and physically grounded features of near-wall turbulence. This behavior arises from enforcing physical consistency within the turbulence model rather than case-specific training, enabling transfer across attached boundary layer flows.

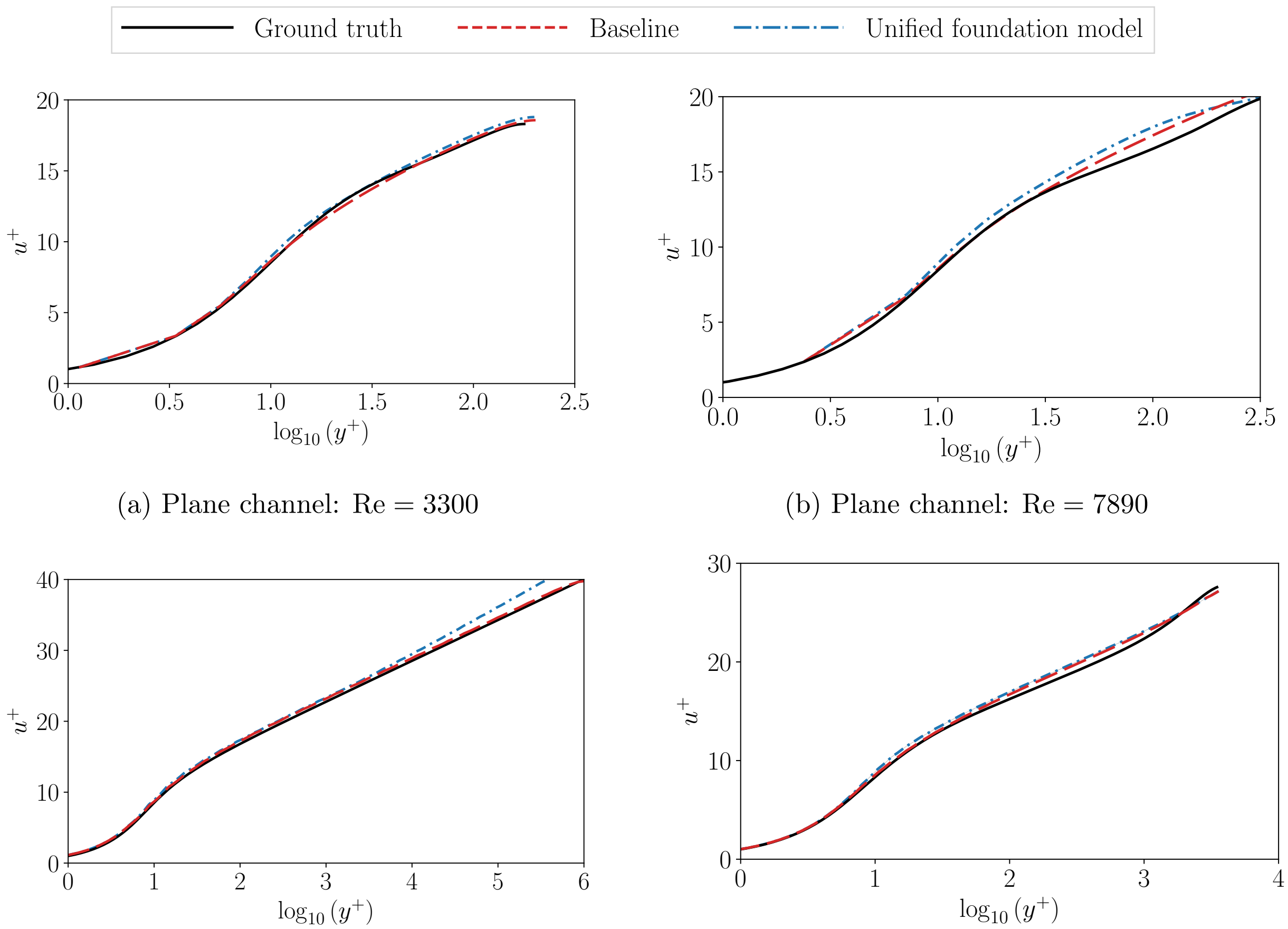

(a) Plane channel: Re = 3300

(b) Plane channel: Re = 7890

(c) Plane channel: Re = 8×10^7

(d) Zero pressure gradient flat plate

Figure S24: Performance evaluation of the **unified foundation model** in **attached boundary layers** against the ground truth and the baseline k–ω model. Mean velocity profiles u^+ are plotted for plane channel flows at (a) Re = 3300, (b) Re = 7890, (c) Re = 8×10^7, and (d) zero pressure gradient flat plate flow; the x-axis denotes the wall-normal coordinate $\log_{10}(y^+)$.

Free-shear flow The unified foundation model reproduces centerline velocity decay in a round jet with higher fidelity than the baseline k–ω model in Fig. S25f. We evaluate the model on the same jet flow case used for training. Compared to high-fidelity DNS data, which show a gradual velocity decay downstream, the unified foundation model closely follows the DNS curve across the entire range. It captures jet entrainment and turbulent mixing more accurately than the baseline model, particularly in the far-field region where the baseline model typically underpredicts the decay. The velocity profiles along the wall-normal direction, which are not directly observed during training, are also predicted with improved accuracy (Fig. S25a–e). This indicates that the unified foundation model generalizes well beyond the observed quantities and provides a more consistent representation of the underlying flow field.

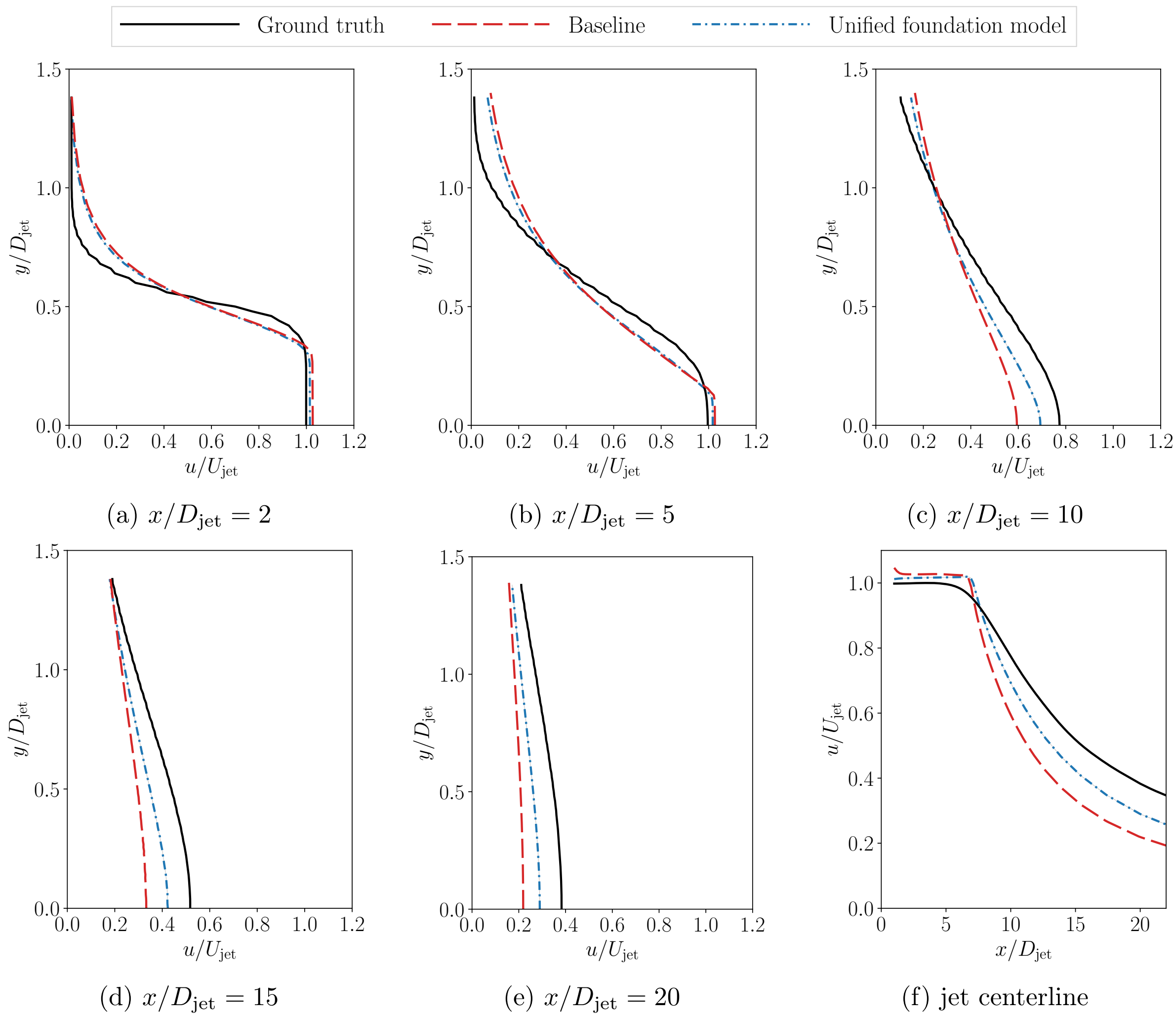

Figure S25: Performance evaluation of the **unified foundation model** in a **round jet** against the ground truth and the baseline k–ω model. Velocity profiles U/U_{jet} are shown at multiple downstream locations, plotted against the normalized wall-normal coordinate y/D_{jet} for subfigures (a)–(e), while the normalized centerline velocity is presented as a function of the downstream distance $x_{\mathrm{center}}/D_{\mathrm{jet}}$ in (f).

Secondary flows The unified foundation model accurately predicts secondary flows across a wide range of duct configurations, generalizing beyond its training conditions. We evaluate the model on eight cases as shown in Fig. S26 and S27: four square ducts at decreasing Reynolds numbers (Re = 3500, 2600, 1800, 1100) and four rectangular ducts at increasing aspect ratios (AR = 3, 5, 7, 10). Among these, the square duct at Re = 1100, 2600 and the rectangular duct at AR = 5 appear in the training set; all other cases serve as generalization tests. For the square ducts, lower Reynolds numbers increase the sensitivity of secondary flow structures to turbulence model accuracy due to stronger viscous effects. Across all Reynolds numbers, the unified foundation model outperforms the baseline k–ω model, maintaining closer agreement with DNS results in Fig. S26. For the rectangular ducts, increasing the aspect ratio amplifies and distorts secondary flows, leading to anisotropic patterns that the baseline k–ω model fails to capture. The unified foundation model captures both the amplification and spatial shifting of secondary vortices with aspect ratio, preserving their qualitative structure and quantitative magnitude in Fig. S27. These results confirm that the model generalizes effectively to both Reynolds number and geometric extrapolation, demonstrating robustness for complex secondary flows beyond the training set.

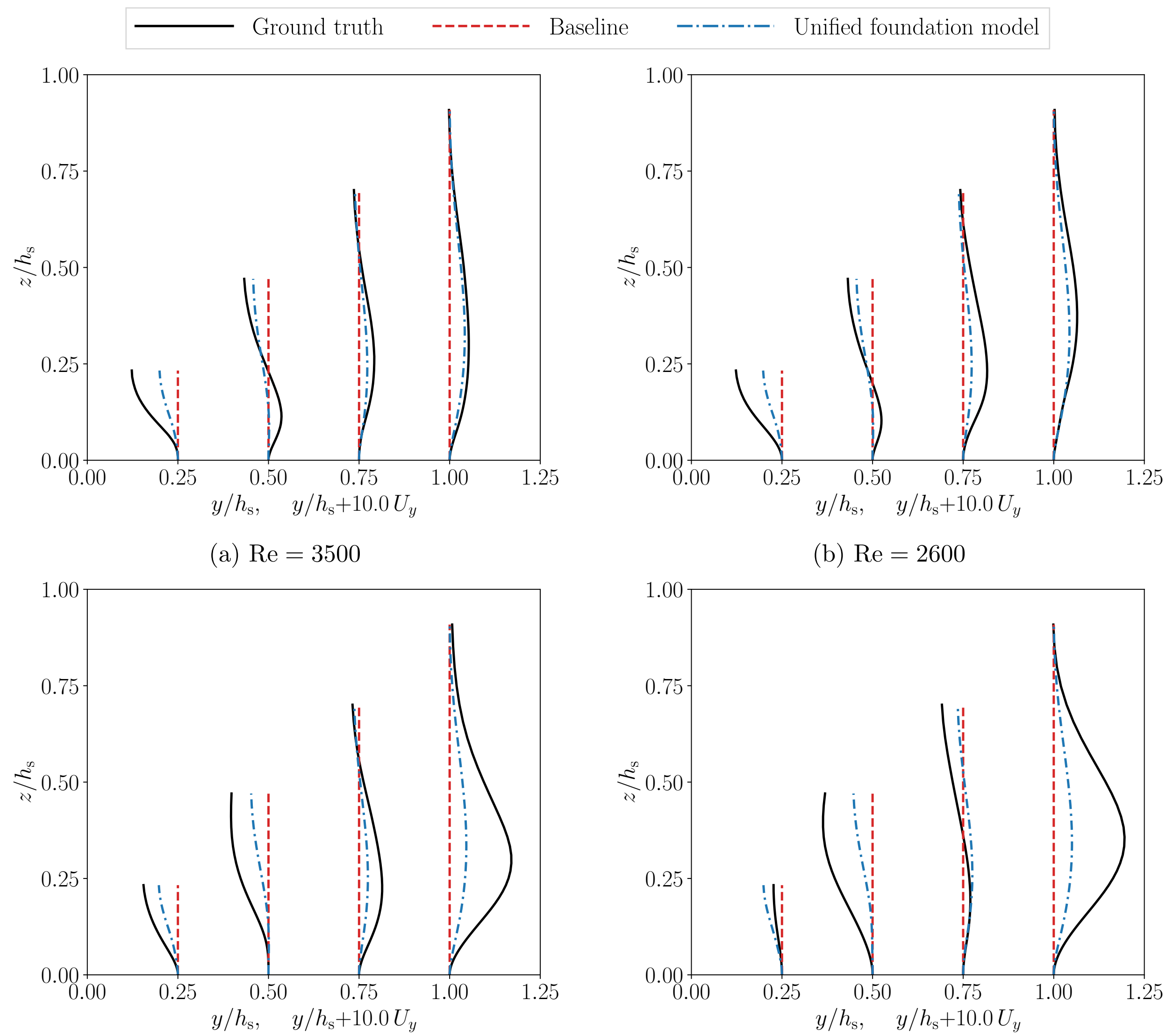

(a) Re = 3500

(b) Re = 2600

(c) Re = 1800

(d) Re = 1100

Figure S26: Performance evaluation of the **unified foundation model** for secondary flow in **square duct configurations** against the ground truth and the baseline k–ω model. Cross-sectional secondary velocity profiles U_y are shown at four Reynolds numbers: (a) Re $= 3500$, (b) Re $= 2600$, (c) Re $= 1800$ and (d) Re $= 1100$. Each panel plots U_y at multiple wall-normal locations z/h_s, where h_s denotes half the duct height; horizontal offsets of $+10.0\,U_y$ are applied for visualization.

Separated flows The unified foundation model improves prediction accuracy for many separated flows. We evaluate the unified foundation model on seven types of benchmark cases: periodic hill flows with different slope scaling factors $\alpha_p = 0.8, 1.0, 1.2, 1.5$, flow over a bump with different bump heights $h_b = 20\,\text{mm}, 26\,\text{mm}, 31\,\text{mm}, 38\,\text{mm}, 42\,\text{mm}$, flow over a curved step, flow over a hump, S809 airfoil cases at three high angles of attack AOA $= 14°, 16°, 18°$ and NACA 0012 airfoil cases at one high angle of attack (18°). Among these, the periodic hill flow with slope scaling factor $\alpha_p = 1.0$, the S809 airfoil flow at AOA $= 14°$, flow over a curved step, flow over a hump and flow over a bump appear in the training set. All seven types of cases are challenging for linear eddy viscosity models because the local equilibrium assumption and Boussinesq hypothesis fail in separated regions. Test results are shown in Figs. S28–S32 and Table S8.

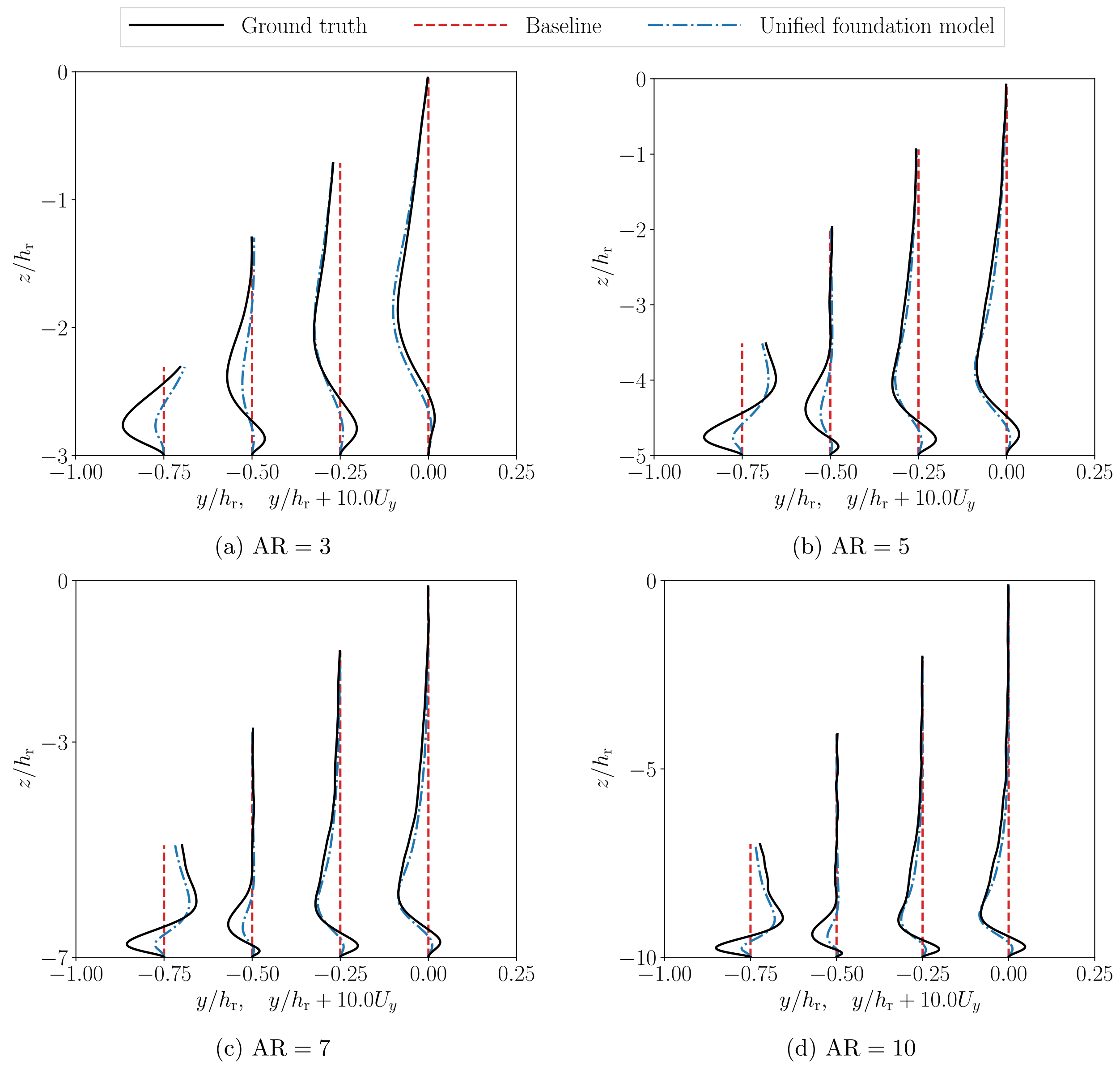

Figure S27: Performance evaluation of the **unified foundation model** for secondary flow in **rectangular duct configurations** against the ground truth and the baseline k–ω model. Cross-sectional secondary velocity profiles U_y are shown for four different aspect ratios (a) AR = 3, (b) AR = 5, (c) AR = 7 and (d) AR = 10. Each panel plots U_y at multiple wall-normal locations z/h_r, where h_r denotes half the height of the rectangular duct; horizontal offsets of $+10.0\,U_y$ are applied for visualization.

The unified foundation model improves predictions over the baseline k–ω model for strongly separated flows, particularly in airfoil and periodic hill configurations. For the S809 airfoil, the unified foundation model generalizes accurately across a wide range of angles of attack, closely following experimental trends in both lift and drag (Fig. S28). In the regime with large flow separation, the baseline model substantially overpredicts lift and underpredicts drag, failing to capture the nonlinear separation-driven transitions, especially at AOA = 18°. In contrast, the unified foundation model reproduces the sharp rise in c_d and the drop in c_l with higher fidelity. Similar improvements are observed for periodic hill flows with varying slope steepness (Fig. S29),

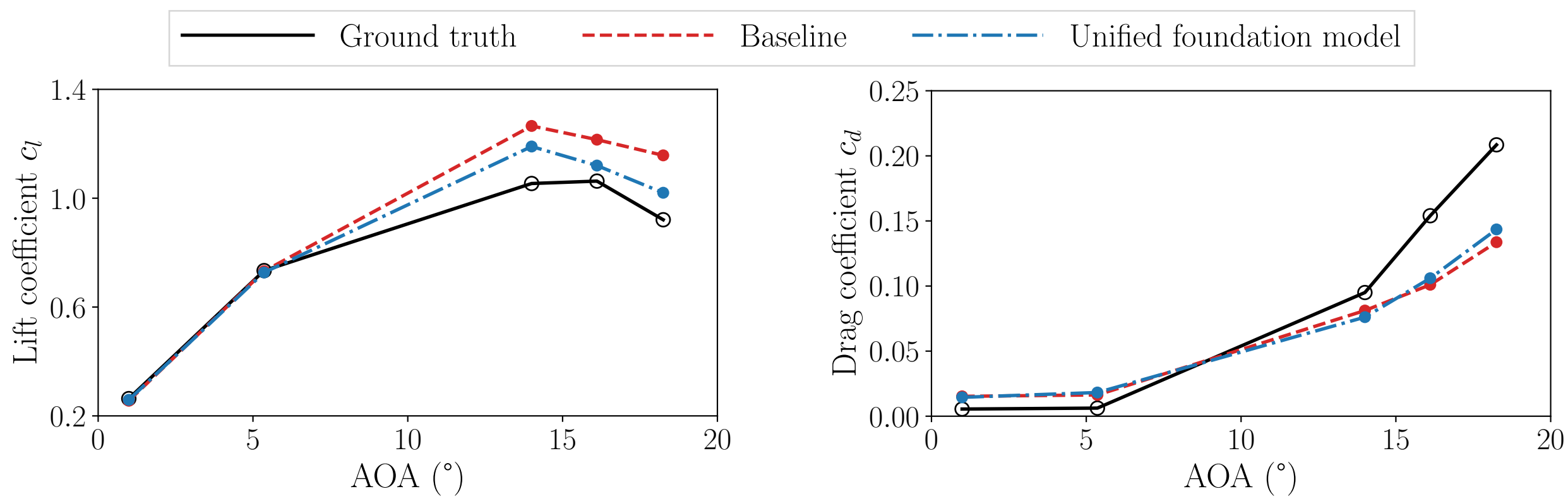

(a) Lift coefficient c_l (b) Drag coefficient c_d

Figure S28: Performance evaluation of the **unified foundation model** for **S809 airfoil flow** across angles of attack against the ground truth and the baseline k–ω model. Lift coefficient c_l (left) and drag coefficient c_d (right) are shown as functions of the angle of attack (AOA).

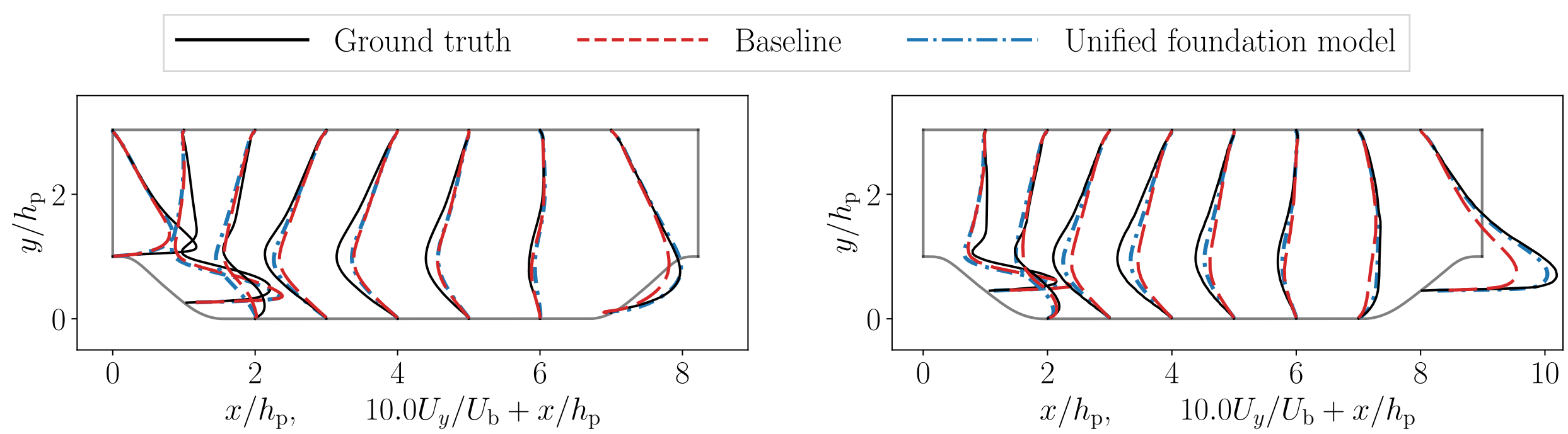

(a) Periodic hill, $\alpha = 0.8$ (b) Periodic hill, $\alpha = 1.0$

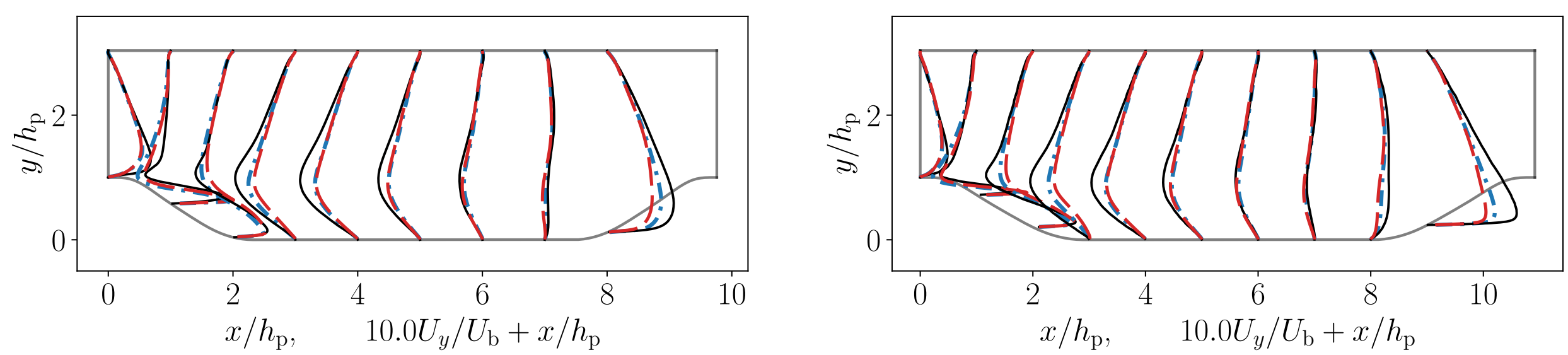

(c) Periodic hill, $\alpha = 1.2$ (d) Periodic hill, $\alpha = 1.5$

Figure S29: Performance evaluation of the **unified foundation model** for **periodic hill flows**, in comparison with the ground truth and the baseline k–ω model. Vertical velocity profiles U_y, normalized by the bulk velocity U_b, are shown for periodic hill configurations with slope scaling factors (a) $\alpha_p = 0.8$, (b) $\alpha_p = 1.0$, (c) $\alpha_p = 1.2$, and (d) $\alpha_p = 1.5$. The profiles are plotted as $10\,U_y/U_b + x/h_p$ versus the normalized wall-normal coordinate y/h_p at multiple streamwise locations x/h_p.

where steeper slopes intensify separation and delay reattachment. Across all slopes, the unified foundation model reproduces the asymmetric development of the separated shear layer and its downstream recovery more faithfully than the baseline predictions.

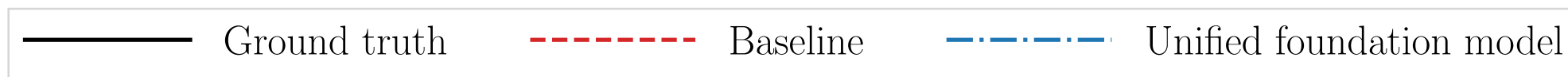

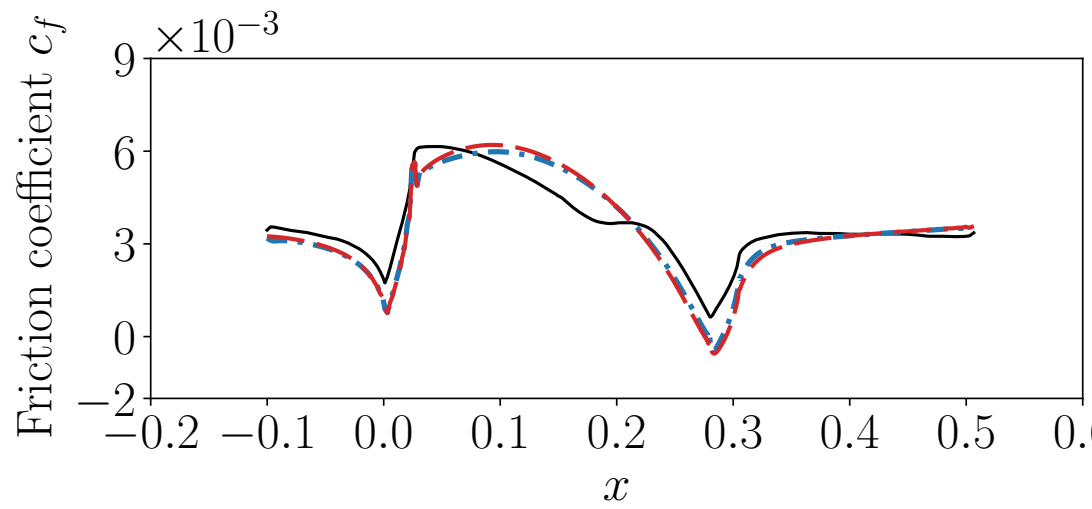

(a) Bump, height = 20mm

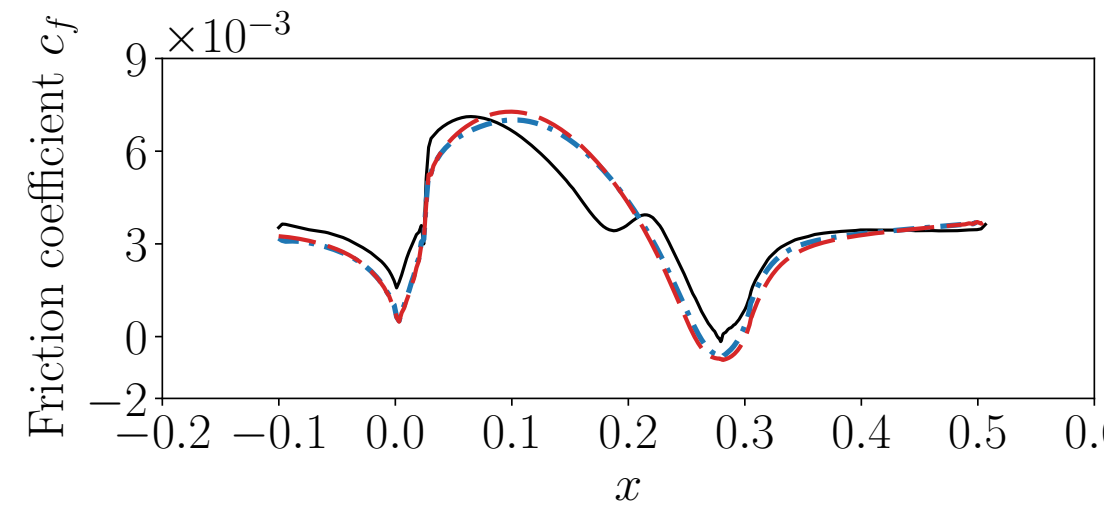

(b) Bump, height = 26mm

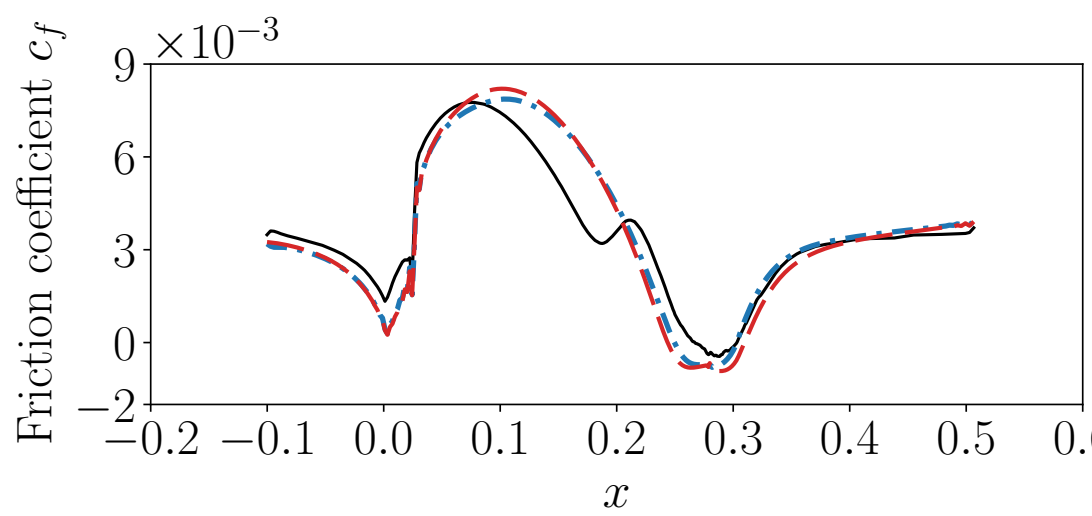

(c) Bump, height = 31mm

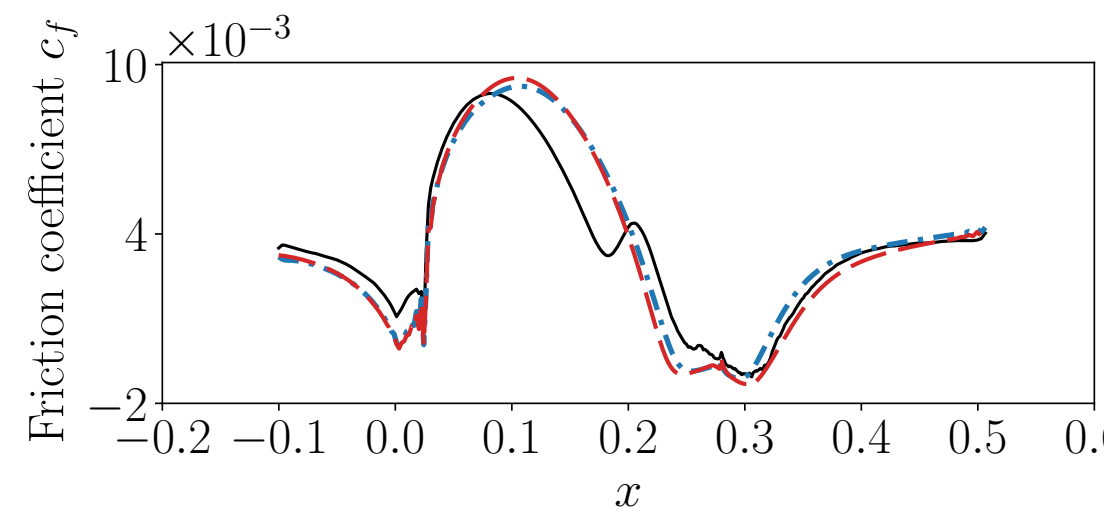

(d) Bump, height = 38mm

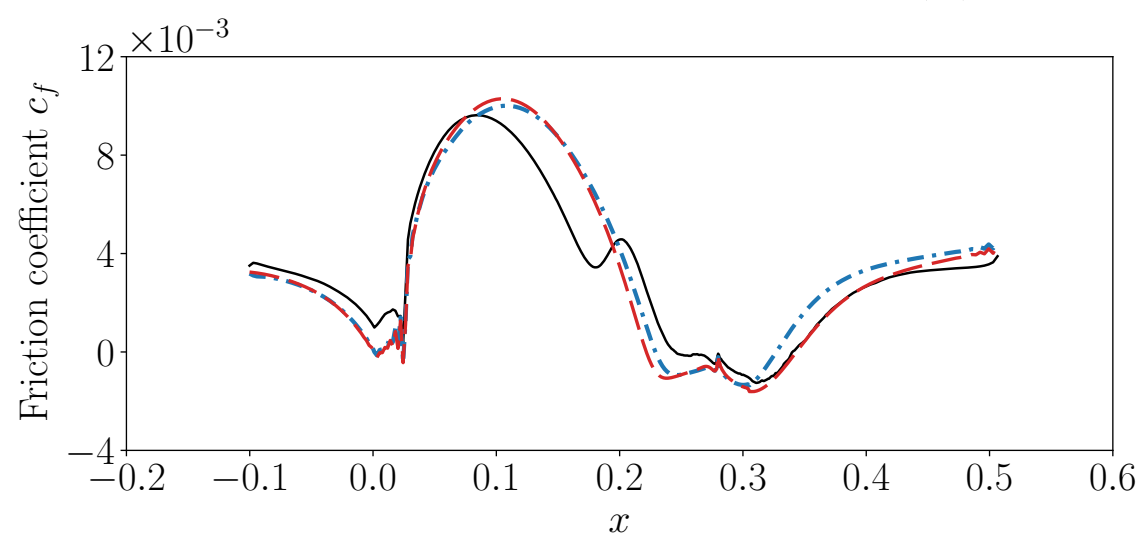

(e) Bump, height = 42mm

Figure S30: Performance evaluation of the **unified foundation model** for **bump flows** against the ground truth and the baseline k–ω model. Streamwise distributions of the wall skin-friction coefficient c_f are shown along the lower surface for bump heights of (a) 20 mm, (b) 26 mm, (c) 31 mm, (d) 38 mm, and (e) 42 mm.

For cases with incipient or moderate separation, the unified foundation model exhibits performance that is comparable to, and occasionally slightly better than, the baseline k–ω model. In the bump flows (Fig. S30), the predicted skin-friction coefficient distributions closely follow the baseline across all bump heights, with only minor deviations near separation and reattachment and nearly identical results for the largest bump height (Fig. S30e). In the hump case, the unified foundation model achieves higher overall accuracy than the baseline model, especially producing fuller near-wall velocity profiles and improved gradients near separation and reattachment, particularly at $x/c_{\text{hump}} = 0.80$ (Fig. S31). For the curved step case, the unified model yields slightly more accurate normalized velocity profiles downstream of separation, indicating improved representation of near-wall behavior (Fig. S32a). The skin-friction coefficient distribution, though not observed during training, shows more accurate predictions of separation and reattachment locations (Fig. S32b), indicating improved generalization and a more consistent representation of the flow field. For the NACA 0012 airfoil, the unified foundation model achieves slightly better performance than the baseline model under attached-flow conditions (AOA $= 10°, 15°$) as shown in Table S8, while at AOA $= 18°$ the unified foundation model modestly reduces lift overprediction relative to the baseline, suggesting limited but consistent gains in post-separation regimes.

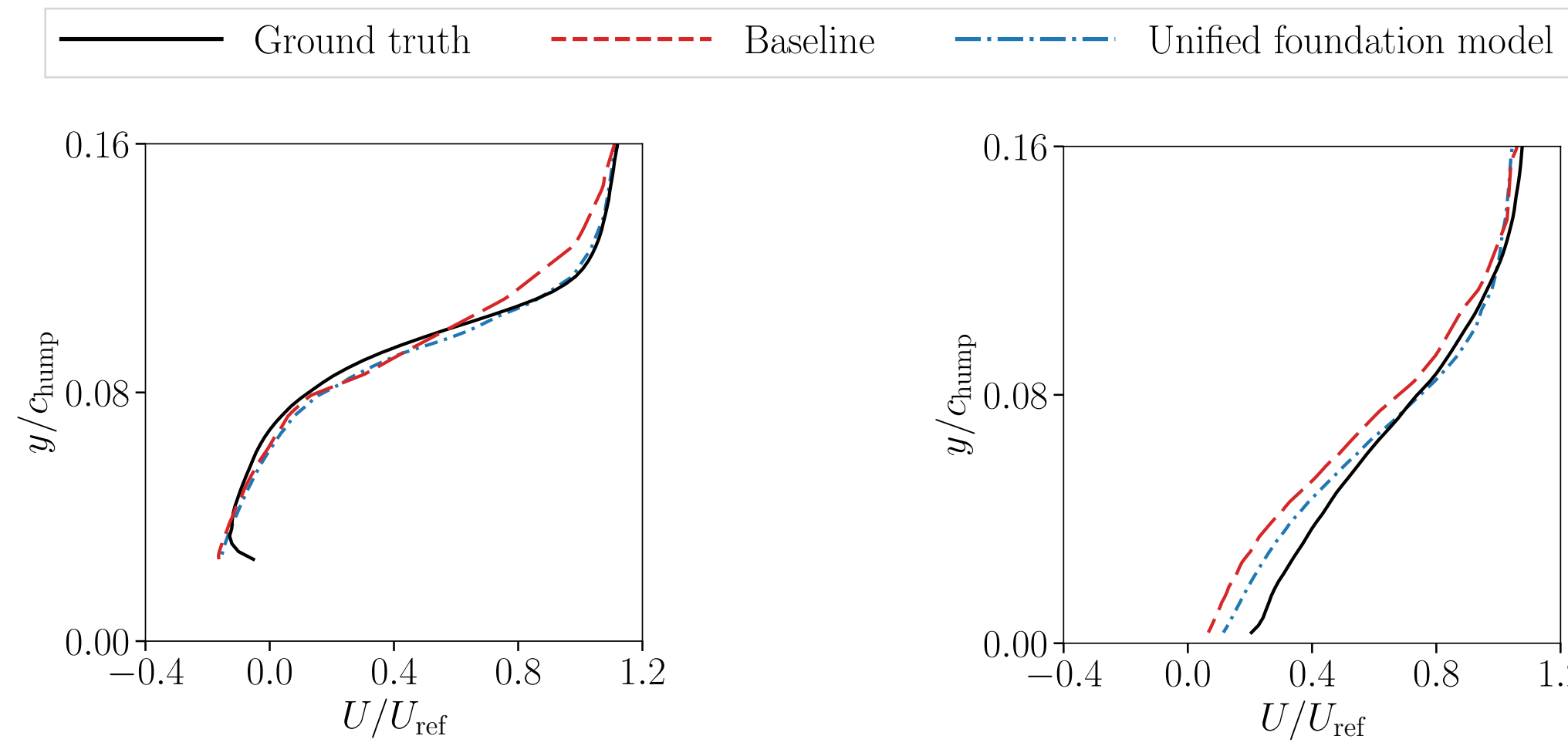

(a) $x/c_{\text{hump}} = 0.8$

(b) $x/c_{\text{hump}} = 1.2$

Figure S31: Performance evaluation of the **unified foundation model** in **hump flow** against the ground truth and the baseline k–ω model. Mean streamwise velocity profiles U/U_{ref} are shown at two streamwise locations, (a) $x/c_{\text{hump}} = 0.80$ and (b) $x/c_{\text{hump}} = 1.20$, representing regions upstream and downstream of separation, respectively. The y-axis represents the wall-normal coordinate, normalized by c_{hump}.

NACA 0012 cases	Ground truth	Baseline k–ω model	**Unified foundation model**
AOA $= 10°$	1.08	1.12	**1.11**
AOA $= 15°$	1.49	1.65	**1.58**
AOA $= 18°$	1.01	1.75	**1.69**

Table S8: Performance evaluation of the **unified foundation model** for **NACA 0012 airfoil flow** against the ground truth and the baseline k–ω model. Lift coefficient predictions are listed for the NACA 0012 airfoil at three angles of attack (AOA $= 10°,\ 15°,\ 18°$).

Complex 3D flows The unified foundation model yields predictions that are either improved or comparable to the baseline k–ω model across the complex three-dimensional cases, without degrading performance (Fig. S33).

In the generic car case, the unified foundation model and the baseline model predict a similar separated wake, with nearly identical wake extent (see Fig. S33a). Consistent with this observation, the predicted drag coefficients are also very similar for the two models (Table S11). In this configuration, the unified foundation model performs on par with the baseline, without noticeable degradation. The quadratic term $g^{(2)}$ is manually deactivated due to its sensitivity, without affecting the prediction. In the asymmetric 3D diffuser, the unified foundation model closely follows the gradual downstream decay of the wall skin friction c_f observed in the ground truth, whereas the baseline model markedly overpredicts c_f and maintains elevated values far downstream (see Fig. S33b). The generic aircraft serves as a benchmark configuration in the aerospace community and provides a stringent test across flow regimes. At a low angle of attack (pre-stall, AOA $= 7.05°$), the baseline model performs well, and the unified foundation model recovers this behavior while slightly improving the predictions of lift and drag. At a high angle of attack (post-stall, AOA $= 21.47°$), where strong separation occurs, the baseline model shows significant deficiencies, whereas the unified model improves the predictions, particularly for drag (see Table S9). The flow over the wing is also better captured, as indicated by improved skin-friction distributions (see Fig. S33c) and pressure coefficients at multiple spanwise sections (see Fig. S34). The unified model reduces the excessive spanwise flow predicted by the baseline model and

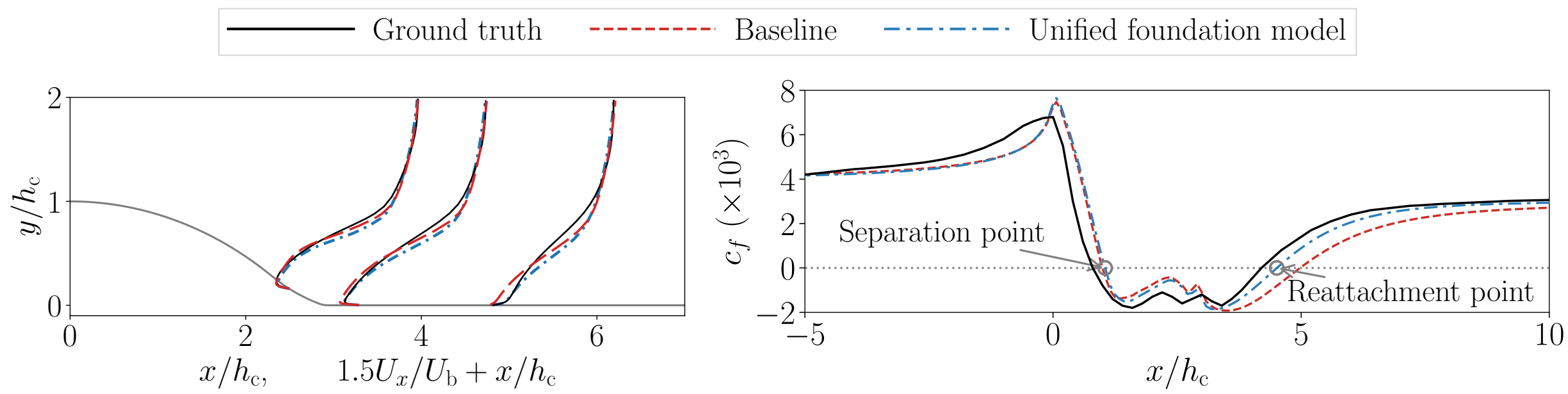

(a) Streamwise velocity profiles

(b) Friction coefficient

Figure S32: Performance evaluation of the **unified foundation model** in a **curved step flow** against the ground truth and the baseline k–ω model. (a) Streamwise velocity profiles U_x, normalized by the bulk velocity U_b, are shown at multiple streamwise locations downstream of the curved step. The profiles U_x versus the wall-normal coordinate y normalized by the curved-step height h_c. (b) Skin-friction coefficient distributions c_f, are shown along the streamwise direction x/h_c. Separation and reattachment points are marked using circles.

Angle of attack	7.05°		21.47°	
	c_d	c_l	c_d	c_l
Ground truth	0.16	1.80	0.39	2.39
Baseline k–ω model	0.21	1.77	0.73	2.20
Unified foundation model	**0.20**	**1.81**	**0.47**	**1.75**

Table S9: Drag and lift coefficients (c_d, c_l) for the generic aircraft predicted by the unified foundation model, compared with the ground truth and the baseline k–ω model.

recovers the mid-span flow behavior without large-scale separation, leading to improved pressure predictions at the mid-span and tip sections. In this case, the quadratic term $g^{(2)}$ is also deactivated due to stability issues. Despite these improvements, discrepancies remain at high angle of attack. The lift is underpredicted compared to both the ground truth and the baseline model, and the pressure distributions still deviate from the ground truth, particularly near the root section. These errors indicate that the separation is not fully captured, which limits the accuracy of the integrated force predictions.

Performance on NASA turbulence modeling challenge cases NASA turbulence modeling challenge cases span attached boundary layers, separated flows, and free-shear flows. The attached boundary layer cases include a 2D zero–pressure-gradient flat plate, a 2D fully developed channel flow, and NACA 0012 airfoil with two low angles of attack. The remaining cases comprise an axisymmetric subsonic jet, a 2D wall-mounted hump, and a NACA 0012 airfoil at high angle of attack. The name mappings for all cases are summarized in Table S10. The unified foundation model demonstrates improved overall performance across the NASA turbulence modeling challenge cases compared to the baseline k–ω model shown in Fig. S35, indicating robust generalization within a widely adopted benchmark suite. The attached boundary layer cases (2D zero-pressure-gradient flat plate, 2D fully developed channel flow, and low-angle-of-attack NACA 0012 airfoil) are treated as verification, as baseline models already perform well in these regimes. For these flows, the unified foundation model exhibits performance comparable to the baseline k–ω model, confirming that the proposed training framework preserves established accuracy where the physics are well captured. In contrast, the unified foundation model achieves clear improvements across the remaining three cases (the axisymmetric subsonic jet, the 2D wall-mounted hump and the NACA 0012 at angle of attack of 14°), as reflected by the reduced radial extent of the blue polygon in Fig. S35. For the NACA 0012 airfoil at high angle of attack, i.e., AOA = 18°, the quadratic term $g^{(2)}$ in the constitutive relation is explicitly set to zero to ensure numerical

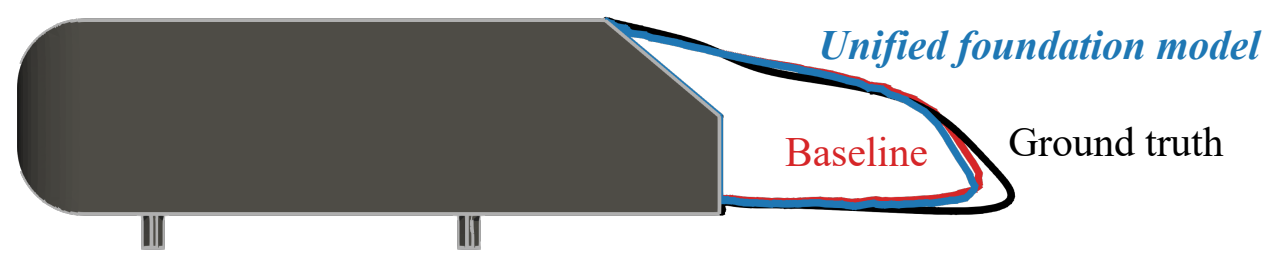

(a) Separation region comparison for generic car

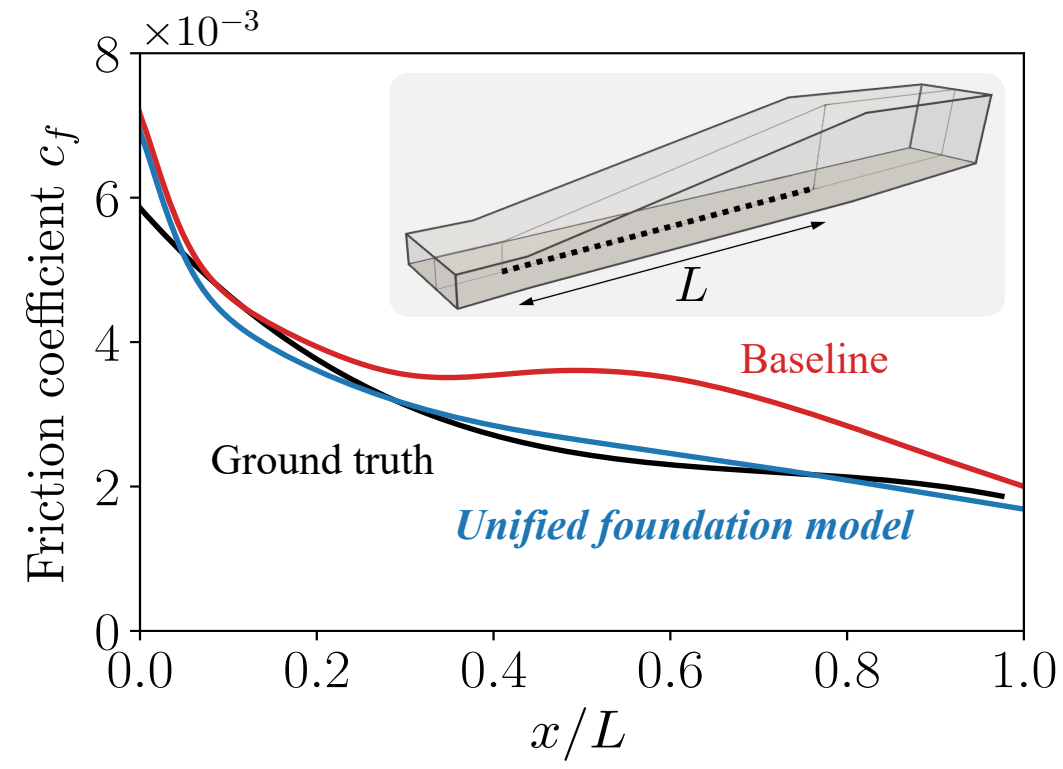

(b) Friction coefficient comparison for asymmetric 3D diffuser

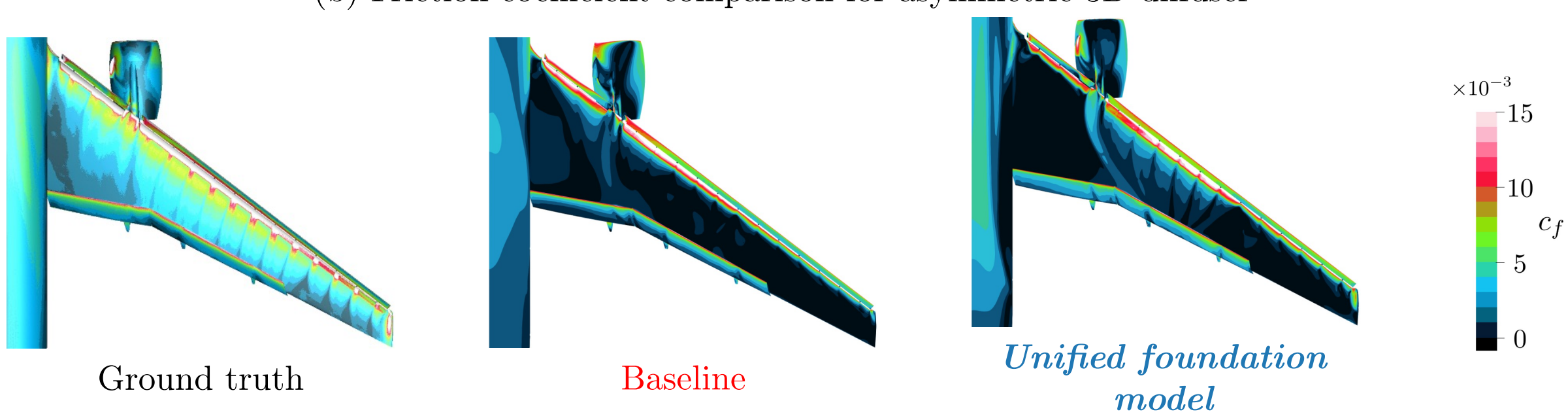

(c) Comparison of skin-friction coefficients on the generic aircraft predicted by different models at AOA = 21.47°

Figure S33: Performance of the **unified foundation model** on **complex three-dimensional configurations**, including a generic car, an asymmetric diffuser, and a generic aircraft, in comparison with the baseline k–ω model and ground truth. Panel (a) shows the predicted separation region for the generic car, where solid contours indicate the wake and the $U_x = 0$ contour delineates flow separation. Panel (b) presents the skin-friction coefficient c_f for the asymmetric diffuser along the bottom-wall midline. Panel (c) shows the surface skin-friction coefficient on the generic aircraft at AOA = 21.47° predicted by the baseline and the unified foundation model, compared with the ground truth [68].

stability. Even under this restriction, the unified foundation model remains competitive and extends predictive coverage relative to the baseline closure in the post-stall regime.

5.2 Specialist turbulence model for separated flows

We develop a specialist turbulence model for separated flows by fine-tuning unified foundation model on benchmark canonical cases that exhibit distinct separation mechanisms. It is then evaluated both within these training categories and on a geometrically more complex three-dimensional configuration.

In this specialist model, we remove the quadratic term $g^{(2)}\mathbf{T}^{(2)}$ and retain only the linear constitutive relation. The coefficient functions $g^{(1)}$ and β are then fine-tuned from the unified foundation model, improving stability while focusing the model capacity on flow separation.

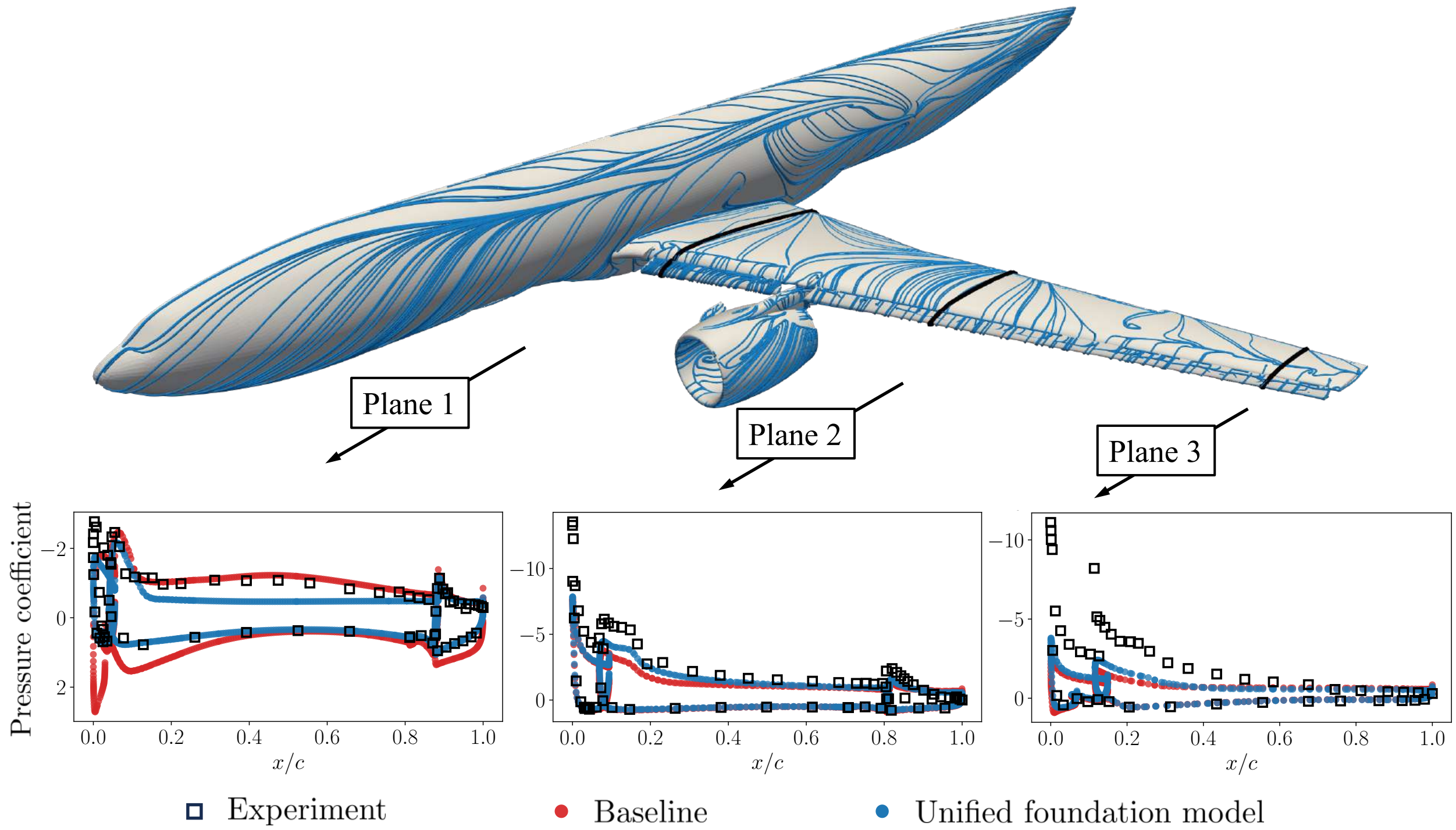

Figure S34: Surface streamlines on the generic aircraft at AOA = 21.47° predicted by the unified foundation model, together with pressure-coefficient distributions at three spanwise sections: Plane 1 (root), Plane 2 (mid-span), and Plane 3 (tip). The baseline model predicts a dominant spanwise flow, whereas the unified model captures the mid-span flow behavior without large-scale separation, consistent with experiments [66]. This improves the pressure prediction at Plane 2 and yields slight improvement at Plane 3. At Plane 1, the unified model improves the pressure distribution on the lower surface, while discrepancies remain on the upper surface; the associated separation behavior contributes to the underprediction of lift.

**Acronym**	**Case description**	**Case library label(s)**
2DZP	2D zero-pressure-gradient flat plate	BL1
2DFDC	2D fully developed channel flow at high Reynolds number	BL2.3
ASJ	Axisymmetric subsonic jet	FS1
2DWMH	2D NASA wall-mounted hump separated-flow	SP5
2DN00	2D NACA 0012 airfoil	BL3.3, BL3.4, SP4.4

Table S10: Summary of cases in the **NASA Turbulence Modeling Challenge** [7], with descriptions and the corresponding labels in our case library (see Table S4).

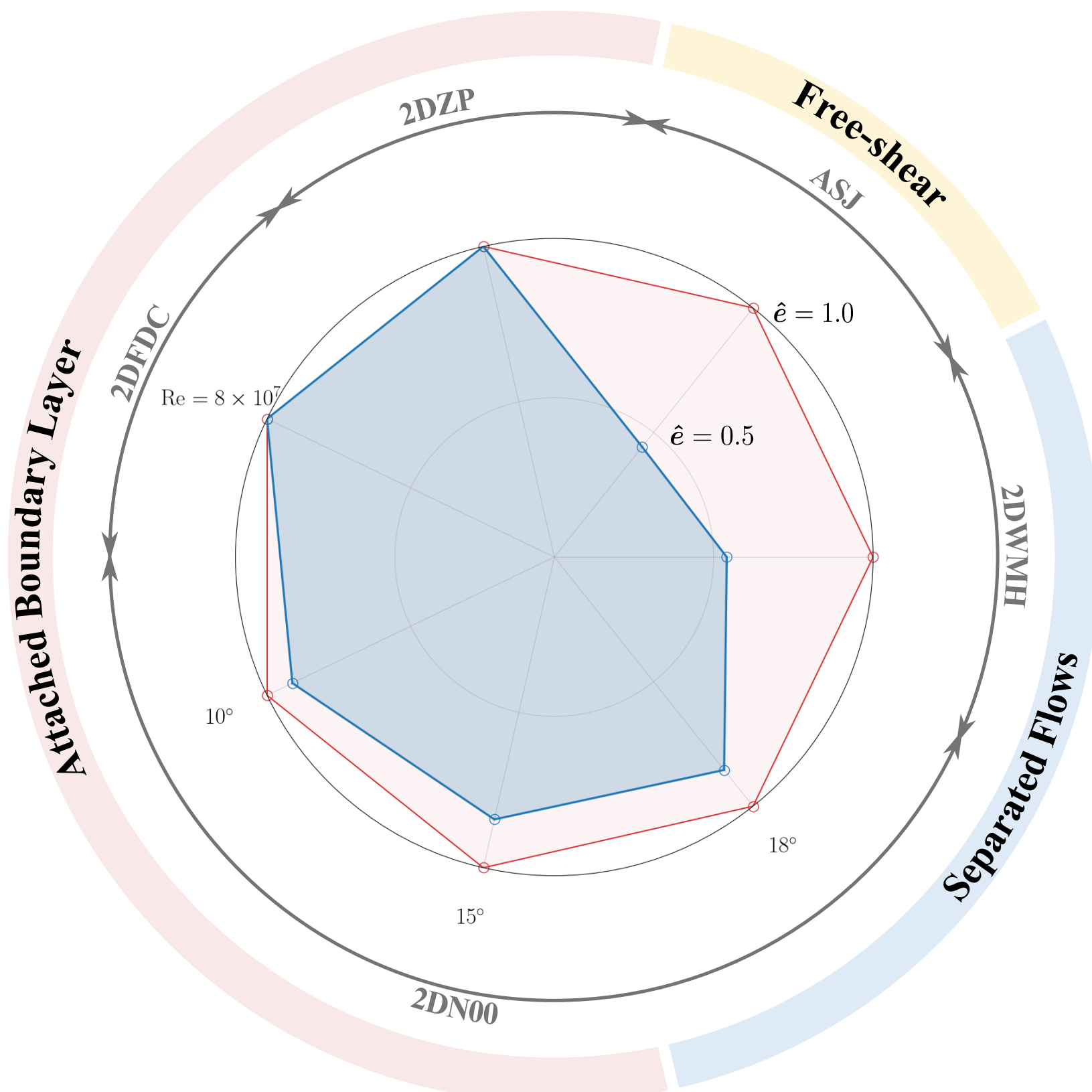

Figure S35: Performance evaluation of the **unified foundation model** on the **NASA challenge cases**. The normalized misfit $\hat{e}$ is shown as the radial coordinate. The radar chart compares $\hat{e}$ across canonical attached boundary layer flows, separated flows, and free-shear flows, with the baseline k–ω model shown for reference, corresponding to the unit red polygon with markers ($\hat{e} = 1$), while the unified foundation model is shown as a blue filled polygon with markers. Across most test cases, the blue filled polygon lies inside the red polygon. The test cases (see Table S10) include 2D zero-pressure-gradient flat plate (2DZP), 2D fully developed channel flow at a high Reynolds number (2DFDC), axisymmetric subsonic jet (ASJ), 2D NASA wall-mounted hump with separated flow (2DWMH), and 2D NACA 0012 airfoils (2DN00).

Fine-tuning results The specialist model improves separation predictions for the two fine-tuning cases (the 42 mm bump and the curved step), as shown by comparisons with the ground truth and the baseline k–ω model in Fig. S36. The specialist model more accurately captures both the onset and reattachment of separation, yielding separation bubbles that closely follow the ground-truth isolines. The improvement is particularly evident for the bump configuration, where the specialist model reproduces the separation extent and recovery with high fidelity. However, the baseline model substantially overpredicts the extent of the separated region, leading to delayed reattachment relative to the reference solution.

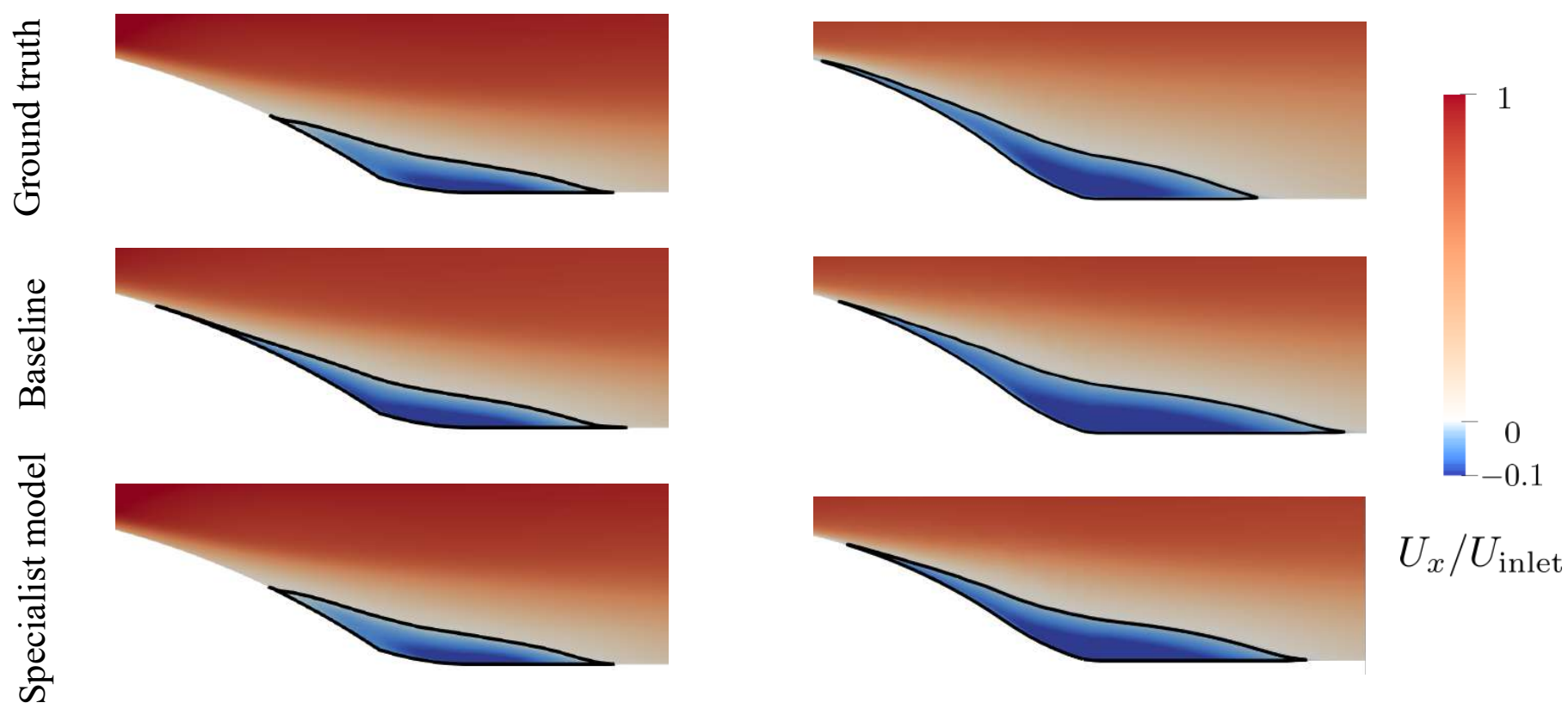

(a) Bump with height 42 mm

(b) Curved step

Figure S36: Fine-tuning performance of the **specialist model for separated flows**. Separation predictions are shown for flows over (a) a **bump** with height 42 mm and (b) a **curved step**, with the ground truth, the baseline k–ω model, and the specialist model arranged from top to bottom. The contours represent the non-dimensional streamwise velocity U_x/U_{inlet}, with the black contour line indicating $U_x = 0$ to delineate the separated recirculation region.

Test results on flows within fine-tuning categories The specialist model generalizes well to flows over bumps, consistently improving separation prediction across a range of geometries. We evaluate this behavior for bump heights of $h_{\text{b}} = 20$, 26, 31, and 38 mm, which fall within the training categories (see Fig. S3), with performance summarized in Fig. S37. Across all bump heights, the specialist model yields smaller misfit values than the baseline model, indicating a consistent reduction in separation-prediction error and that the results approach the single-training reference, which represents the optimized upper bound for these configurations.

Test results on generic car The specialist model for separated flows exhibits improved predictive capability on the three-dimensional generic car (see Fig. S38), providing more accurate drag estimates and wake separation than the baseline and unified foundation models. In this case, the drag coefficient c_d is the key quantity of interest and is summarized in Table S11 for the different models. Relative to the ground truth, the specialist model provides the closest estimate of the drag coefficient.

The specialist model most accurately reproduces the wake structure by appropriately reducing eddy viscosity in the separated region. Wake structures predicted by the different models are compared in Fig. S38a, where the $U_x = 0$ contour delineates the separated region. The specialist model shows the best agreement with the ground truth, accurately reproducing both the size and downstream extent of the separated region. The streamline patterns and the spatial distribution of $-g^{(1)}$ are shown in Fig. S38b to examine the model behavior underlying this improvement. Regions where $-g^{(1)}$ decreases relative to its nominal value of 0.09 are concentrated in the wake, where the eddy viscosity ν_t is locally reduced according to [32]

$$\nu_t = -\frac{g^{(1)} k}{C_\mu \omega}, \tag{S7}$$

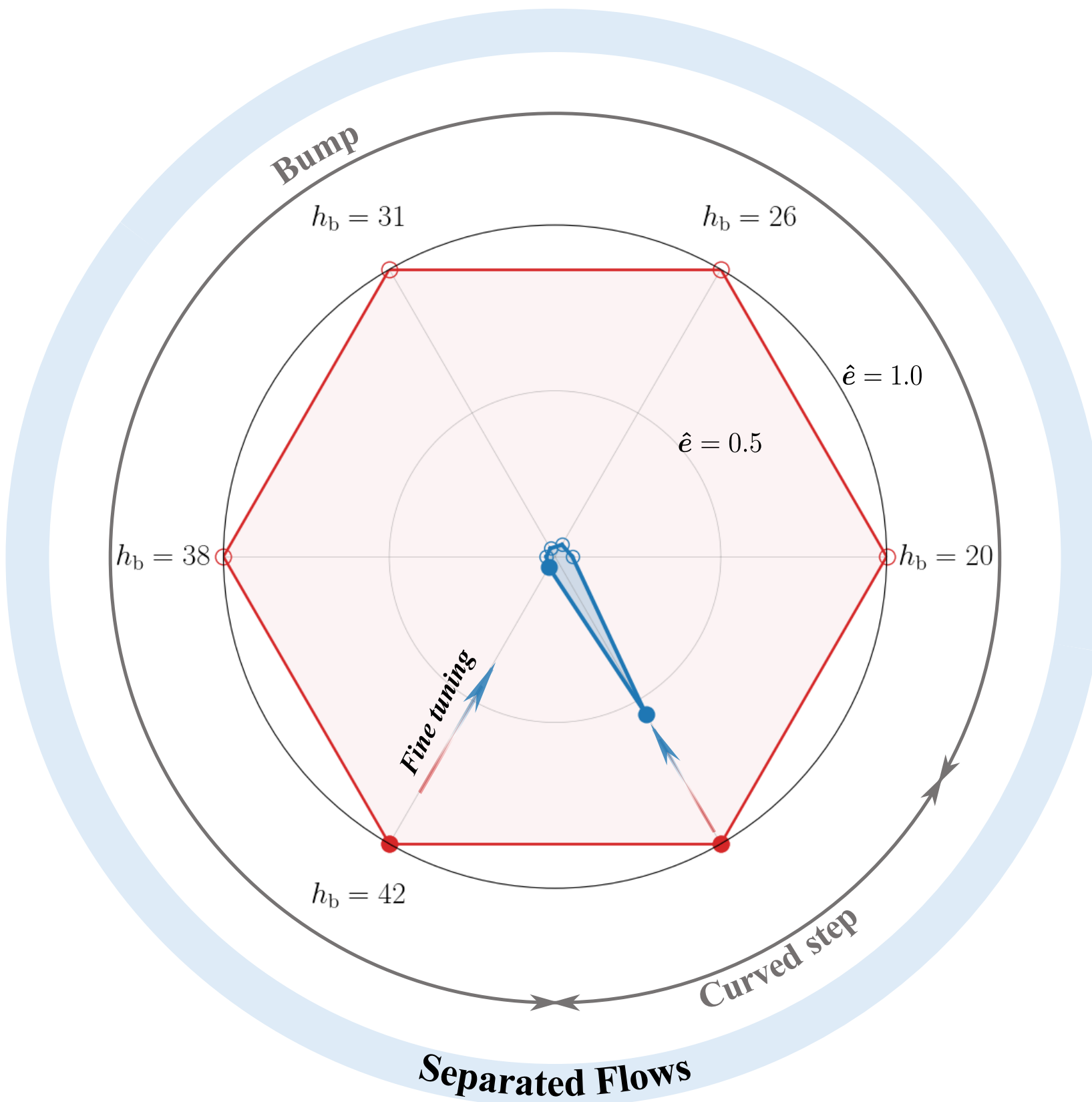

Figure S37: Performance evaluation of the **specialist model for separated flows** within the **training categories**, shown as a radar chart compared with the baseline k–ω model. Bumps with height from 20 to 42 mm ($h_b = 20$–42 mm) and a curved step are included. The radial coordinate represents the normalized misfit $\hat{e}$. The performance of the specialist model is shown as a blue filled polygon, while that of the baseline k–ω model is shown as a red polygon with markers ($\hat{e} = 1$). Arrows indicate the fine-tuning cases, demonstrating additional improvements in the specialist model through further training on specific separated flows.

Model	Drag coefficient
Ground truth	0.248
Baseline k–ω model	0.295
Unified foundation model	0.297
Specialist model	**0.275**

Table S11: Drag coefficient evaluation of the specialist model for separated flows compared to the unified foundation model, the ground truth, and the baseline k–ω model.

with $C_\mu = 0.09$. This localized reduction in ν_t is consistent with enhanced separation and improved agreement with the ground-truth wake structure.

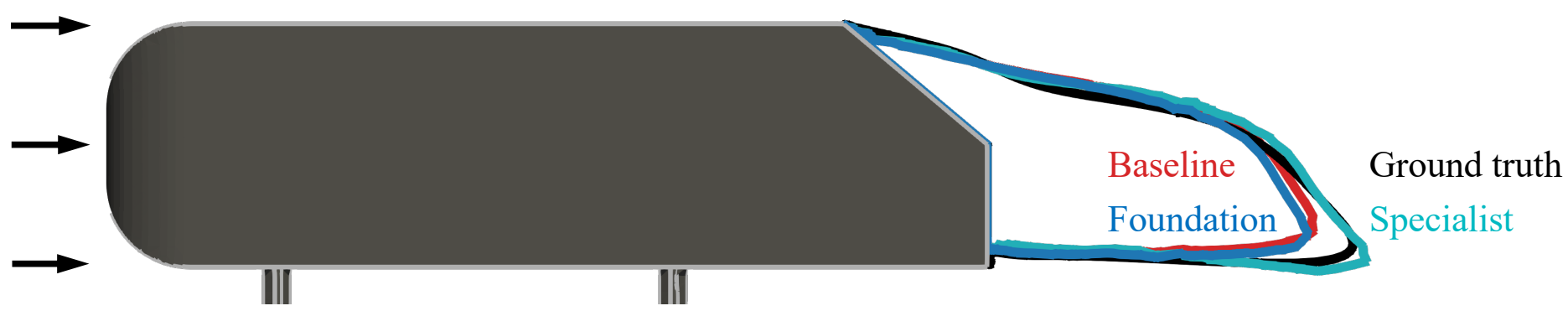

(a) Separated region comparison for generic car

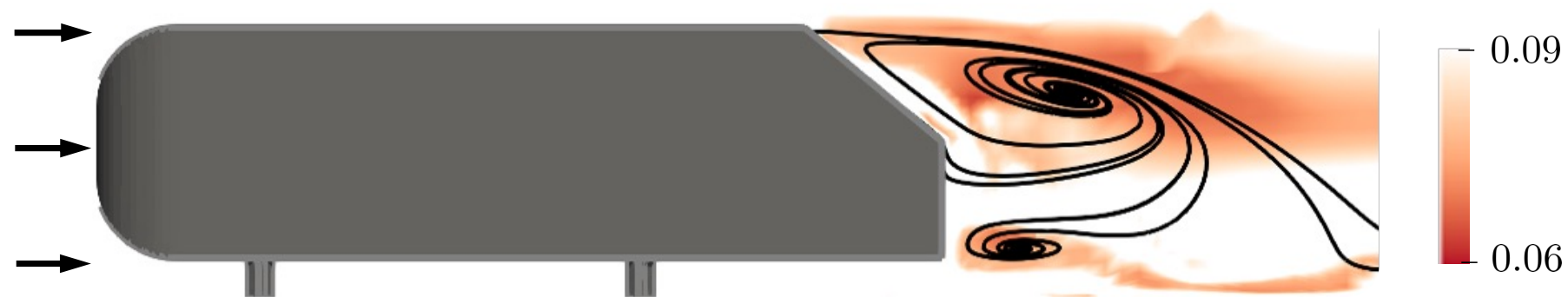

(b) Streamline and $-g^{(1)}$ distribution in the wake region from specialist model

Figure S38: Performance evaluation of the **specialist model for separated flows** on the **generic car** (Ahmed body), showing (a) comparison of the separated wake region, delineated by the $U_x = 0$ contour, for the baseline model, unified foundation model, specialist model and ground truth; (b) streamlines and spatial distribution of the correction coefficient $-g^{(1)}$ predicted by the specialist model. The decreased values of $-g^{(1)}$ indicate a reduction relative to the standard value $C_\mu = 0.09$ used in the baseline k–ω model, within the wake region, as illustrated by the streamlines (solid black lines). The arrows indicate inflow direction.

In summary, the specialist model for separated flows improves prediction of the separation behavior across the training cases, placing onset and reattachment points closer to the ground truth. For unseen flows within the training categories, the model consistently reduces error relative to the baseline, indicating good generalization ability for closely related geometries. When applied to the generic car, the specialist model maintains improved performance, with localized corrections concentrated in the wake region that are consistent with more accurate predictions of the separation extent and associated flow structures.

5.3 Specialist turbulence model for secondary flows with separation

We develop a specialist turbulence model targeting secondary flows with separation and evaluate its performance on three-dimensional configurations. The model reduces the baseline k–ω model's overprediction of separation, more accurately reproduces corner-vortex structures, and successfully captures the interaction between secondary flow and separation in a three-dimensional diffuser.

Fine-tuning results The specialist model improves predictions for both separated and secondary flows. For the curved step, which is governed by flow separation, the specialist model reduces the baseline model's overprediction of the separated region and yields a separation extent closer to the ground truth (Fig. S39a). For the rectangular duct flows, which are characterized by secondary motions, the in-plane velocity fields predicted by the specialist model, the baseline model, and the ground truth are compared in Fig. S39b. For the rectangular duct with aspect ratio AR = 3, the flow exhibits asymmetric corner vortices, with vortices along the horizontal walls extending further in the spanwise direction and weakening those along the vertical walls. The specialist model accurately reproduces this asymmetry and the associated vortex structure, closely matching the ground truth, whereas the baseline model fails to capture any corner vortices.

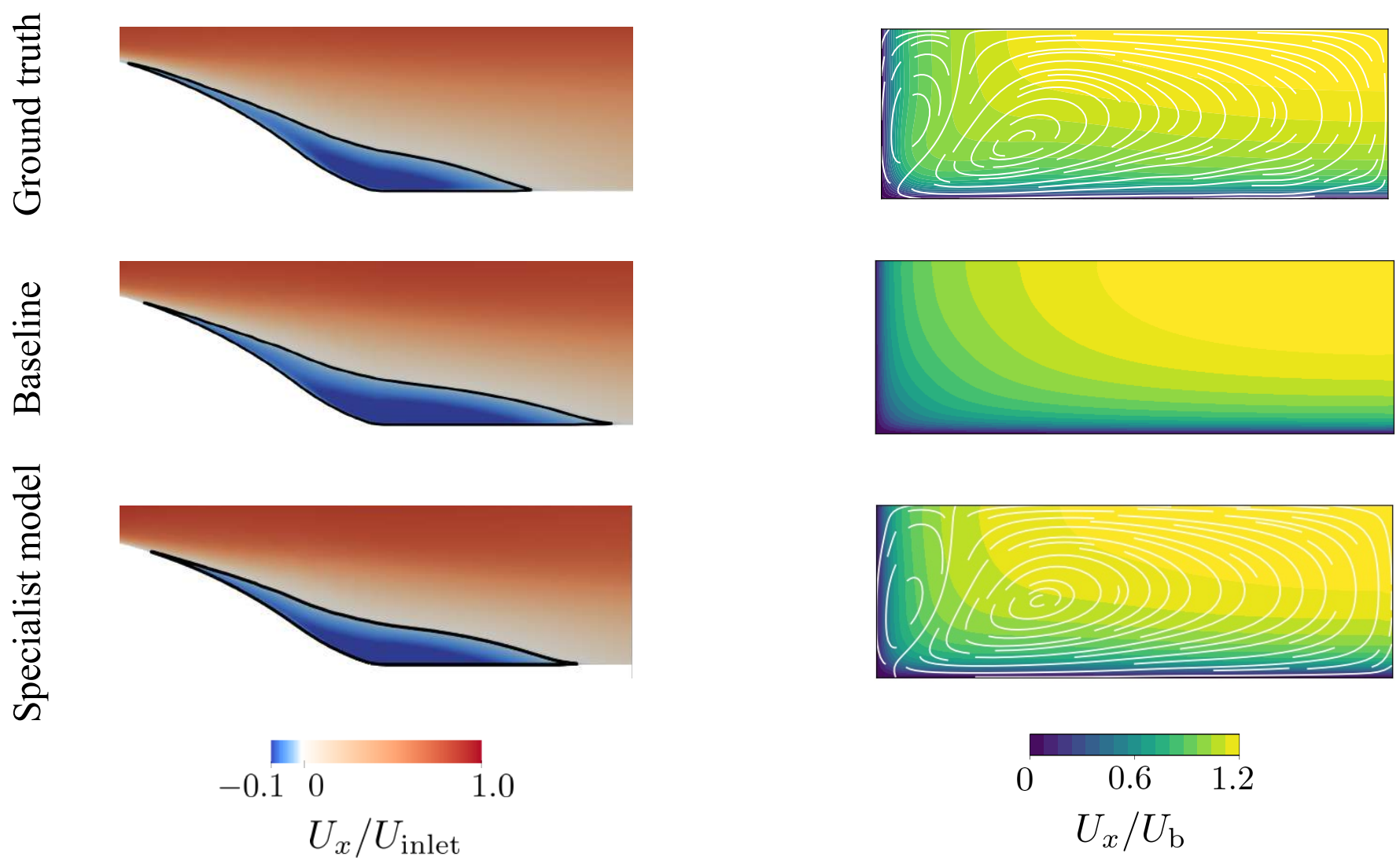

(a) Curved step (b) Rectangular duct (AR=3)

Figure S39: Fine-tuning performance of the **specialist model for secondary flows with separation** against the ground truth and the baseline k–ω model. Separation prediction results are shown for (a) flow over a **curved step**, and in-plane secondary flow predictions are shown for (b) flow through a **rectangular duct** with aspect ratio AR = 3. The results for the rectangular duct with aspect ratio AR = 10 are omitted for brevity. For the curved step, contours of the non-dimensional streamwise velocity U_x/U_{inlet} are shown, where the black contour line ($U_x = 0$) marks the separation boundary and highlights differences in separation extent and reattachment location. For the rectangular duct, cross-sectional contours of U_x/U_{b} overlaid with streamlines illustrate the corner-vortex structures and secondary circulations.

Test results on flows within fine-tuning categories The specialist model generalizes well to separated and secondary flows beyond those included in the fine-tuning set. Its performance on these cases is summarized in Fig. S40. Across secondary flow configurations, i.e., square and rectangular ducts, the specialist model consistently achieves higher prediction accuracy than the unified foundation model (see Fig. S23). For separated flows, it generally maintains, and in some cases slightly improves upon, the accuracy inherited from the unified foundation model. Notably, the 42 mm bump case exhibits a clear performance gain, while the periodic hill and hump cases show accuracy comparable to that of the unified foundation model.

Test results on three-dimensional diffuser The specialist model improves prediction accuracy for the asymmetric diffuser, a three-dimensional flow in which secondary flow and separation are strongly coupled. Streamwise velocity fields and separation patterns at multiple downstream locations (Fig. S41) show how the specialist model corrects the baseline separation topology. In the ground truth, separation originates

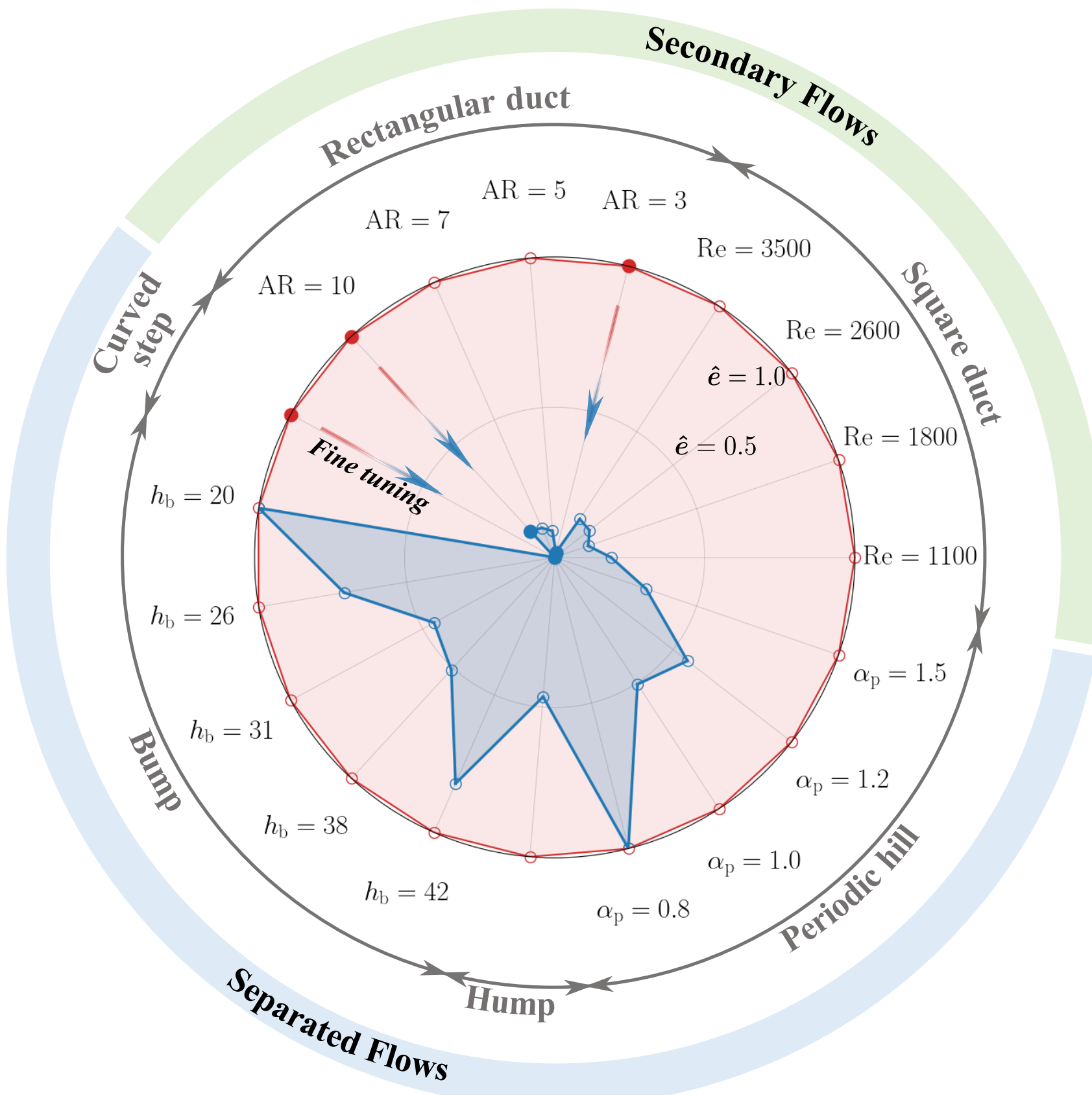

Figure S40: Performance evaluation of the **specialist model for secondary flows with separation** across **separated and secondary flows**, summarized in a radar chart and compared with the baseline k–ω model. The evaluated cases include canonical secondary flows (square and rectangular ducts) and separated flows (curved step, bump, periodic hill, and hump), covering a broad range of geometric and flow conditions. The radial coordinate represents the normalized prediction error $\hat{e}$, where the red polygon with markers ($\hat{e} = 1$) corresponds to the baseline model and the blue filled polygon corresponds to the specialist model. Arrows indicate the fine-tuning cases, and for most remaining test cases, the blue polygon lies inside the red polygon, indicating improved performance of the specialist model.

along the upper wall and grows downstream while remaining attached to the inclined side wall. The baseline model instead predicts extensive separation along the inclined side wall and fails to reproduce the dominant upper-wall separation. The specialist model suppresses this spurious side-wall separation and shifts the separation line to the upper wall, improving agreement with the reference topology. The unified foundation model exhibits intermediate behavior: at downstream stations ($x/h_\mathrm{d} = 12$ and 15), it partially strengthens the upper-wall separation and weakens side-wall separation, but still departs from the ground-truth separation pattern. Complementary spanwise cross sections near the inclined side wall (Fig. S42) further confirm that the specialist model best captures the downstream propagation of upper-wall separation and its reattachment at the diffuser exit, outperforming both the baseline and unified foundation models.

Overall, the specialist model for secondary flows with separation improves predictive performance across both secondary-flow and separated-flow categories and effectively captures their interaction. Its performance on the three-dimensional diffuser, including the suppression of spurious inclined side-wall separation and improved prediction of upper-wall separation, suggests improved generalization ability beyond the training categories.

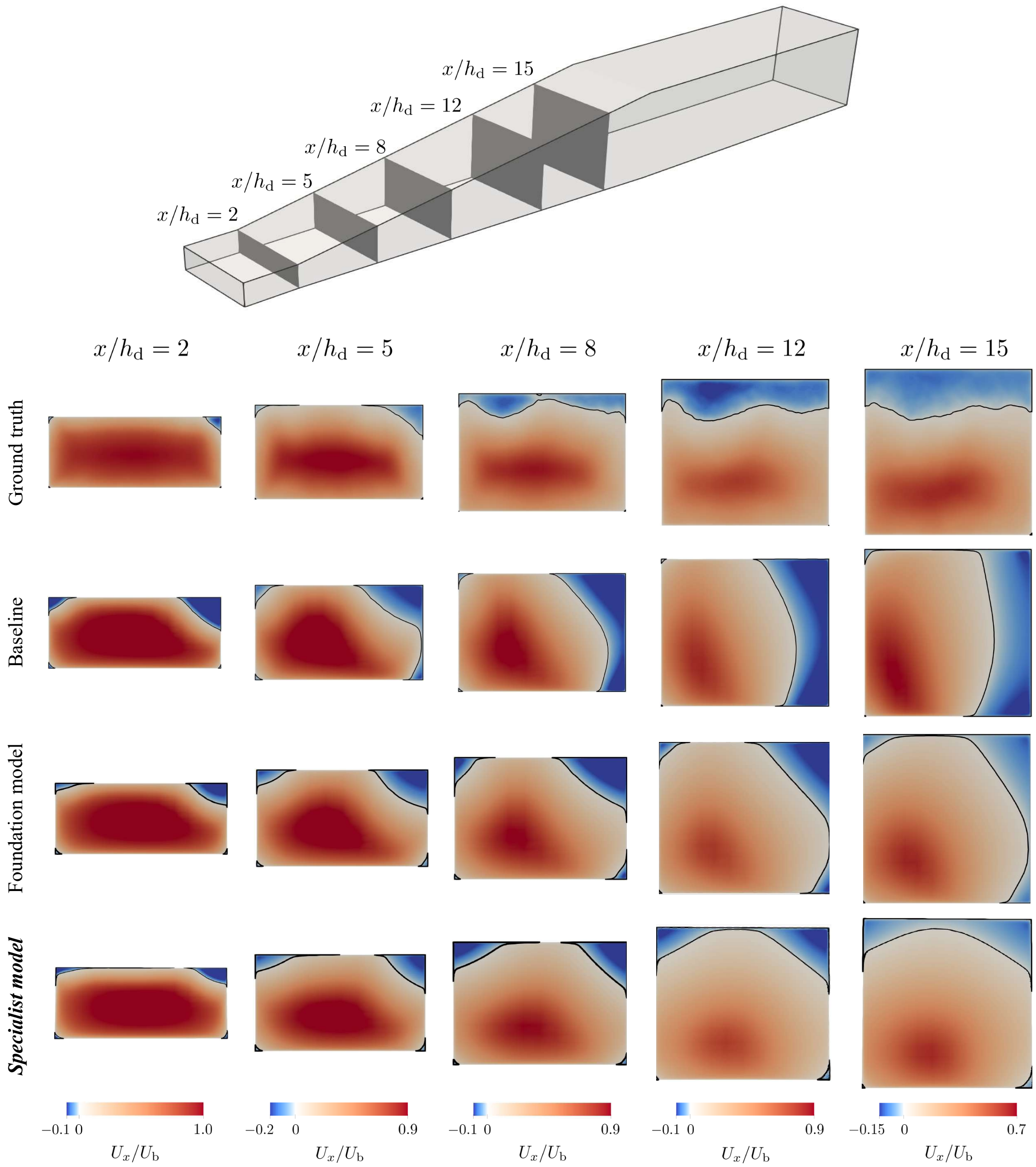

Figure S41: Performance evaluation of the **specialist model for secondary flows with separation** on the **three-dimensional diffuser** against the unified foundation model, the baseline k–ω model, and the ground truth (obtained from DNS mean flow field [69]). Separation predictions at five streamwise cross-sections are extracted at $x/h_\mathrm{d} = 2$, 5, 8, 12, and 15, where h_d denotes the diffuser inlet height. Contours represent the normalized streamwise velocity U_x/U_b, and the $U_x = 0$ isoline marks the separated regions.

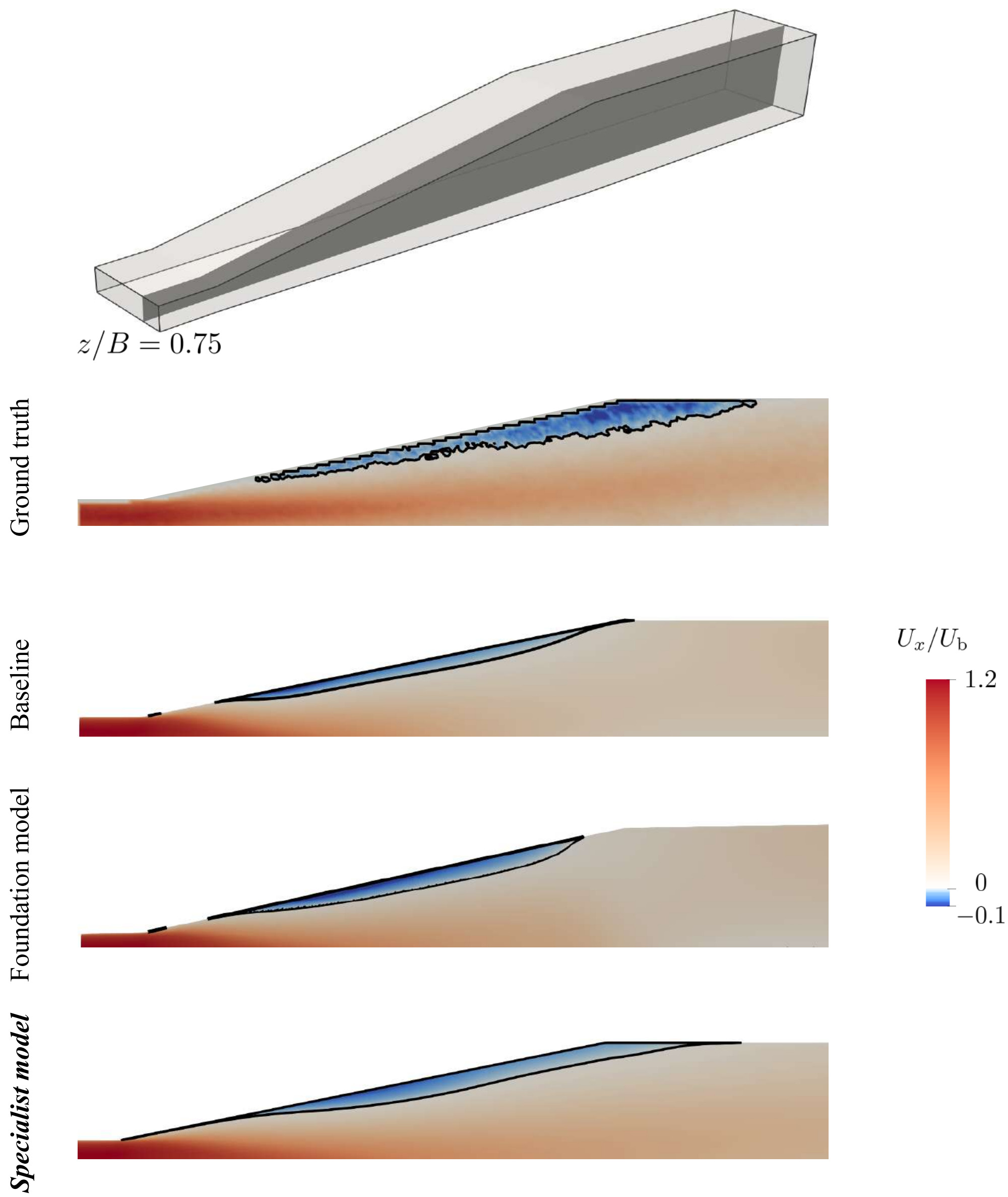

Figure S42: Performance evaluation of the **specialist model for secondary flows with separation** on the **3D diffuser** against the unified foundation model, the baseline k–ω model, and the ground truth (obtained from experiments [65]). Upper-wall separations on a spanwise cross-section at $z/B = 0.75$, where B is the diffuser inlet width, are plotted. Contours represent the streamwise velocity U_x/U_b, with the $U_x = 0$ isoline delineating the separated region.

References

1. Liu Z, Wang H, Zhao Z and Xiao H. Unified data-driven turbulence modeling framework. `https://github.com/ITLR-DDSim/unified-turbulence-modeling` 2025.

2. Slotnick JP, Khodadoust A, Alonso JJ, Darmofal DL, Gropp WD, Lurie EA and Mavriplis DJ. CFD vision 2030 study: A path to revolutionary computational aerosciences. Technical Report NASA/CR-2014-218178, NASA Langley Research Center, Hampton, VA 2014.

3. Lumley JL. Toward a turbulent constitutive relation. *J Fluid Mech* 1970; **41**:413–434.

4. Girimaji SS. Turbulence closure modeling with machine learning: A foundational physics perspective. *New J Phys* 2024; **26**:071201.

5. Xiao H and Cinnella P. Quantification of model uncertainty in RANS simulations: A review. *Prog Aerosp Sci* 2019; **108**:1–31.

6. Duraisamy K, Iaccarino G and Xiao H. Turbulence modeling in the age of data. *Annual Review of Fluid Mechanics* 2019; **51**:357–377.

7. Rumsey CL and Coleman GN. NASA symposium on turbulence modeling: Roadblocks, and the potential for machine learning. Technical Report NASA/TM-20220015595, NASA Langley Research Center, Hampton, VA 2022.

8. Speziale CG. On nonlinear k-l and k-ε models of turbulence. *J Fluid Mech* 1987; **178**:459–475.

9. Gatski TB and Speziale CG. On explicit algebraic stress models for complex turbulent flows. *J Fluid Mech* 1993; **254**:59–78.

10. Pope SB. A more general effective-viscosity hypothesis. *J Fluid Mech* 1975; **72**:331–340.

11. Ling J, Kurzawski A and Templeton J. Reynolds averaged turbulence modelling using deep neural networks with embedded invariance. *J Fluid Mech* 2016; **807**:155–66.

12. Weatheritt J and Sandberg R. A novel evolutionary algorithm applied to algebraic modifications of the RANS stress–strain relationship. *J Comput Phys* 2016; **325**:22–37.

13. Schmelzer M, Dwight RP and Cinnella P. Data-driven deterministic symbolic regression of nonlinear stress-strain relation for RANS turbulence modelling. 48th AIAA Fluid Dynamics Conference, Paper 2018-2900, Atlanta, GA 2018.

14. Wang JX, Wu JL and Xiao H. Physics-informed machine learning approach for reconstructing Reynolds stress modeling discrepancies based on DNS data. *Phys Rev Fluids* 2017; **2**:034603.

15. Wu JL, Xiao H and Paterson E. Physics-informed machine learning approach for augmenting turbulence models: A comprehensive framework. *Phys Rev Fluids* 2018; **3**:074602.

16. Parish EJ and Duraisamy K. A paradigm for data-driven predictive modeling using field inversion and machine learning. *J Comput Phys* 2016; **305**:758–774.

17. Launder BE, Reece GJ and Rodi W. Progress in the development of a Reynolds-stress turbulence closure. *J Fluid Mech* 1975; **68**:537–566.

18. Han J, Zhou XH and Xiao H. An equivariant neural operator for developing nonlocal tensorial constitutive models. *J Computational Physics* 2023; **488**:112243.

19. Zhou XH, Han J and Xiao H. Frame-independent vector-cloud neural network for nonlocal constitutive modeling on arbitrary grids. *Comput Methods Appl Mech Eng* 2022; **388**:114211.

20. Luther J and Jenny P. Non-linear super-stencils for turbulence model corrections. *Communications Physics* 2025; **8**:233.

21. Alaya E, Grabe C and Eisfeld B. Evolutionary algorithm applied to differential Reynolds stress model for turbulent boundary layer subjected to an adverse pressure gradient. AIAA Aviation 2022 Forum, Paper 2022-3337, Chicago, IL 2022.

22. Taghizadeh S, Witherden FD and Girimaji SS. Turbulence closure modeling with data-driven techniques: physical compatibility and consistency considerations. *New J Phys* 2020; **22**:093023.

23. Taghizadeh S, Witherden FD, Hassan YA and Girimaji SS. Turbulence closure modeling with data-driven techniques: Investigation of generalizable deep neural networks. *Phys Fluids* 2021; **33**.

24. Spalart P. An old-fashioned framework for machine learning in turbulence modeling. arXiv:2308.00837, 2023.

25. Fang Y, Zhao Y, Waschkowski F, Ooi AS and Sandberg RD. Toward more general turbulence models via multicase computational-fluid-dynamics-driven training. *AIAA J* 2023; **61**:2100–2115.

26. Cherroud S, Merle X, Cinnella P and Gloerfelt X. Space-dependent aggregation of stochastic data-driven turbulence models. *J Comput Phys* 2025; **527**:113793.

27. Buchanan T, Lăcătuş M, West A and Dwight RP. Data-driven RANS closures using a relative importance term analysis based classifier for 2D and 3D separated flows. *Comput Fluids* 2026; **305**:106899.

28. Rumsey CL, Coleman GN and Wang L. In search of data-driven improvements to RANS models applied to separated flows. AIAA SciTech Forum, Paper 2022-0937, San Diego, CA 2022.

29. Wu C, Zhang S and Zhang Y. Development of a generalizable data-driven turbulence model: Conditioned field inversion and symbolic regression. *AIAA Journal* 2025; **63**:687–706.

30. Gand F, Deck S, Brunet V and Sagaut P. Flow dynamics past a simplified wing body junction. *Physics of Fluids* 2010; **22**:115111.

31. Menter FR and Matyushenko A. Generalized k–ω (GEKO) two-equation turbulence model. *AIAA J* 2025; **63**:4590–4606.

32. Zhang XL, Xiao H, Jee S and He G. Physical interpretation of neural network-based nonlinear eddy viscosity models. *Aerosp Sci Technol* 2023; **142**:108632.

33. Ling J and Templeton J. Evaluation of machine learning algorithms for prediction of regions of high Reynolds-averaged Navier–Stokes uncertainty. *Physics of Fluids* 2015; **27**.

34. Wilcox DC. *Turbulence Modeling for CFD.* DCW Industries, La Canada, CA, 1998, second edition.

35. Pope SB. *Turbulent Flows.* Cambridge University Press, Cambridge, 2000.

36. Kok JC. Resolving the dependence on freestream values for the k-ω turbulence model. *AIAA J* 2000; **38**:1292–1295.

37. Cazalbou J, Spalart PR and Bradshaw P. On the behavior of two-equation models at the edge of a turbulent region. *Phys Fluids* 1994; **6**:1797–1804.

38. Wu C, Zhang S, Guo C and Zhang Y. Data-driven turbulence modeling for separated flows considering nonlocal effect. *AIAA Journal* 2025; **63**:5326–5343.

39. Kim J, Moin P and Moser R. Turbulence statistics in fully developed channel flow at low Reynolds number. *J Fluid Mech* 1987; **177**:133–66.

40. Xiao H, Wu JL, Laizet S and Duan L. Flows over periodic hills of parameterized geometries: A dataset for data-driven turbulence modeling from direct simulations. *Comput Fluids* 2020; **200**:104431.

41. Bi K, Xie L, Zhang H, Chen X, Gu X and Tian Q. Accurate medium-range global weather forecasting with 3D neural networks. *Nature* 2023; **619**:533–538.

42. Callaway E. Deepmind's new AlphaGenome AI tackles the 'dark matter' in our DNA. *Nature* 2025; **643**:17–18.

43. Kendall A, Gal Y and Cipolla R. Multi-task learning using uncertainty to weigh losses for scene geometry and semantics. *Proceedings of the IEEE Conference on Computer Vision and Pattern Recognition*. 2018 7482–7491.

44. Chen Z, Badrinarayanan V, Lee CY and Rabinovich A. Gradnorm: Gradient normalization for adaptive loss balancing in deep multitask networks. *International Conference on Machine Learning*. PMLR, 2018 794–803.

45. Désidéri JA. Multiple-gradient descent algorithm (MGDA) for multiobjective optimization. *Comptes Rendus Mathématique* 2012; **350**:313–318.

46. Kuhn HW and Tucker AW. Nonlinear programming. Neyman J, editor, *Proceedings of the Second Berkeley Symposium on Mathematical Statistics and Probability*. University of California Press, Berkeley, 1951 481–492.

47. Jaggi M. Revisiting Frank-Wolfe: Projection-free sparse convex optimization. *International Conference on Machine Learning*. PMLR, 2013 427–435.

48. Sener O and Koltun V. Multi-task learning as multi-objective optimization. *Advances in Neural Information Processing Systems*, volume 31, 525–536. Curran Associates, Inc., 2018.

49. Zhang XL, Michelén-Ströfer CA and Xiao H. Regularized ensemble Kalman methods for inverse problems. *J Comput Phys* 2020; **416**:109517.

50. Zhang XL, Xiao H, Luo X and He G. Combining direct and indirect sparse data for learning generalizable turbulence models. *J Comput Phys* 2023; **489**:112272.

51. Bin Y, Huang G, Kunz R and Yang XI. Constrained recalibration of Reynolds-averaged Navier–Stokes models. *AIAA J* 2024; **62**:1434–1446.

52. Zhang XL, Xiao H, Luo X and He G. Ensemble Kalman method for learning turbulence models from indirect observation data. *J Fluid Mech* 2022; **949**:A26.

53. Evensen G, Vossepoel FC and Van Leeuwen PJ. *Data assimilation fundamentals: A unified formulation of the state and parameter estimation problem*. Springer Nature, 2022.

54. Vishny D, Morzfeld M, Gwirtz K, Bach E, Dunbar OR and Hodyss D. High-dimensional covariance estimation from a small number of samples. *Journal of Advances in Modeling Earth Systems* 2024; **16**:e2024MS004417.

55. Jespersen DC, Pulliam TH and Childs ML. OVERFLOW turbulence modeling resource validation results. Technical Report NAS-2016-01, NASA Ames Research Center, Moffett Field, CA 2016.

56. Somers DM. Design and experimental results for the S809 airfoil. Technical Report NREL/SR-440-6918, National Renewable Energy Laboratory, Golden, CO 1997.

57. Ladson CL. Effects of independent variation of Mach and Reynolds numbers on the low-speed aerodynamic characteristics of the NACA 0012 airfoil section. Technical Memorandum NASA-TM-4074, NASA Langley Research Center, Hampton, VA 1988.

58. Bentaleb Y, Lardeau S and Leschziner MA. Large-eddy simulation of turbulent boundary layer separation from a rounded step. *J Turbul* 2012; N4.

59. Matai R and Durbin P. Large-eddy simulation of turbulent flow over a parametric set of bumps. *J Fluid Mech* 2019; **866**:503–25.

60. Seifert A and Pack LG. Active flow separation control on wall-mounted hump at high reynolds numbers. *AIAA journal* 2002; **40**:1363–1372.

61. Pinelli A, Uhlmann M, Sekimoto A and Kawahara G. Reynolds number dependence of mean flow structure in square duct turbulence. *J Fluid Mech* 2010; **644**:107–122.

62. Vinuesa R, Schlatter P and Nagib H. Secondary flow in turbulent ducts with increasing aspect ratio. *Phys Rev Fluids* 2018; **3**:054606.

63. Bridges J and Wernet MP. Establishing consensus turbulence statistics for hot subsonic jets. 16th AIAA/CEAS Aeroacoustics Conference, Paper 2010-3751, Stockholm, Sweden 2010.

64. Lienhart H and Becker S. Flow and turbulence structure in the wake of a simplified car model. *SAE Transactions* 2003; 785–796.

65. Cherry EM, Elkins CJ and Eaton JK. Geometric sensitivity of three-dimensional separated flows. *Int J Heat Fluid Flow* 2008; **29**:803–811.

66. Evans AN, Lacy DS, Smith I and Rivers MB. Test summary of the NASA semi-span high-lift common research model at the QinetiQ 5-metre low-speed wind tunnel. AIAA Aviation 2020 Forum, Paper 2020-2770.

67. Singh AP and Duraisamy K. Using field inversion to quantify functional errors in turbulence closures. *Physics of Fluids* 2016; **28**:045110.

68. Goc KA, Moin P, Bose ST and Clark AM. Wind tunnel and grid resolution effects in large-eddy simulations of the high-lift common research model. *Journal of Aircraft* 2024; **61**:267–279.

69. Ohlsson J, Schlatter P, Fischer P and Henningson D. DNS of separated flow in a three-dimensional diffuser by the spectral-element method. *J Fluid Mech* 2010; **650**:307–318.
